# Superalkali–Alkalide Interactions and Ion Pairing in Low-Polarity Solvents

**DOI:** 10.1021/jacs.1c00115

**Published:** 2021-03-04

**Authors:** René Riedel, Andrew G. Seel, Daniel Malko, Daniel P. Miller, Brendan T. Sperling, Heungjae Choi, Thomas F. Headen, Eva Zurek, Adrian Porch, Anthony Kucernak, Nicholas C. Pyper, Peter P. Edwards, Anthony G. M. Barrett

**Affiliations:** † Department of Chemistry, Imperial College London, Molecular Sciences Research Hub, White City Campus, Wood Lane, London W12 0BZ, U.K.; ‡ Department of Physics and Astronomy, University College London, Gower Street, London WC1E 6BT, U.K.; § Inorganic Chemistry Laboratories, University of Oxford, Park Royal Road, Oxford OX1 3QR, U.K.; ∥ Department of Chemistry, Hofstra University, 106 Berliner Hall, Hempstead, New York 11549, United States; ⊥ School of Engineering, Cardiff University, Cardiff CF24 3AA, U.K.; # ISIS Neutron and Muon Source, Science and Technology Facilities Council, Rutherford Appleton Laboratory, Harwell Campus, Didcot OX11 0QX, U.K.; × Department of Chemistry, State University of New York at Buffalo, 777 Natural Sciences Complex, Buffalo, New York 14260-3000, United States; + University Chemical Laboratory, Lensfield Road, Cambridge CB2 1EW, U.K.

## Abstract

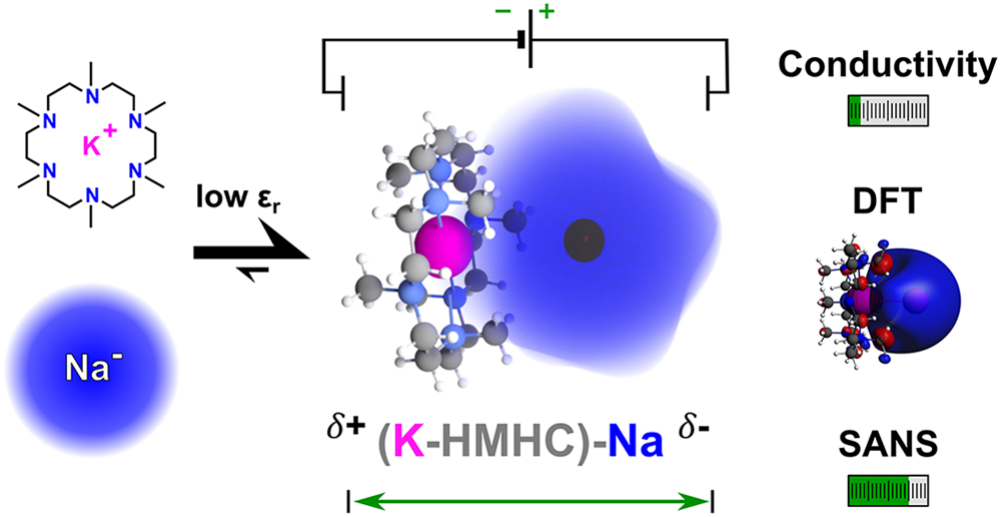

The nature of anionic alkali metals in solution is traditionally thought to be “gaslike” and unperturbed. In contrast to this noninteracting picture, we present experimental and computational data herein that support ion pairing in alkalide solutions. Concentration dependent ionic conductivity, dielectric spectroscopy, and neutron scattering results are consistent with the presence of superalkali–alkalide ion pairs in solution, whose stability and properties have been further investigated by DFT calculations. Our temperature dependent alkali metal NMR measurements reveal that the dynamics of the alkalide species is both reversible and thermally activated suggesting a complicated exchange process for the ion paired species. The results of this study go beyond a picture of alkalides being a “gaslike” anion in solution and highlight the significance of the interaction of the alkalide with its complex countercation (superalkali).

## Introduction

Anionic forms of the electropositive Group I metals, with the exception of lithium, can be generated in condensed phases.^1^ Termed alkalides, these monoanions are chemically highly reducing and possess a diffuse, closed-shell *n*s^2^ configuration, resulting in an exceptionally high electronic polarizability. The formation and stabilization of alkalide species requires stringent chemical environments and involves either a disproportionation or the reduction of one elemental alkali metal by another.^2^ The dissolution and reduction process is facilitated by strong stabilization of the alkali cation by preorganized complexants such as crown ethers and cryptands.^3^ This enables alkali metals to be dissolved in even weakly polar solvents such as tetrahydrofuran (THF) by formation of alkalide anions that persist in the absence of any better electron receptor, as with ammonia or small amines (metal–ammonia solutions^4−6^ and solutions containing solvated electrons^7,8^), functional groups in organic or organometallic molecules (the dissolving metal reduction), or simply a different, more reducible metal. Cryogenic temperatures are also necessitated to avoid reduction and decomposition of solvent.

Perhaps the most puzzling aspect of alkalides, which has persisted almost from the time of their discovery, is an understanding of the precise nature of their diffuse *n*s^2^ orbital in solution. The NMR signatures of an alkalide species in solution, and in the crystalline solids they form, are significantly shielded and exhibit an exceptionally narrow line width. Considering that the alkali metals all possess quadrupolar nuclei, these features have been ascribed to the high shielding and high symmetry of an unperturbed “gaslike” anion in solution, with little to no interaction with its local environment.^9−14^ However, the high polarizability of the alkalides and ready electron dissociation into solvated-electron species with increasing solvent polarity imply that the genuine alkalide could be significantly perturbed in certain cases. Indeed, the “gaslike” picture of alkalides in solution has recently been questioned by *ab initio* calculations, which have instead provided two insights in favor of a picture of an alkalide interacting with its environment: First, it was suggested that the most stable species were formed via the association of the alkalide anion with solvated/complexed alkali cations in a known alkalide-forming solvent, 1,2-ethylenediamine,^15^ and it was shown that the simulated absorption spectra for such interacting species in the visible and ultraviolet ranges were in agreement with experimental observations. The complexed alkali metal cations have been termed “superalkali” cations because their LUMOs are highly expanded but retain similar symmetries to those of the uncomplexed alkali metal cations and they are able to accept electron density from the alkalide in a weak donor–acceptor sense. Second, molecular dynamics simulations on explicitly solvated sodide (Na^–^) ions suggested that the expanded 3s^2^ orbital is perturbed by its environment, but the NMR response for the sodium nucleus is negligibly affected, despite its quadrupolar nature.^16^

**Figure 1:**
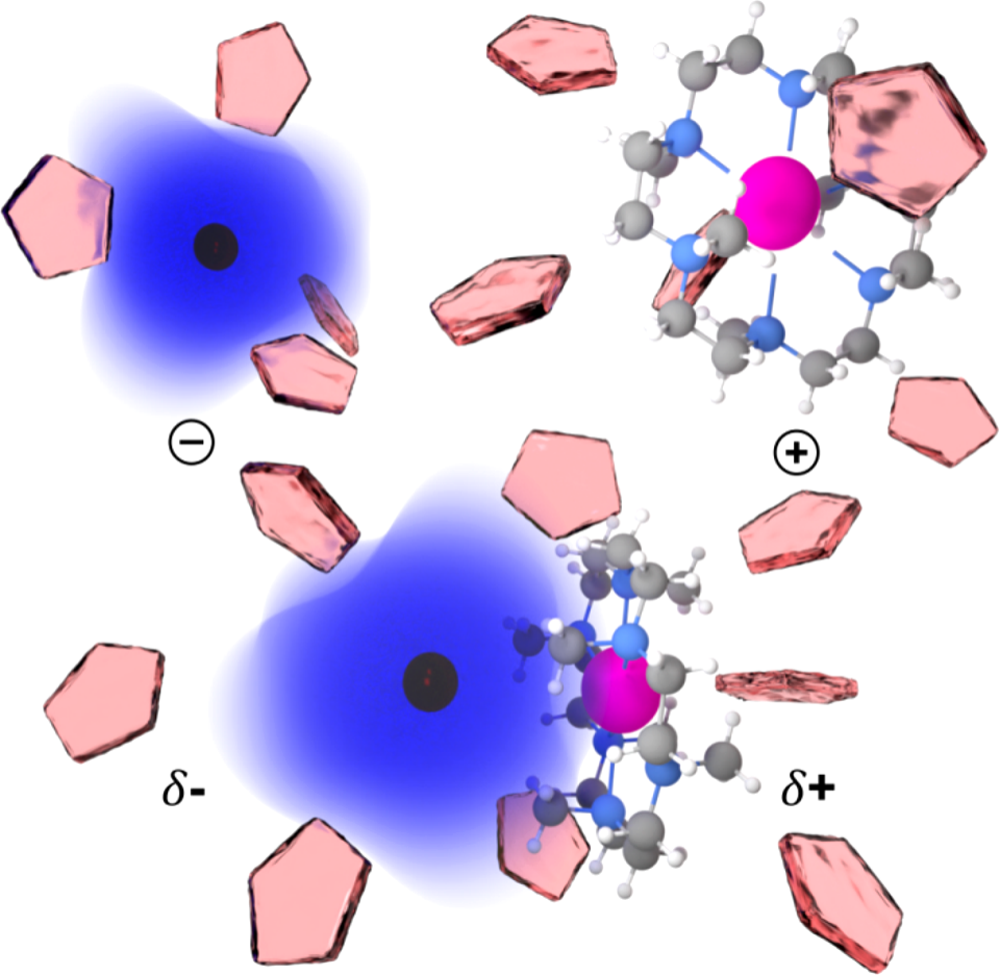
Illustration of potential components of a sodide solution in HMHC/THF. Separately solvated complexed potassium (pink) cation (top right) and alkalide anion (top left) with its diffuse 3s^2^ valence orbital (blue) and a contact ion pair (bottom) in a medium of THF molecules (red) are indicated.

Here, we provide experimental evidence that alkalides interact with their environment through the formation of ion paired species in solution (see Figure 1). Further support is provided by density functional theory (DFT) calculations that suggest a nature beyond that of classic ion associates.^17^ Such weakly covalent interactions between the alkalide and the counter superalkali cation reflect a subtle chemistry for alkalides, which has previously not been reported. As such, our findings have implications for future control of alkalide properties and their potential use in photo- and electrochemical applications.

## Experimental Section

### Synthesis

Full experimental details and characterization regarding the preparation of HMHC are provided in the Supporting Information. Crude HMHC and commercially available 15-crown-5 were purified by Kugelrohr distillation from NaK, kept under an argon atmosphere and handled inside an argon glovebox.

### Ionic Conductivity Measurements

Impedance measurements were carried out using a potentiostat Gamry Reference 600 and using platinized platinum electrodes (cell constant 0.98 cm). The Walden product was calculated using the shear viscosity of metal-free solutions of 15-crown-5 in THF at all relevant concentrations as an approximation of the viscosity of metal solutions. The shear viscosity was determined over the temperature range of 15–40 °C using a RheoSense m-VROC viscometer. In a typical experiment, NaK was added in small portions (<20 mg) to a solution of 0.06 M 15-crown-5 or 0.03 M HMHC in dry THF at 243 K while the conductivity of the mixture was monitored in set intervals of 0.5 min. Macrocycle concentration was doubled when the concentration of dissolved metal reached approximately 70% saturation to maintain a sufficient metal dissolution rate. The ratio between metal and macrocycle concentration at a given metal concentration was found to have no effect on the conductivity.

### Small Angle Neutron Scattering (SANS)

SANS spectra were collected at the neutron diffractometer NIMROD^18^ at the ISIS Neutron and Muon Source Facility, U.K. Samples were prepared by mixing of all the components (HMHC (1 equiv) or 15-crown-5 (2 equiv), *d*_8_-THF, NaK (*n*/*n* 1:1, 3 equiv)) in a quartz NMR tube inside an argon glovebox with a cryogenic and inert atmosphere being maintained. Samples were then warmed to 240 K, and equilibrated for 10 h prior to measurement to ensure complete metal dissolution up to a point of saturation. Data were reduced using standardized procedures within the GudrunN software.^19^ Density values were precisely determined for all relevant metal-free complexant solutions in protiated THF using a 4 place digital LiquiPhysics Excellence density meter DM40 over the temperature range of 273–303 K. Density values at 243 K were determined by linear extrapolation (Details are provided in the Supporting Information.).

### Density Functional Theory

The DFT calculations used the Perdew–Burke–Ernzerhof (PBE) functional^20^ with the Amsterdam Density Functional (ADF)^21−23^ software package. TZP basis sets from the ADF basis set library were used for the Na, K, C, N, and O atoms, while the QZ3P + 1 diffuse function basis set was used for the H atoms.^24^ The core electronic states were kept frozen for all atoms except Na and K. Further computational details are provided in the Supporting Information, section S7.

### NMR Spectroscopy

^23^Na NMR spectra were acquired at 106 MHz on a Bruker DRX-400 spectrometer. ^39^K NMR spectra were acquired at 28 MHz on a Bruker Avance 600 MHz NMR spectrometer. Chemical shifts are reported as δ-values in ppm relative to the cation signal from external aqueous solutions of the respective chloride salt at room temperature. Samples were prepared by addition of all components (complexant HMHC (1 equiv) or 15-crown-5 (2 equiv), *d*_8_-THF, NaK (3 equiv)) to an oven- and flame-dried borosilicate NMR tube with a J. Young valve inside an argon glovebox. The sealed tubes were removed from the glovebox and cooled to 195 K before exposure of the metal alloy to the solution. The samples were stored at 195 K and warmed to 240 K for 10 h in preparation for the respective NMR experiment to ensure complete metal dissolution up to a point of saturation. The probe of the NMR spectrometer was cooled to 200 K prior to quick sample loading. The steady reduction in signal intensity upon thermal cycling is reversible, while taking into account a slight loss of intensity over time due to minor decomposition processes. All measurements were corrected for any loss in signal intensity due to a shift of the Boltzmann distribution of spin states.

## Results and Discussion

### Design and Control of Chemical Composition

**Figure 2:**
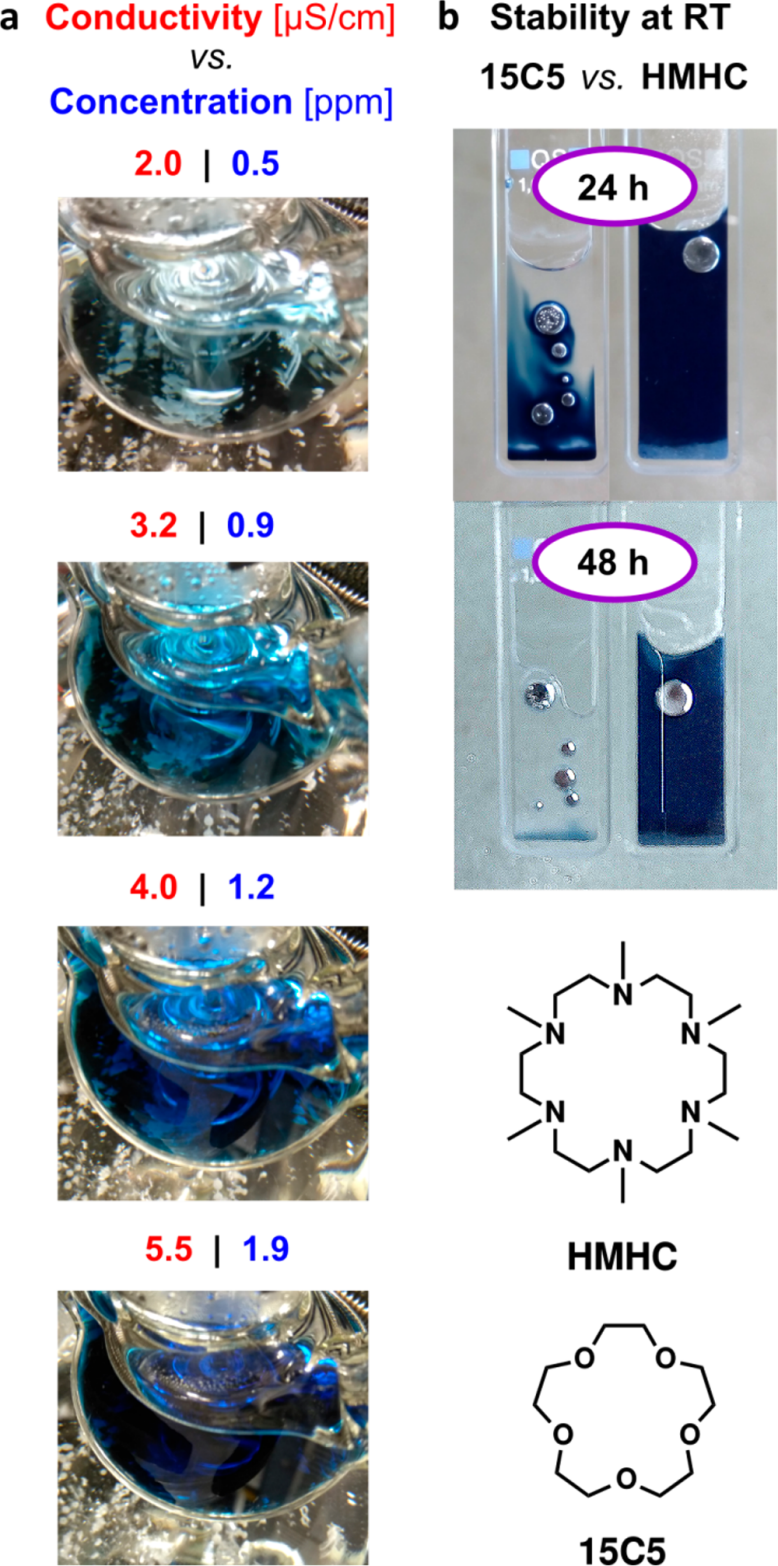
Alkalide solutions in low-polarity solvent tetrahydrofuran (THF). (a) Relation between concentration, conductivity, and color intensity of characteristically blue sodide solutions in 50 mL of THF at extremely high dilution. (b) Stability of solutions of NaK in 40 mM 15-crown-5/THF (left) and 20 mM HMHC/THF (right) in the presence of NaK after storage at room temperature for 24 h (top) and 48 h (bottom). Structures of aza-crown HMHC and crown ether 15-crown-5 (15C5) used in this work.

The accurate preparation and investigation of alkalide solutions, especially at concentrations as low as those shown in Figure 2a, requires the use of complexing agents that are resistant to irreversible reductive ring scission. A milestone in the development of more stable alkalide systems was the introduction of per-alkylated polyamine ligands to the field.^25^ This showed that the hexa-aza-crown 1,4,7,10,13,16-hexamethyl-1,4,7,10,13,16-hexaazacyclooctadecane (hexamethyl-hexacyclen, HMHC, see Figure 2b) significantly outperforms the reportedly most stable crown ether 15-crown-5^26^ in its resistance to reductive decomposition in the presence of alkalides. THF was found to be the most suitable organic solvent as it allowed for a comparably rapid metal dissolution and for the preparation of alkalide solutions with exceptionally high metal concentrations, long-lived stability, and persistence. Figure 2b highlights the stark contrast in stability between the metal solutions using HMHC and the more labile complexant 15-crown-5 in THF, which was ascribed to the decreased reactivity of C–N bonds as compared to C–O bonds under strongly reducing conditions.

### Ionic Conductivity and Small Angle Neutron Scattering (SANS)

**Figure 3:**
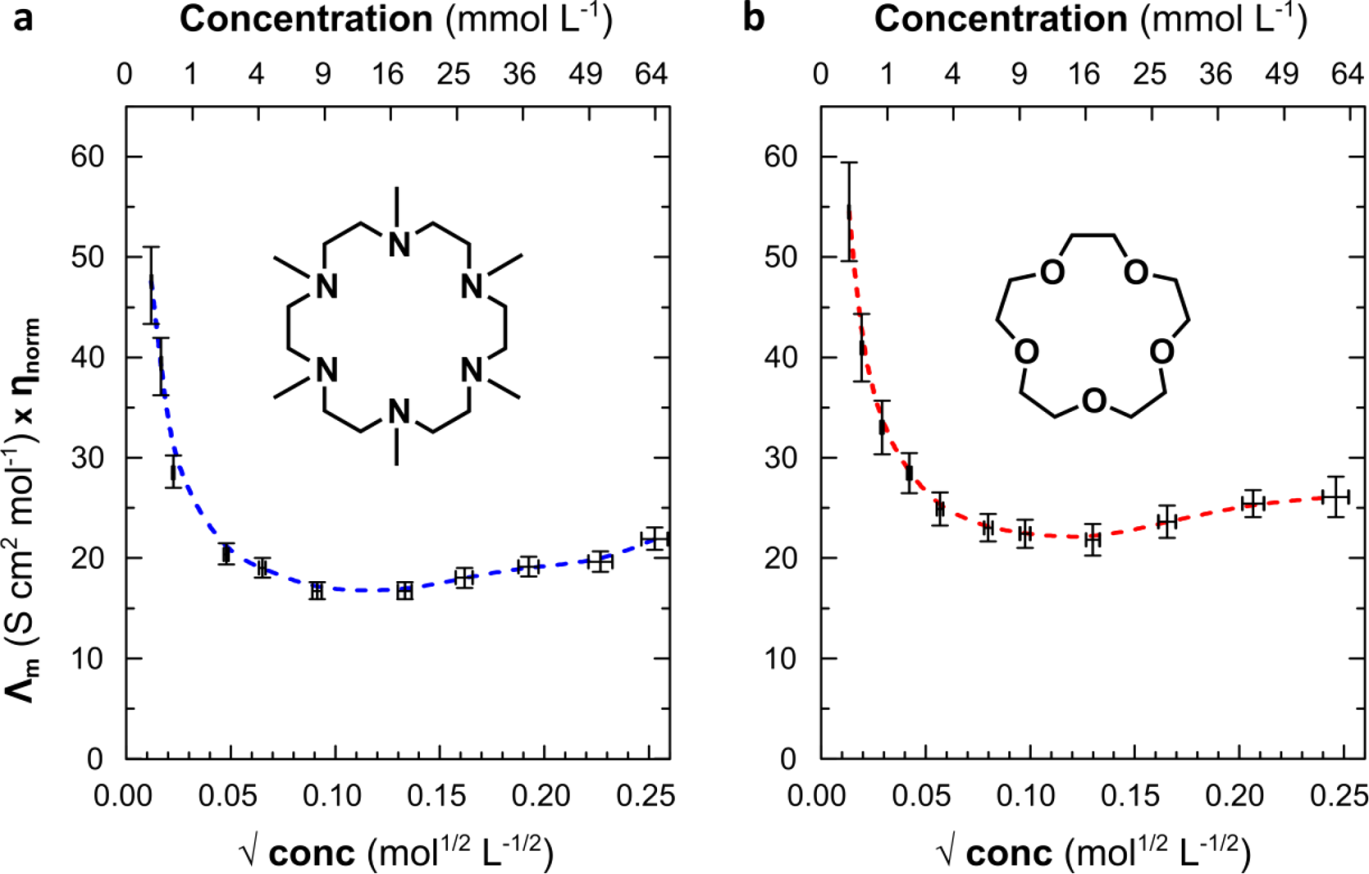
Walden product vs square-root of the concentration of sodide solutions. (a) Concentration dependent Walden product of solutions of NaK in HMHC in THF at 243 K. (b) Concentration dependent Walden product of solutions of NaK in 15-crown-5 in THF at 243 K. Dashed lines represent guides to the eye.

Investigation of the concentration dependence of ionic conductivity of electrolyte solutions is the acknowledged technique to interrogate the nature of ion association in solution. Our experimental methodology allowed for the reproducible preparation of highly dilute metal solutions (Figure 2a) and the accurate determination of their metal content by ion chromatography, enabling the investigation of conductivity across a wide concentration range. Plots of the relationship between concentration and the associated Walden product for solutions of NaK in 15-crown-5/THF and HMHC/THF (Figure 3) show that both exhibit a rapid decrease followed by a slight increase of the derived Walden product with increasing metal concentration. While the decrease in molar conductivity is a familiar consequence of ion association, the presence of a minimum at ∼15 mM distinguishes the Walden product trend of the alkalide systems from that of a classic weak electrolyte. Such conductance minima have been reported for several solutions of electrolytes and ionic liquids in low-polarity solvents^27−37^ and agree with the theory of a feedback between the ion association equilibrium and the overall relative permittivity of mixtures in low-polarity solvents.^31,38−42^ Examination of the frequency-dependent dielectric spectra and the static permittivity over the period of metal dissolution yields results that are consistent with an increase of the overall relative permittivity of the solution due to an increasing number of ion pairs upon metal dissolution (Supporting Information, Figure SI-4).

We employed neutron scattering measurements to examine any structural signatures for such associations. The length scales of the macrocycle-encapsulated cation or the envisaged contact-ion/ion paired superalkali–alkalide correspond to a range in reciprocal space that is intermediary between that probed in small and wide-angle neutron scattering, with intramolecular distances overlapping with intermolecular, solvent–solvent, or solvent–solute distances. The use of a protiated macrocycle and deuterated solvent overcomes this problem due to the large difference in coherent neutron scattering length of the proton (−3.74 fm) and the deuteron (6.67 fm). This enables the macrocyclic complex to behave as a scattering object in the limit of low scattering angle *Q*, and any change to its size/solvation via the association of the alkalide should be apparent. Within the low-*Q* limit, the coherent differential neutron scattering cross section, dσ/dΩ, is given by
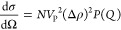
1where *N* is the number of scattering objects of volume *V*_P_, Δρ is the contrast between the scattering-length density of the object and the average scattering-length density of the solvent, and *P*(*Q*) is the form- or shape-factor for the object. This expression holds only for the dilute limit, where there are no significant “object–object” correlations in the solution.

**Figure 4:**
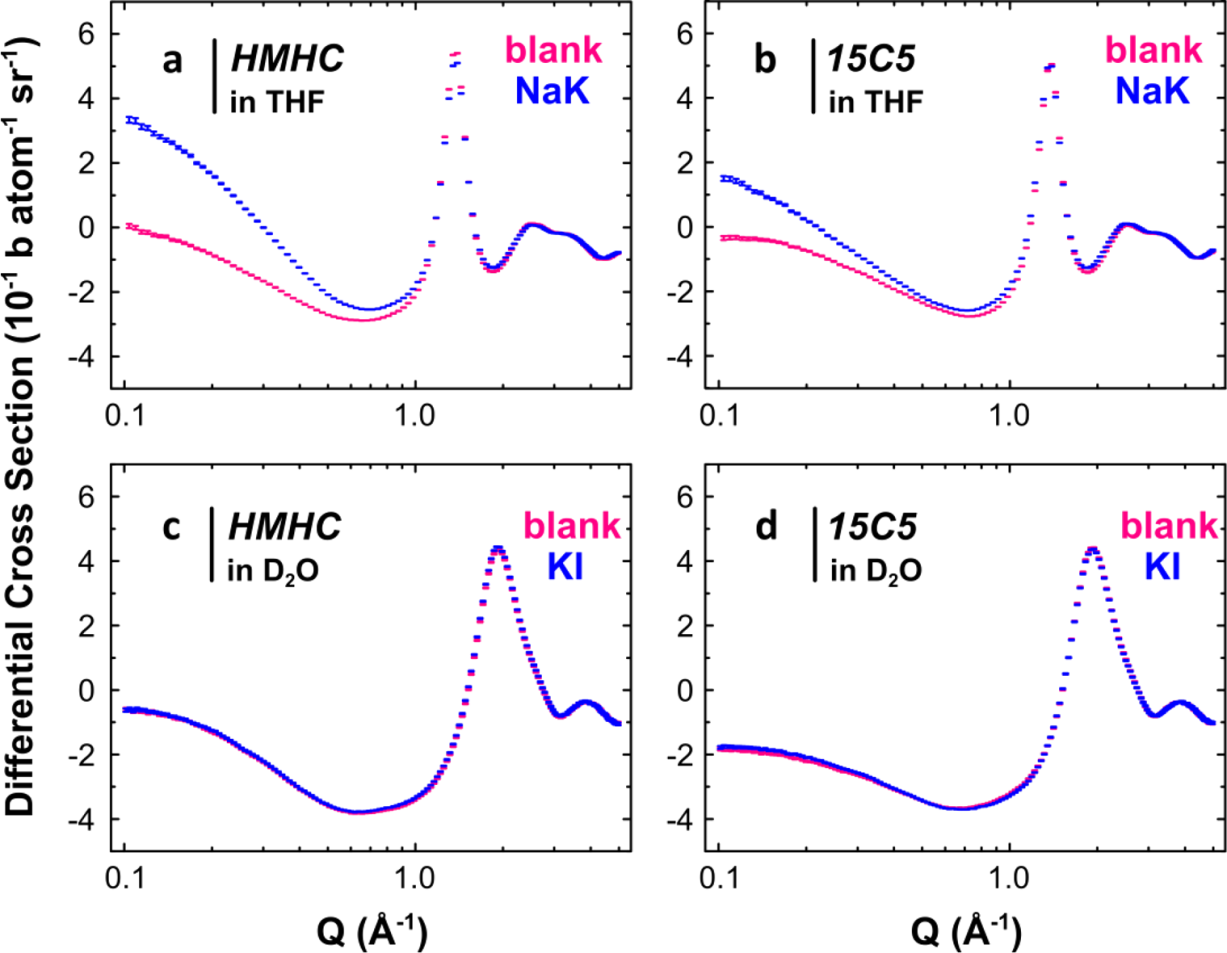
Small angle neutron scattering spectra for macrocycle solutions of both NaK in THF and KI in D_2_O. Coherent differential cross section of blank (pink) and ion-containing (blue) solutions involving HMHC in THF-*d*_8_ (0.05 M, a), HMHC in D_2_O (0.05 M, c), 15-crown-5 in THF-*d*_8_ (0.1 M, b), and 15-crown-5 in D_2_O (0.1 M, d).

Figure 4 presents neutron scattering data for both alkalide solutions (15-crown-5 or HMHC in THF with and without dissolved K^+^ Na^–^) and control solutions of either 15-crown-5 or HMHC in D_2_O both with and without dissolved K^+^ I^–^. These control solutions were chosen to represent fully ion-dissociated systems of separately solvated macrocycle-K^+^ and I^–^. Furthermore, the iodide and sodide anions are almost identical in size and possess similar coherent neutron scattering lengths.

It is apparent from Figure 4 that the alkalide solutions both possess an increase in small-angle intensity whereas the control systems containing iodide do not exhibit such an increase. Importantly, this indicates that the scattering behavior of the macrocycle complex does not change in the control (as expected from simply binding K^+^ with its extremely low neutron scattering length and negligible contribution to scattering from the macrocycle complex), whereas in the alkalide systems the scattering volume and contrast has clearly increased. Simple ellipsoidal form factor models are reported in the Supporting Information in Figure SI-5 and are concordant with an association of the alkalide with the macrocyclic superalkali, effectively increasing the effective volume of the scattering object in solution from that of a solvated macrocycle to a solvated superalkali–alkalide ion pair. Moreover, a larger aggregation of ions in solution would lead to a small angle signal orders of magnitude higher than those witnessed in the alkalide solutions, thus enabling us to rule out any macroscopic phase separation or higher alkalide agglomerates in the solutions.

### *Ab Initio* Calculations

**Figure 5:**
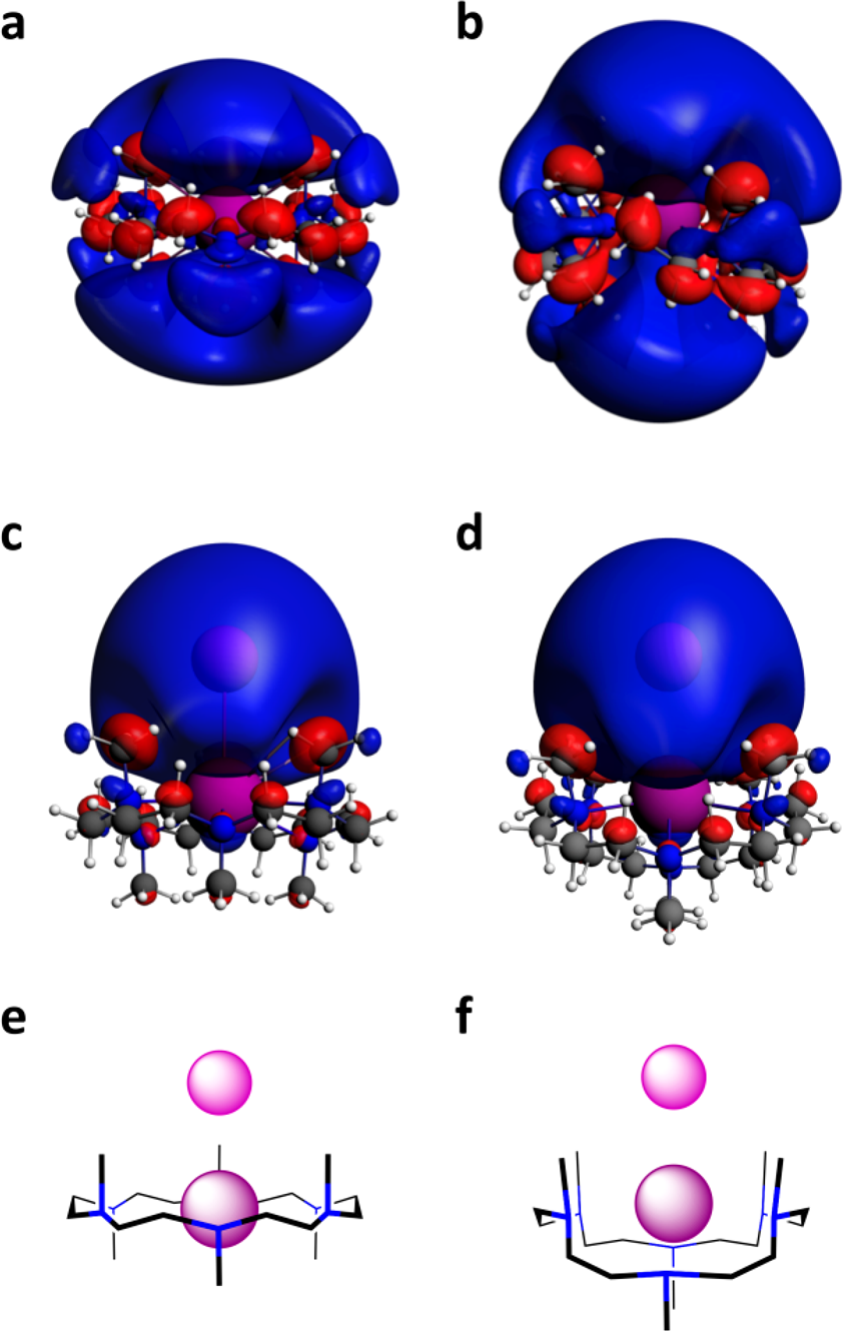
Computed SOMOs/HOMOs of superalkali K-HMHC and superalkali–alkalide (K-HMHC)^δ+^(Na)^δ-^. Isosurfaces (isovalue = ±0.010 au) for superalkali models K-HMHC in the chair (a) and boat (b) conformations and for the superalkali–alkalide models K-HMHC (Na) in the chair (c) and boat (d) conformations. Ion pairs in the chair (e) and boat (f) conformations illustrated in the form of a cartoon.

**Figure 6:**
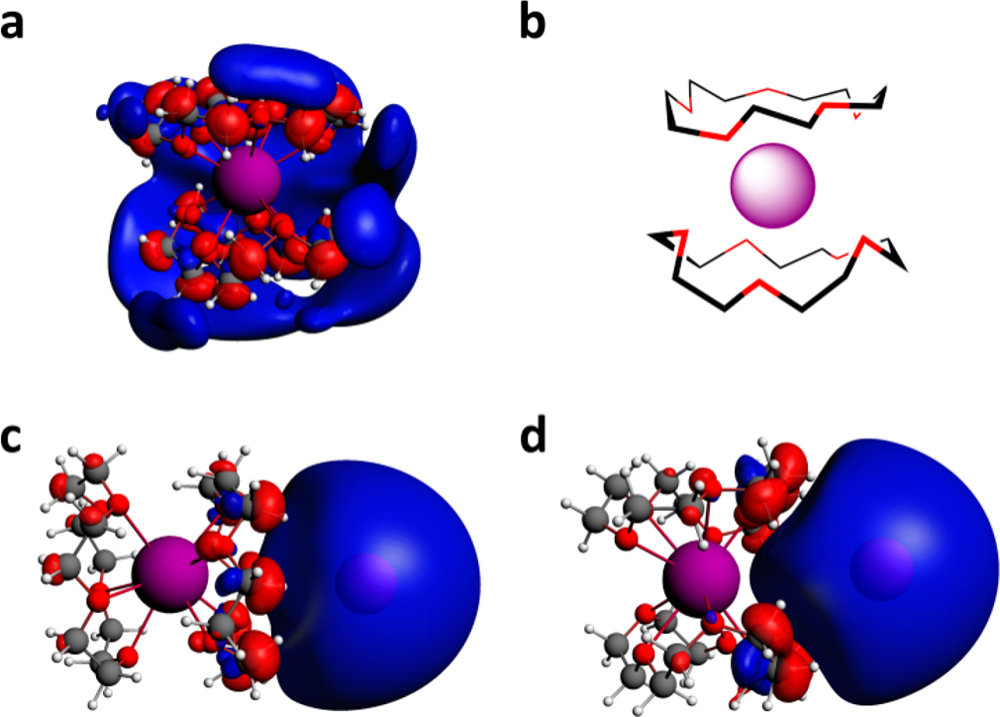
Computed SOMO/HOMOs of superalkali K-15-crown-5_2_ and superalkali–alkalide (K-15-crown-5_2_)^δ+^(Na)^δ-^. Isosurfaces (isovalue = ±0.010 au) for superalkali model K-15-crown-5_2_ (a) and superalkali–alkalide K-15-crown-5_2_ (Na) with the alkalide in the axial (c) and equatorial (d) positions with respect to the sandwich complex (as illustrated in the form of a cartoon (b)).

To assess the possible species that may form in alkalide solutions, DFT calculations were performed on sodium and potassium superalkalis of HMHC and 15-crown-5, superalkali–alkalide ion pairs, and superalkali dimers. As shown in Table 1, K was calculated to interact more strongly than Na with both HMHC and 15-crown-5. The computed binding energies (BEs) suggest that the metal atoms prefer to be coordinated to two 15-crown-5 molecules as opposed to a single complexant (Supporting Information, section S15). In agreement with prior DFT calculations,^15^ the superalkali dimers were found to be less stable than the superalkali–alkalide ion pairs when solvation effects were considered (Supporting Information, section S14). Therefore, in this discussion we focus on the superalkali–alkalide ion pairs whose computed BEs and selected properties are given in Table 1, with optimized geometries and molecular orbital isosurfaces illustrated in Figures 5 and 6. Out of the four metal combinations that are possible, the encapsulated potassium and anionic sodide models, K-HMHC (Na), and K-15-crown-5_2_ (Na), each possess the largest BEs, in agreement with the experimental observation of a predominance of potassium cations and sodide anions.

**Table 1:** Binding Energies and Select Molecular Properties of Superalkali and Superalkali–Alkalide Pair Models from DFT Calculations^a^

	binding energy^b^ (kJ/mol)	M–M distance (Å)	superalkali Hirshfeld charge (e)	alkalide Hirshfeld charge (e)	dipole moment (D)
Superalkali M-15-crown-5_2_ Models					
Na-15-crown-5_2_	–84.5		+0.26		4.5
K-15-crown-5_2_	–109.7		+0.23		2.7
Superalkali-Alkalide Pair M-15-crown-5_2_ (M) Models					
Na-15-crown-5_2_ (Na)	–147.4	5.71	+0.28	–0.45	17.1
K-15-crown-5_2_ (Na)–equatorial	–178.2	5.59	+0.28	–0.42	15.6
K-15-crown-5_2_ (Na)–axial	–177.7	6.20	+0.27	–0.43	18.3
Na-15-crown-5_2_ (K)	–132.9	6.38	+0.28	–0.42	17.0
K-15-crown-5_2_ (K)	–166.7	7.08	+0.28	–0.43	17.9
Superalkali M-HMHC Models					
Na-HMHC	–62.7		+0.31		0
K-HMHC	–90.9		+0.26		0
Superalkali–Alkalide Pair M-HMHC (M) Models					
Na-HMHC (Na)	–152.0	3.61	+0.22	–0.23	9.3
K-HMHC (Na)–chair^c^^,^^d^	–177.0	4.11	+0.24	–0.29	10.6
K-HMHC (Na)–boat^c^^,^^d^	–183.5 (−150.6)	4.00 (4.50)	+0.21 (+0.27)	–0.29 (−0.33)	10.5 (12.6)
Na-HMHC (K)	–130.8	4.36	+0.25	–0.21	9.8
K-HMHC (K)	–159.0	4.73	+0.25	–0.24	9.9

a M is the symbol for the alkali metal, to represent either Na or K. Each HMHC system given was modeled with the chair conformation, except for the “K-HMHC (Na)–boat” where the most stable and least stable positions of the sodide are given outside and within the parentheses, respectively. Each 15-crown-5_2_ system was modeled as the equatorial pair unless labeled differently. M-HMHC and M-15-crown-5_2_ are referred to as superalkalis.

b The binding energies (BE in kJ/mol), calculated in the gas-phase by subtracting the energy of the alkali metal(s) and 15-crown-5/HMHC from the total system energy (see the Supporting Information, eqs S1, S2, S4, and S5).

c The BEs were calculated using the HMHC conformation adopted in the M-HMHC(M) model.

d *E*[K-HMHC (Na)–chair] – *E*[K-HMHC (Na)–boat] = 0.84 kJ/mol.

The flexibility of the macrocycles and approach of the second metal ion allows for several conformationally and topologically different superalkali–alkalide contact ion pairs. In its complexes with alkali cations, HMHC can adopt a conformation with all six nitrogen atoms in one plane, similar to the chair conformation in the crystalline salt of K-HMHC with tetraphenylborate^43^, or a boat conformation as is the case for the crystalline sodide K-HMHC (Na)^44,45^ (Figure 5). For the 15-crown-5 systems, an alkalide can approach either axial or equatorial to the superalkali complex (Figure 6). These two systems are essentially isoenergetic, although the equatorial approach is statistically preferred. The chair and boat forms of K-HMHC (Na) are also isoenergetic, but pure HMHC favors the chair conformation by 5.9 kJ/mol. Calculations exploring the potential energy landscape associated with flipping the HMHC between the chair and boat conformations, both for pure HMHC and within the superalkali–alkalide complex, concluded that the chair form is the dominant species in solution (Supporting Information, section S16). Therefore, we focus upon the chair HMHC in this discussion.

Turning to the NMR parameters, the computed shielding constant for the sodide in K-HMHC (Na) is 40–46 ppm larger than for the sodium cation in Na-HMHC (K) or Na-HMHC (Na). The Hirshfeld charges capture the loss of electron density from the encapsulated metal atom and an increase in electron density on the surrounding metal for all ion paired systems, as expected for the alkalide state being maintained even in a close ion pair. Both K-HMHC (Na) and K-15-crown-5_2_ (Na) possess significant dipole moments, in line with the rationale for the experimentally observed increase in molar conductivity at higher alkalide concentrations (Figure 3). This further rules out weakly dipolar superalkali dimers (Supporting Information Table SI-4).

The chair HMHC superalkali *C*_*i*_ symmetry SOMO extends from the metal symmetrically surrounding the superalkali above and below, in contrast to the 15-crown-5_2_ superalkali *C*_1_ symmetry SOMO that persists about the ether rings but does not have much character near the alkali metal (*cf*. Figures 5 and 6). The HOMOs of the superalkali–alkalide complexes are concentrated on the sodide component, and they have a large spatial extent past the alkalide portion of the ion pair, in agreement with our neutron scattering results and with the crystallographically derived radii of alkalides in the solid state. These HOMOs are formed from a bonding in-phase interaction between the SOMO of the sodium atom with the SOMO of the potassium superalkali, which itself contains character from the LUMOs of the complexant, as well as 4s and 5s K orbitals. The bond orders of 0.65 and 0.57 computed between K and Na highlight the partial covalency of the interaction in K-HMHC (Na) and K-15-crown-5_2_ (Na), respectively. Additional stability in these superalkali and superalkali–alkalide complexes, alongside coordination and M^δ+^M^δ-^ pairing, originates from intramolecular H⇜⇝H interactions first introduced in ref (5). The H⇜⇝H interaction is mediated through orbital overlap between neighboring hydrogen atoms, as seen in the superalkali SOMOs and superalkali–alkalide HOMOs (Figures 5, 6, and Supporting Information Figures SI-9–SI-14, SI-17). This H⇜⇝H bonding arises from the partial population of the LUMO of the organic species when alkali metals are introduced to the system (Supporting Information Figures SI-7 and SI-8) and is reminiscent of Rydberg bonding proposed by Simons.^46,47^ One consequence of this H⇜⇝H interaction is that the distance between pairs of hydrogen atoms is ∼0.1 Å shorter in the superalkali systems as compared to pure HMHC and 15-crown-5.

A fragment orbital analysis was performed using the (spin-restricted) distorted K-HMHC/K-15-crown-5_2_ superalkalis (as found in the optimized superalkali–alkalide species) and the (spin-restricted) Na atoms as the fragments.^21^ This revealed the composition of the molecular orbitals of the complex in terms of the occupied and unoccupied orbitals of the fragments. As shown in Table SI-12, the Na 3s SOMO is the dominant contribution to the all-important HOMO of the chair K-HMHC (Na) and equatorial K-15-crown-5_2_ (Na) complexes (51.8% and 67.9%, respectively) as expected. The SOMO of the superalkali provides the second largest contribution (34.2% and 20.0%), but diffuse unoccupied orbitals on the superalkali are also an important component, suggesting this may be another manifestation of Rydberg bonding (Supporting Information, section S19).^46,47^

### Variable Temperature NMR Spectroscopy

**Figure 7:**
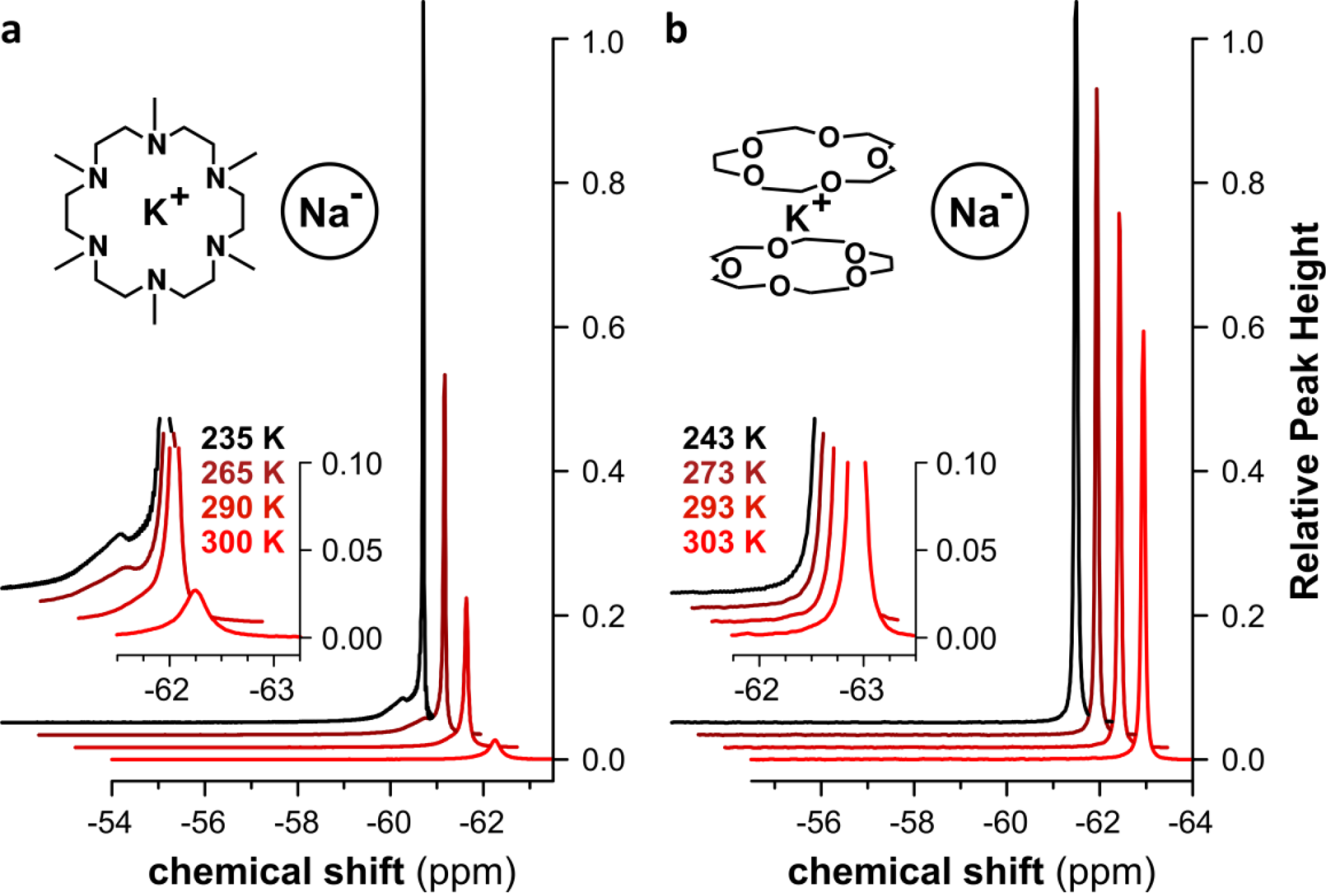
Temperature dependent ^23^Na NMR spectroscopy of sodide solutions in THF. (a) Upfield range of ^23^Na spectra showing the temperature dependent change in shape and total signal intensity of the alkalide signal of a solution of 0.3 M NaK in HMHC/THF in the chemical shift range between (−61) – (−65) ppm. (b) Upfield range of ^23^Na spectra showing the temperature dependent change in shape and total signal intensity of the alkalide signal of a solution of 0.4 M NaK in 15-crown-5/THF in the chemical shift range between (−61) – (−65) ppm. Spectra are referenced to 1 M aqueous NaCl solution at room temperature according to IUPAC recommendations. Peak heights in both sets are normalized to their respective lowest temperatures. Insets show zooms of the signal onsets.

^23^Na and ^39^K NMR spectra under cryogenic conditions exhibit the expected narrow, shielded signals in concentrated solutions of macrocycle/NaK and macrocycle/K in THF, respectively (Figure 7 and Supporting Information Figure SI-1). The dynamics of the alkalide species and their interactions with their local environment or with other components in solution are evidenced through comparison of ^23^Na spectra at different temperatures over an accessible range of more than 60 K (Figure 7). Increasing the temperature results in a rapid and reversible decrease in the total integrated area of signals in the chemical shift range of (−61) – (−63) ppm for both macrocycle systems.

Considering the lack of any additional signals in the chemical shift range of (+100) – (−200) ppm, we attribute the unusual behavior of alkalide species in NMR to an exchange process between an NMR-visible and an NMR-invisible species, each involving the alkali metal nucleus of interest. The line width of the alkalide signal increases only slightly from 2.9 to 9.0 Hz from 235 to 290 K despite the drastic loss in signal intensity. This suggests a thermally activated transformation of the NMR-visible species in the slow exchange limit.^48^ Very minor changes in the chemical shift of the signals cannot be conclusively demonstrated here due to the sensitive temperature dependence of the reference signal.

If the relaxation of the quadrupolar alkalide nucleus was purely due to the approach of the superalkali countercation, it must be induced by a sufficiently large electric field gradient (EFG) at the alkalide nucleus. However, our computed EFG, *V*_*zz*_, of the isolated sodide does not differ from that in the ion pair and is essentially 0 relative to the computed *V*_*zz*_ for Na and K in the superalkali (Supporting Information Tables SI-5 and SI-7). Recent results from *ab initio* molecular dynamics showed that the sodide anion may appear in NMR experiments as if it were an unperturbed, spherical ion, despite the polarizable 3s orbital being strongly affected by the surrounding species in solution.^16^ We note that the solvent modeled in ref (16) was methylamine, a solvent of higher dielectric than the THF solutions used in this study and which would be expected to perturb the alkalide species to an even greater extent. Hence, we conclude that the NMR-visible alkalide species is subject to a fast and reversible exchange process with an NMR invisible species, which is particularly short-lived. This may be a paramagnetic species or a charge-transfer state but is not due to the association and dissociation of the superalkali–alkalide complex.

Another interesting and exclusive feature of the HMHC sodide system observed upon close inspection of the ^23^Na NMR is the existence of a small shoulder in the major signal on the low-field side (Figure 7a). This shoulder becomes more pronounced as the signals drift apart with decreasing temperature. The small difference in chemical shift between the primary and secondary signals around the −60 ppm chemical shift range suggests the involvement of a coordinative or, more likely, a conformational difference between both NMR-visible species without a loss of its integrity.

Mindful of our above discussion concerning the effect of a perturbation of the alkalide on its NMR signature, we suggest that this is due to the conformational flexibility of the superalkali–alkalide complex of HMHC that is absent in the 15-crown-5 case, bearing in mind that both chairlike and boatlike conformations for the HMHC macrocycle exist in crystalline systems. Clearly, further work remains as to identifying the precise nature, and mechanisms, of these intriguing exchange processes.

## Conclusion

Alkalides have a unique place in the history and chemistry of the s-block elements.^1^ Since their discovery by J. L. Dye and colleagues, the characterization of the alkalide species in condensed matter systems has led to the fascinating discussion of their diffuse, yet localized, electronic states in weakly polar solvents and indeed to the application of alkalides as highly reductive species across chemistry.^25,49−51^ The existence of ion paired species has been mooted since the very earliest discussions of alkalides but has never been experimentally demonstrated conclusively.

Herein, we have provided experimental evidence of the observation and effect of ion pairing of alkalides in solution, both from examination of their macroscopic conductivity and dielectric properties to the local disruption of solvent scattering density witnessed in coherent neutron scattering. To suggest what form these ion pairs may take in solution, we have carried out DFT calculations on a number of possible superalkali–alkalide complexes in addition to superalkalis and superalkali dimers. In agreement with recent *ab initio* results,^16^ our models implicate that the superalkali–alkalide complex, which we suggest is the dominant species in solution, may be indistinguishable in NMR from an isolated solvated alkalide and so we have revisited the classical interpretation of such data in the literature of alkalides. We attach great importance to the temperature dependence of the conformationally dynamic HMHC system, indicating that there is much more to be understood as to the kinetics of alkalides in solution: The reversible perturbation and possible disintegration of the NMR-visible species (that may well be the contact ion pair) highlights the significance of the interaction of alkalides with their complex countercations.

Our studies paint a picture of an alkalide species being far beyond a “gaslike” ion in solution, but instead one that could be “chemically” controlled and developed by considering superalkali–alkalide interactions of the sort delineated here. We believe that the interactions of alkalides in solution is merely the most recent in a long line of surprising and unique aspects of s-block chemistry^1^ and certainly one that has the potential to affect how these systems are extended and applied in the future.

## Supporting Information Available

Experimental protocols and full characterization, NMR data, description of ionic conductivity methodology, dielectric spectroscopy methodology and data, computational details and data, isosurfaces, and structure coordinates (PDF)

## Abbreviations

15C5: 15-crown-5
DFT: density functional theory
EFG: electric field gradient
HMHC: 1,4,7,10,13,16-hexamethyl-1,4,7,10,13,16-hexaazacyclooctadecane
HOMO: highest occupied molecular orbital
LUMO: lowest unoccupied molecular orbital
NMR: nuclear magnetic resonance
SANS: small angle neutron scattering
SOMO: singly occupied molecular orbital
THF: tetrahydrofuran
