# Supplementary material for: Superalkali–Alkalide Interactions and Ion Pairing in Low-Polarity Solvents
Source: J Am Chem Soc. 2021 Mar 4;143(10):3934–43. doi: 10.1021/jacs.1c00115 (PMC8028040; doi:10.1021/jacs.1c00115)
Supplement: Supplementary file 1 — ja1c00115_si_001.pdf [file ja1c00115_si_001.pdf]

Supporting Information

Superalkali–Alkalide Interactions and Ion Pairing in Low-Polarity Solvents

R. Riedel[1]*, A.G. Seel[2,3]*, D. Malko[1], D.P. Miller[4], B.T. Sperling[4], H. Choi[5], T.F. Headen[6], E. Zurek[7], A. Porch[5], A. Kucernak[1], N.C. Pyper[8], P.P. Edwards[3]*, A.G.M. Barrett[1]*

[1]Department of Chemistry, Imperial College London, Molecular Sciences Research Hub, White City Campus, Wood Lane, London W12 0BZ, U.K.
[2]Department of Physics and Astronomy, University College London, Gower Street, London WC1E 6BT, U.K.
[3]Inorganic Chemistry Laboratories, University of Oxford, Park Royal Road, Oxford OX1 3QR, U.K.
[4]Department of Chemistry, Hofstra University, 106 Berliner Hall, Hempstead, New York 11549, United States
[5]School of Engineering, Cardiff University, Cardiff CF24 3AA, U.K.
[6]ISIS Neutron and Muon Source, Science and Technology Facilities Council, Rutherford Appleton Laboratory, Harwell Campus, Didcot OX11 0QX, U.K.
[7]Department of Chemistry, State University of New York at Buffalo, 777 Natural Sciences Complex, Buffalo, New York 14260-3000, United States
[8]University Chemical Laboratory, Lensfield Road, Cambridge CB2 1EW, U.K.
*e-mail: rene.riedel13@alumni.imperial.ac.uk; a.seel@ucl.ac.uk; agm.barrett@imperial.ac.uk; peter.edwards@chem.ox.ac.uk

1 CONTENT

1 Content S2

2 Synthesis of aza-crown hexamethyl-hexacyclen (HMHC, **1**) S3

3 NMR Spectroscopy S9

4 Conductivity and Viscosity measurements S10

5 Dielectric Spectroscopy S13

6 Small Angle Neutron Scattering and Density measurements S16

7 Computational Details S20

8 Structure, HOMO, and LUMO of the HMHC Molecule S22

9 Structure, HOMO, and LUMO of the 15-crown-5 Molecule S23

10 Alkali Metal HMHC - Structure, SOMO, and HOMO S24

11 Alkali Metal 15-crown-5 - Structure, SOMO, and HOMO S27

12 Superalkali Dimer Structures and HOMOs S29

13 Computational Analysis of the Alkali Metal HMHC Models S30

14 Discussion on the HMHC Superalkali Dimer Models S32

15 Computational Analysis of the Alkali Metal 15-Crown-5 Models S33

16 Examination of HMHC Intermediates for the Chair/Boat Conformations S35

17 Computational Study on the Interaction of THF with Na and K S37

18 Superalkali-Alkalide HOMOs with Small Isovalues S41

19 Fragment Orbital Analysis S42

20 Coordinates (XYZ) S43

2 SYNTHESIS OF AZA-CROWN HEXAMETHYL-HEXACYCLEN (HMHC, 1)

Instrumental procedures

Melting points were determined using a Reichert-Thermovar hot-stage apparatus and are uncorrected. IR spectra were recorded as thin films with the absorption bands reported in wave number (cm^{-1}) using a Mattson 5000 FTIR apparatus with automatic background subtraction. ^{1}H NMR spectra were recorded at 400 MHz and ^{13}C NMR spectra at 100 MHz on Bruker DRX-400 spectrometers respectively. Chemical shifts are reported as δ-values in ppm relative to the $CDCl_3$ peaks (δH 7.26, δC 77.0). Coupling constants (*J*) are recorded in Hertz (Hz) and quoted to the nearest 0.5 Hz. Low and high resolution mass spectra (ESI) were recorded by Imperial College Mass Spectrometry service using a Micromass Platform II and a Micromass AutoSpec-Q spectrometer.

General reaction procedures

All reactions were carried out with a magnetic stirring and, if air or moisture sensitive, in a flame-dried or oven-dried glassware under nitrogen or argon. Syringes, used to transfer reagents and solvents, were purged with nitrogen or argon prior to use. Reaction temperatures other than room temperature were recorded as the bath temperature unless otherwise stated. Prolonged periods of vessel cooling were attained by the use of CryoCool apparatus. All solvents and reagents were used as commercially supplied, unless otherwise stated. Et_2O, THF and CH_2Cl_2 were redistilled from Na-Ph_2CO, Na and CaH_2 selectively. Thin layer chromatography was performed on pre-coated aluminum backed silica gel F254 glass plates. The chromatogram was visualized under UV light and/or by staining using potassium permanganate solution or aqueous acidic vanillin followed by gentle heating with a heat gun. Flash column chromatography was performed using silica gel, particle size 40-63 μm (eluents are given in parenthesis). The diameter of the columns and the amount of silica gel were calculated according to the recommendations of W. C. Still[1].

[1] Still, W. C.; Kahn, M.; Mitra, A. Rapid chromatographic technique for preparative separations with moderate resolution. *J. Org. Chem.* **1978**, *43*, 2923–2925.

***N, N', N'', N'''*-Tetra-(4-methylphenylsulfonyl)-1,4,7,10-tetraazadecane (2)**

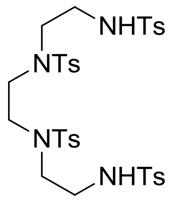

4-Toluenesulfonyl chloride (45.8 g, 0.24 mol, 4 equiv.) in Et_2O (50 mL) and water (50 mL) was cooled to 5 °C and triethylenetetramine (8.80 g, 60 mmol, 1 equiv.) in NaOH solution (5 M; 50 mL) was added under vigorous stirring. After 1 h, the ice bath was removed, and the mixture was stirred for a further 4 h at room temperature. The mixture was acidified with 2 M HCl, the white tacky precipitate was filtered off and washed with water, methanol and Et_2O. The crude material was purified via boiling in large amount of methanol, filtration, washing with ether and drying under vacuum to obtain the title compound **2** (34.3 g, 75%) as white crystals.
mp 210 °C (Lit.[2] 215-217 °C).
IR (neat, cm^{-1}) 3280, 2930, 2846, 1571, 1460, 1309, 1117.
^{1}H NMR (400 MHz, $CDCl_3$): δ 2.42 (s, 6H), 2.44 (s, 6H), 3.16 (m, 4H), 3.22 (m, 4H), 3.42 (s, 4H), 5.46 (t, ^3J = 6.0 Hz, 2H), 7.31 (d, ^3J = 8.0 Hz, 4H), 7.34 (d, ^3J = 8.1 Hz, 4H), 7.74 (dd, ^3J = 8.2 Hz, 8H), 8.08 (s, 4H).
^{13}C NMR (100 MHz, $CDCl_3$): δ 21.5, 43.7, 50.9, 127.1, 127.4, 128.4, 129.8, 129.9, 131.0, 133.6, 134.6.
HRMS (ESI): calc. for $C_{34}H_{42}N_4O_8NaS_4$: $(M + Na)^+$, 785.1783; found: $(M + Na)^+$, 785.1822.

[2] Benini, A.; Burguete, M. I.; Garcia-Espana, E.; Luis, S. V.; Miravet, J. F.; Soriano, C. An efficient synthesis of polyaza[n]paracyclophanes. *J. Org. Chem.* **1993**, *58*, 4749–4753.

_N,N'_-Di-(2-(4-methylphenylsulfonyl)ethyloxy)_N,N'_-di-(4-methylphenylsulfonyl)-1,2-ethanediamine (3)

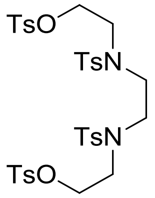

4-Toluenesulfonyl chloride (76.3 g, 0.40 mol, 4 equiv.) in CH_2Cl_2 (150 mL) was added dropwise at 10 °C with vigorous stirring to a mixture of *N,N'*-di-(2-hydroxyethyl)-1,2-diaminoethane (14.8 g, 0.10 mol, 1 equiv.), triethylbenzylammonium chloride (1.00 g), aqueous NaOH (30%; 100 mL) and CH_2Cl_2 (150 mL). After 7 h the mixture was poured into water (500 mL). The organic layer was separated, washed with water (2 x 300 mL) and dried over Na_2SO_4. The solvent was evaporated under reduced pressure and the residue was recrystallized from ethanol to obtain the title compound **3** (50.4 g, 66%) as thin white needles.
mp 146 °C (Lit.[3] 147-149 °C).
IR (neat, cm^{-1}) 2959, 1597, 1326, 1150, 1098, 813.
^{1}H NMR (400 MHz, $CDCl_3$): δ 2.44 (s, 12H), 3.30 (s, 4H), 3.36 (t, ^3J = 5.4 Hz, 4H), 4.14 (t, ^3J = 5.3 Hz, 4H), 7.34 (d, ^3J = 8.1 Hz, 8H), 7.71 (d, ^3J = 8.2 Hz, 4H), 7.77 (d, ^3J = 8.3 Hz, 4H).
^{13}C NMR (100 MHz, $CDCl_3$): δ 21.6, 21.7, 49.5, 49.9, 69.0, 77.2, 127.4, 128.0, 130.0, 132.4, 135.0, 144.0, 145.2.
HRMS (ESI): calc. for $C_{34}H_{41}N_2O_{10}S_4$: $(M + H)^+$, 765.1644; found: $(M + H)^+$, 765.1702.

[3] Luk'yanenko, N. G.; Basok, S. S.; Filonova, L. K.; Kulikov, N. V.; Pastushok, V. N. Synthesis of macrocyclic polyamines in a biphasicsystem. *Chemistry of Heterocyclic Compounds* **1990**, *26*, 346–349.

1,4,7,10,13,16-Hex-(4-methylphenylsulfonyl)-1,4,7,10,13,16-hexa-aza-cyclo-octadecane (4)

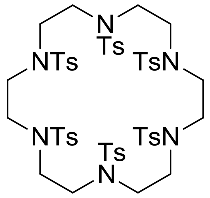

Sodium hydride (60% suspension in oil; 2.60 g, 65 mmol, 3.3 equiv.) was added with stirring to *N*,*N'*,*N''*,*N'''*-Tetra-(4-methylphenylsulfonyl)-1,4,7,10-tetraazadecane **2** (15.3 g, 20 mmol, 1.0 equiv.) in dry DMF (220 mL) under a nitrogen atmosphere to form the disodium salt. After most effervescence subsided, the solution was heated to 70 °C, and, on cessation of hydrogen evolution, the yellow solution was filtered off from excess NaH under a dry nitrogen atmosphere. The filtrate was stirred at 105 °C, when a solution of *N'*-Di-(2-(4-methylphenylsulfonyl)ethyloxy)*N,N'*-di-(4-methylphenylsulfonyl)-1,2-ethanediamine **3** (15.3 g, 20 mmol) in dry DMF (200 mL) was added over 2 h. The orange solution was stirred at 105 °C for a further 2 h. After reduction of volume (100 mL) to induce crystallization, water (300 mL) was added to produce a flocculent white precipitate. This was filtered off, sequentially washed with water, ethanol, and Et_2O, and dried under vacuum to obtain the title compound **4** (21.6 g, 91%) as a white powder.

IR (neat, cm^{-1}) 2925, 1598, 1457, 1334, 1151, 1089, 976, 811, 722.

^{1}H NMR (400 MHz, $CDCl_3$): δ 2.44 (s, 18H), 3.23 (s, 24H), 7.32 (d, ^3J = 8.1 Hz, 12H), 7.69 (d, ^3J = 8.2 Hz, 12H).

^{13}C NMR (100 MHz, $CDCl_3$): δ 21.6, 49.6, 127.5, 130.0, 135.0, 144.0.

HRMS (ESI): calc. for $C_{54}H_{67}N_6O_{12}S_6$: $(M + H)^+$, 1183.3141; found: $(M + H)^+$, 1183.3142.

1,4,7,10,13,16-hexa-aza-cyclo-octadecane (5)

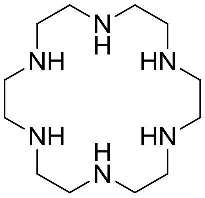

1,4,7,10,13,16-Hexa-(4-methylphenylsulfonyl)-1,4,7,10,13,16-hexa-aza-cyclo-octadecane **4** (8.30 g, 7.0 mmol, 1.0 equiv.) was dissolved in concentrated sulfuric acid (96%, 30 mL) and maintained under stirring at 105 °C for 48 h and the resultant dark-brown solution was subsequently cooled in an ice bath. Ethanol (100 mL) and Et_2O were added slowly with stirring. A brownish precipitate was formed, presumably the sulfate of the protonated ligand, which was filtered off and dissolved in ice-cooled aqueous NaOH solution (20%; 20 mL). The free amine was extracted with chloroform (10 x 20 mL) and the combined organic layers were dried over Na_2SO_4. The solvent was evaporated under reduced pressure and the residue dried under vacuum to obtain the title compound **5** (1.10 g, 60%) as a white powder.

IR (neat, cm^{-1}) 3274, 3034, 2922, 1599, 1460, 1323, 1153, 1088, 979, 810.

^{1}H NMR (400 MHz, $CDCl_3$): δ 1.65 (bs, 6H), 2.72 (s, 24H).

^{13}C NMR (100 MHz, $CDCl_3$): δ 49.3.

HRMS (ESI): calc. for $C_{12}H_{31}N_6$: $(M + H)^+$, 259.2610; found: $(M + H)^+$, 259.2615.

1,4,7,10,13,16-Hexamethyl-1,4,7,10,13,16-hexa-aza-cyclo-octadecane (1)

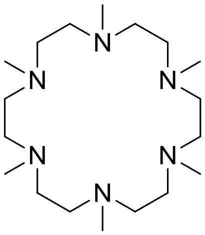

Paraformaldehyde (2.49 g, fivefold excess) was added in one portion with stirring to 1,4,7,10,13,16-hexa-aza-cyclo-octadecane **5** (1.00 g, 3.9 mmol) in formic acid (90%, 10 mL) at room temperature. The suspension was heated at reflux under nitrogen atmosphere. After 72 h, the bulk of the excess of formic acid was removed by distillation and the residue was diluted with water, basified with aqueous KOH and extracted with dichloromethane (3 x). The organic extracts were combined, dried, filtered, and evaporated under reduced pressure to give a brown oily residue. Kugelrohr distillation under high vacuum (oven temperature 130 °C, 1 x 10^{-5} mbar) gave the hexa-amine **1** (1.06 g, 80% yield) as a colorless oil which darkened on prolonged exposure to the atmosphere.

IR (neat, cm^{-1}) 2945, 2773, 1672, 1454, 1355, 1298,c1113, 1035, 938, 772.

^{1}H NMR (400 MHz, $CDCl_3$): δ 2.26 (s, 18H), 2.51 (s, 24H).

^{13}C NMR (100 MHz, $CDCl_3$): δ 43.8, 55.2.

HRMS (ESI): calc. for $C_{18}H_{43}N_6$: $(M + H)^+$, 343.3549; found: $(M + H)^+$, 343.3549.

3 NMR SPECTROSCOPY

a

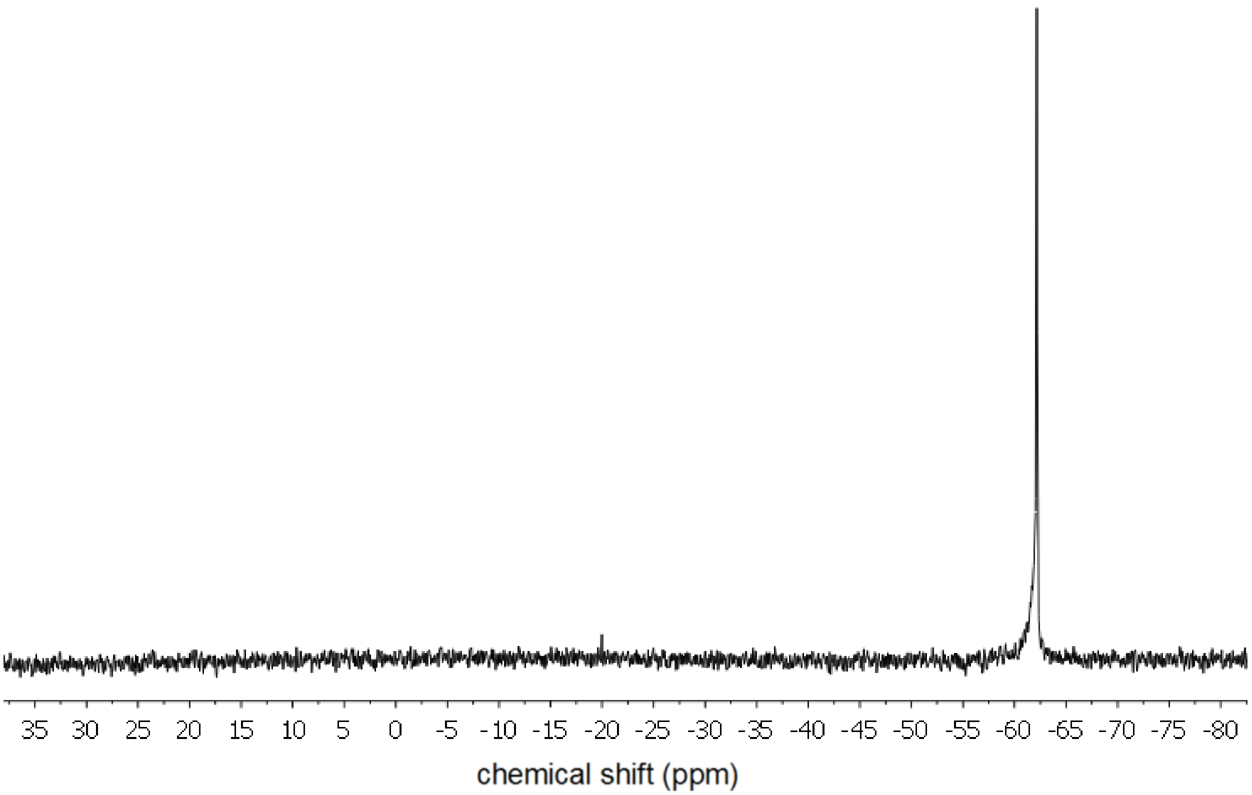

b

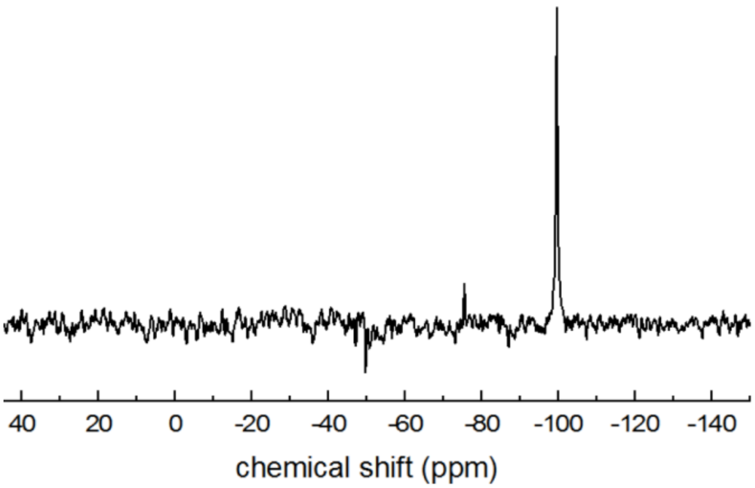

Figure SI-1 | Alkali metal NMR spectra of alkalide solutions in THF. **a** ^{23}Na spectrum of HMHC(K^+)Na^- in THF at 245 K. **b** ^{39}K spectrum of HMHC(K^+)K^- in THF at 245 K.

4 CONDUCTIVITY AND VISCOSITY MEASUREMENTS

Concentration-dependent conductivity data

Impedance measurements were carried out using a potentiostat Gamry Reference 600 and using platinized platinum electrodes (cell constant 0.98 cm). Molar conductivity values were calculated from the specific resistance in the high frequency range (10-100 kHz).
The cell constant of the platinum electrode was determined before and after alkalide experiments which gave identical values. Preliminary tests showed that platinum did neither dissolve nor even show any noticeable signs of their degradation upon prolonged exposure to alkalide solutions in THF.
The temperature control was provided by a Huber TC45E-F immersion cooler.
Karl-Fischer titrations were performed with an automatic titration system (Crison model KF431) equipped with a digital burette (Crison model 738).
The obtained temperature-dependent viscosity trends for each concentration were fitted by an exponential function (Arrhenius-type) and extrapolated to lower temperatures (Figure SI-2, Table SI-1). The viscosity values η — calculated for 30 °C (243 K) — were then normalized to the viscosity at the lowest solute concentration and the obtained fractions η_{norm} were used to calculate the Walden product $\Lambda_m\eta_{norm}$.

Conductivity data were discussed in the form of the Walden product to account for any changes in viscosity while being aware of its limitation[4].
Despite extensive efforts within the employed experimental procedure, traces of remaining water in the solvent in the concentration range of 5 -10 ppm were inevitable[5]. Dissolution of an alkali metals and formation of highly reducing alkalide species in the medium would result in the conversion of any water traces to alkali hydroxides which would contribute to the measured alkali metal concentration without contributing to the measured conductivity due to extensive ion pairing. Indeed, the intentional use of slightly wet THF (20 – 30 ppm) delayed the development of the characteristic blue color of the mixture after metal addition. In order to correct the original molar conductivity data, all collected data points were corrected for the background concentrations of up to10 ppm as determined by Karl-Fischer titration of the solvent immediately before each conductivity experiment. The resulting data follow the aforementioned general trend but suggest a significantly higher limiting molar conductivity Λ_l and a more pronounced decrease in molar conductivity with increasing metal concentration.
To ensure that the conductivity measured can be indeed ascribed to the alkalide species, the effect of decomposition products on the conductivity of an alkalide solution was probed. For that purpose, a saturated solution of NaK in 0.4 M 15-crown-5 in THF was kept at room temperature under inert atmosphere until complete discoloration. Addition of the obtained solution to a more dilute alkalide solution in a way, that the total metal concentration of the mixture was at least twice as large as the metal concentration of the original alkalide solution, did not show any notable effect on the conductivity of the mixture.

---

[4] Nakahara, M.; Ibuki, K. Is the Walden product useful? *J. Phys. Chem.* **1986**, *90*, 3026–3030.
[5] Williams, D. B.; Lawton, M. Drying of organic solvents: quantitative evaluation of the efficiency of several desiccants. *J. Org. Chem.* **2010**, *75*, 8351–8354.

Viscosity measurements

a

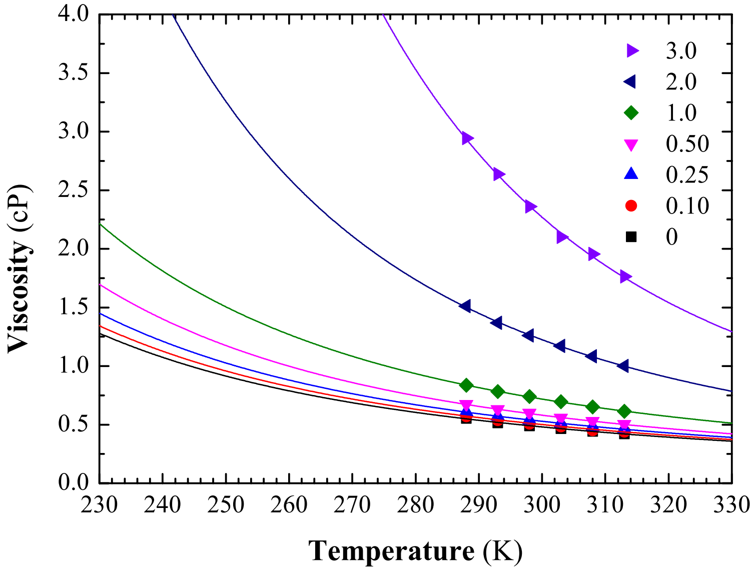

b

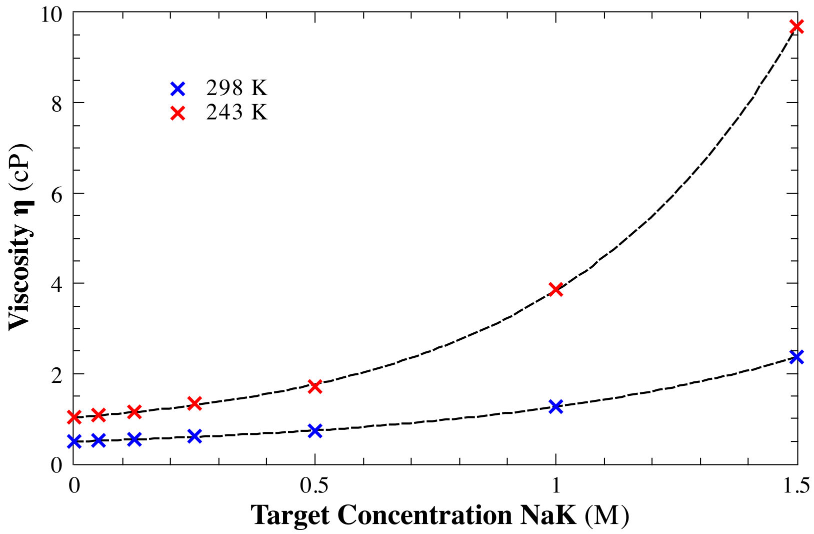

Figure SI-2 | Temperature and concentration dependence of shear viscosity η of metal-free solutions of 15-crown-5 in THF. **a** Temperature dependence of shear viscosity η of metal-free solutions of 15-crown-5 in THF. Measured shear viscosity values (data points) at each concentration of 15-crown-5 (given in M) were extrapolated (single exponential fit function; solid lines) to determine shear viscosity values at 243 K. **b** Concentration dependence of shear viscosity η of metal-free solutions of 15-crown-5 in THF at 298 K (blue, measured) and 243 K (red, from extrapolation). Single exponential fit functions (dashed lines) were used to determine viscosity values at any given concentration.

Table SI-1 | Experimentally determined (normal font) and extrapolated (italic) shear viscosity values of metal-free solutions of 15-crown-5 (15C5) in THF. Results of exponential fit of experimental values using Arrhenius equation are attached, respectively.

**Temp** [K]	**η THF**^{ref} [cP]	**η THF exp.** [cP]	**Viscosity η of 15-crown-5 in THF** [M]						**η 15C5** [cP]
			**0.10** [cP]	**0.25** [cP]	**0.50** [cP]	**1.0** [cP]	**2.0** [cP]	**3.0** [cP]	
243	0.903	*1.022*	*1.072*	*1.151*	*1.327*	*1.711*	*3.860*	*9.692*	*462.7*
253	0.779	*0.874*	*0.915*	*0.979*	*1.118*	*1.428*	*3.040*	*7.166*	*242.9*
263	0.679	*0.756*	*0.791*	*0.843*	*0.954*	*1.209*	*2.438*	*5.421*	*133.9*
273	0.598	*0.661*	*0.690*	*0.734*	*0.823*	*1.036*	*1.987*	*4.186*	*77.14*
283	0.532	*0.583*	*0.609*	*0.645*	*0.718*	*0.897*	*1.643*	*3.292*	*46.20*
288	0.503	0.553	0.574	0.608	0.674	0.837	1.510	2.943	36.54
293	0.477	0.516	0.542	0.572	0.629	0.782	1.367	2.637	28.34
298	0.452	0.489	0.511	0.539	0.599	0.735	1.260	2.361	22.53
303	0.430	0.465	0.487	0.513	0.557	0.690	1.170	2.100	18.29
308	0.410	0.443	0.455	0.482	0.529	0.652	1.082	1.955	14.94
313	0.391	0.422	0.442	0.463	0.504	0.613	1.000	1.763	12.42
η_0 [10^{-4} cP]		192	196	191	171	177	91.7	46.6	0.385
**E** [kJ/mol]		8.024	8.084	8.275	8.789	9.230	12.210	15.440	32.930
R^2		0.997	0.995	0.998	0.997	0.998	0.998	0.998	0.998

5 DIELECTRIC SPECTROSCOPY

We use a resonant method for measuring the complex permittivity, based on the coaxial probe described previously[6]. Whilst this necessarily operates at a discrete set of resonant frequencies, it has the advantage over non-resonant approaches (such as the open-ended coaxial probe[7]) of enhanced sensitivity, especially for dielectric loss, which is of prime here importance since it links to the electrical conductivity of the liquids. Making the resonant coaxial line long results in a closer harmonic spectrum, in the usual way, so many modes can be assessed over the 0.35 to 6 GHz bandwidth of the experiments presented here.

To avoid non-TEM modes and make sure broadband operation the cross-sectional geometry should be much smaller than the wavelength**.** PTFE-filled RG401 50Ω coaxial cable is chosen in the design for several reasons, but mainly for its wide availability, low cost and rigidity for this application. Both ends of the coaxial cable is cut and polished to form half-wavelength resonator, where one end will be in touch with the sample under test while the opposite end will have input and output coupling ports. The relationship between the length and resonant frequencies can be defined as follows,

$$f_N = \frac{N}{2l} \cdot \frac{c}{\sqrt{\varepsilon_r}}, \tag{1}$$

where f_N is the harmonic resonant frequency, l is the length of the coaxial resonator where $l = \lambda/2$, N is the harmonic number defined to be the number of half wavelengths along the length, and c is the speed of light, and ε_r is the relative permittivity of the dielectric material (PTFE). According to (1), a length of 300 mm gives a fundamental resonant frequency of about 350 MHz, with higher order harmonics at 700 MHz, 1050 MHz, …, N×350 MHz. This length allows the investigation of the dielectric properties of the target sample under test over RF (low N) and microwave frequency range (high N).

Capacitive microwave coupling to the electric field (E-field) is made at one end of the cable using SMA connectors and the sample is placed at the other end. An aluminum fixture is used to hold coaxial cable and two coupling ports of SMA connectors. Since the cable is open circuit at each end, the E-field in each resonant mode is maximum at both ends, yielding effective microwave coupling that increases with increasing frequency, and effective E-field coupling to the sample for assessment of its dielectric properties. The distance between the two ports (*Distance*) is chosen to be 13mm so that there is no direct coupling between two ports.

All experimental data is taken using a resonant coaxial probe (300 mm length, 1.63 mm inner conductor, 0.635 mm outer conductor, fundamental resonance at 350 MHz with 14 additional harmonic resonances), flexible microwave coaxial cables, and a microwave vector network analyzer (Keysight Fieldfox N9923A) under computer control (see Figure SI-3). The resonant coaxial probe was calibrated with a polypropylene protection cap. The resistance of the cap

---

6 Rowe, D. J.; Porch, A.; Barrow, D. A.; Allender, C. J. Novel coupling structure for the resonant coaxial probe. *IEEE Transactions on Microwave Theory and Techniques* **2012**, *60*, 1699–1708.

7 Gregory, A. P.; Clarke, R. N. A review of RF and microwave techniques for dielectric measurements on polar liquids. *IEEE Transactions on dielectrics and electrical insulation* **2006**, *13*, 727–743.

towards common solvents and alkali metal solutions in THF was confirmed by exposure tests. No noticeable decomposition of the protection caps was observed by visual inspection after exposure to metal solutions at room temperature for several days.

A bespoke LabView program (National Instruments) is used to record and extract continuous changes in resonant frequency, 3dB bandwidth and peak power at resonance for each mode.

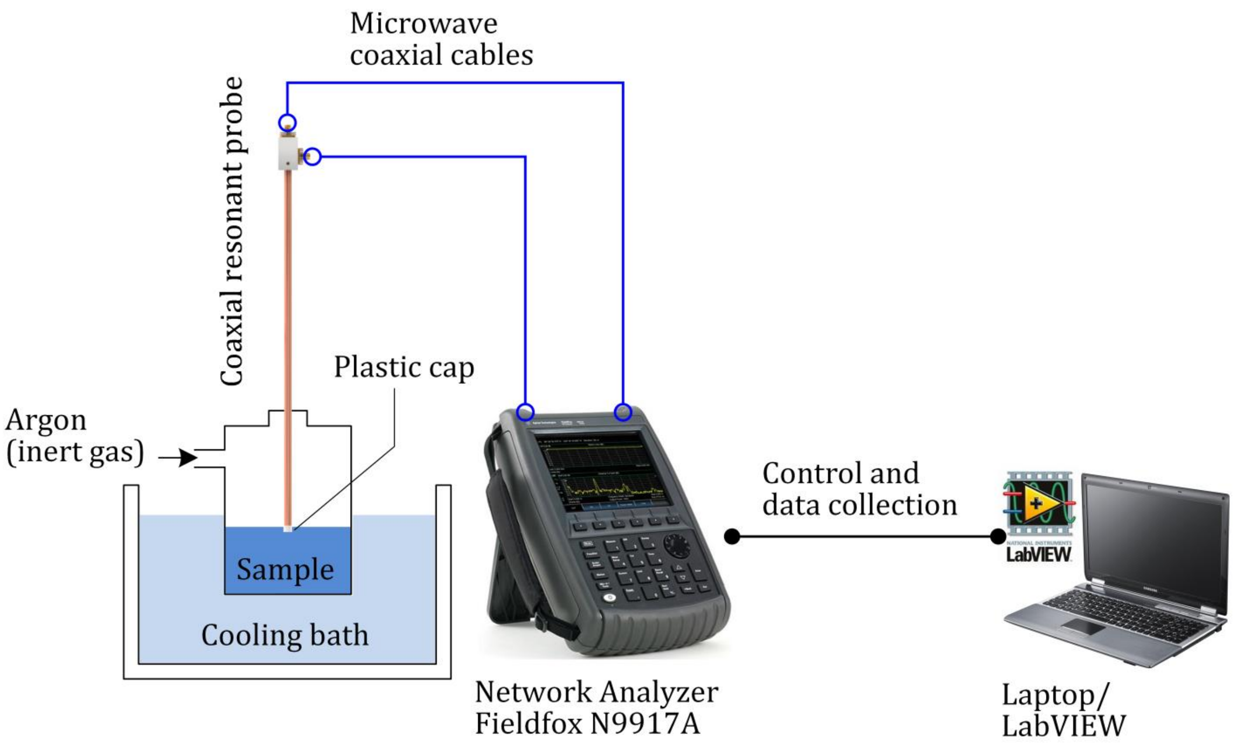

Figure SI-3 | Experimental setup for dielectric spectroscopy measurements.

a

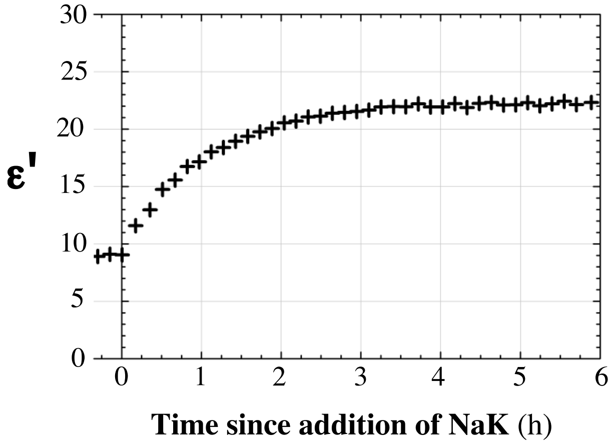

b

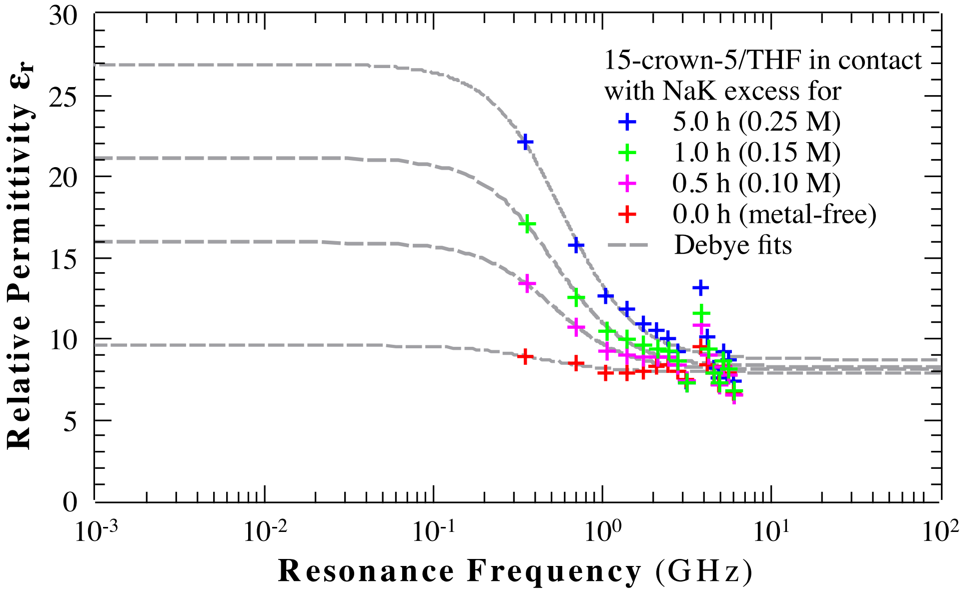

Figure SI-4 | Dielectric spectroscopy of sodide solutions. **a** Development of the static relative permittivity ε' of a solution of NaK in 0.5 M 15-crown-5 in THF over time upon metal dissolution from an excess of NaK at 243 K (permittivity recorded at fundamental resonance frequency 0.35 GHz). **b** Frequency dependence of the relative permittivity of solutions of NaK in 15-crown-5/THF at different concentrations. Debye fit functions (dashed lines) were used as guides to the eye to allow for estimates of the real static relative permittivity of the sodide solutions.

6 SMALL ANGLE NEUTRON SCATTERING AND DENSITY MEASUREMENTS

Fits to the small-angle/low-Q region of the neutron scattering data are presented in Figure SI-5, using a model ellipsoidal form factor incorporating independent polar and equatorial radii, r_p and r_e respectively, scaled by an arbitrary factor. The extracted dimensions of the scattering objects and the difference between their neutron scattering length densities (SLD) and that of the deuterated THF solvent ($\Delta\rho = \rho_{\text{system}} - \rho_{THF}$) are reported in Table SI-2. Before discussing these values, as is discussed in the main text it should be reiterated that the length scales for crown systems are not truly in the low-Q limit (equation 1), being of the same order as a few solvation shells *i.e.* several Angstroms. In terms of scattering, we are therefore looking across dimensions where the average solvent SLD has been disrupted, expected to include any macrocycle/ion-pair contribution as well as their primary solvation shells. The use of a model ellipsoid form factor with constant ρ is therefore somewhat erroneous; the proton possesses a negative scattering length whilst C, N, O, and the metal ions each possess positive scattering lengths. For example, methyl groups (comprising the 'surface' of HMHC) possess a net-negative SLD whereas the ring component of the macrocycle is a weakly positive scatterer. Nevertheless, the existence of a low-Q signal demonstrates that the crown-containing species do indeed act as scattering sources across this Q-range, their being hydrogenous and possessing an average SLD on order of magnitude lower than the solvent.

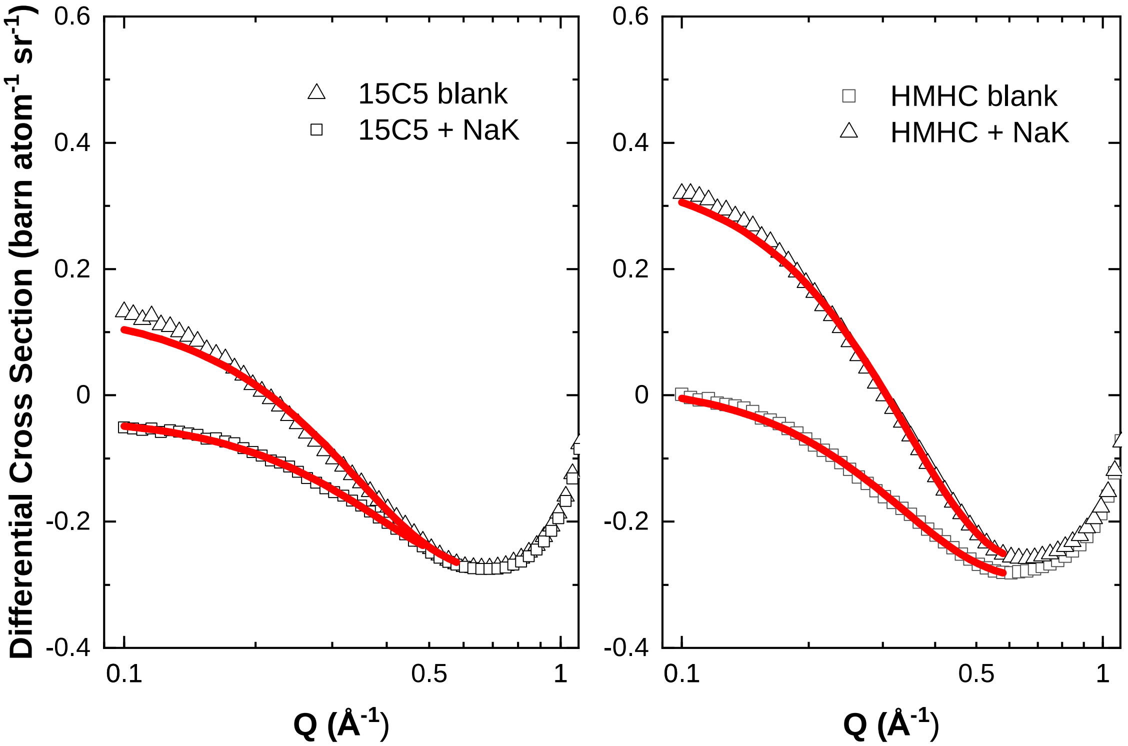

Figure SI-5 | Fitted neutron scattering data to an ellipsoid model in the low-Q region.

The first point to discuss from Figure SI-5 and Table SI-2 is that an ellipsoid model fits the data fairly well, despite it not considering any explicit solvation structure of the crown-containing species. For both the 15-crown-5 (15C5) and HMHC systems we find an increase in the volume of the scattering object going from the blank solution to the NaK containing alkalide systems, and in all cases the $\Delta\rho$ values are negative as expected. The dimensions of the fitted ellipsoids for the blank samples are approximately those of the crown themselves but expanded slightly, presumably due to the both the coarseness of the model and contribution from the first crown-THF solvation layer. The volume increase in our scattering objects upon dissolution of NaK is far too small to be attributed to a conglomeration of the macrocycles, indicating that this expansion is due to ion-paired species. It is worth noting that the fitted values for $\Delta\rho$ differ slightly between the 15C5 and HMHC systems upon metal dissolution. The absolute SLD for the 15C5+NaK system is slightly more positive than for the blank 15C5, whereas for HMHC+NaK the difference to the blank is slightly more negative. These differences are extremely small considering the value for $\rho_{THF} = 6.9\ \times 10^{-6}$ Å^{-2} at the temperature of measurements, but could be due to the larger variation in scattering length magnitude across the HMHC systems than the 15C5. We also note that an ellipsoidal shape is maintained for the 15C5 system upon metal dissolution, in agreement with a side-on superalkali-alkalide approach, whereas for HMHC the alkalide component results in a much more spherical scattering object. This agrees with the facial interaction between alkalide and superalkali components.

Table SI-2| Extracted dimension and scattering length density (SLD) for ellipsoid models in HMHC and 15C5 alkalide solutions in deuterated THF.

**System**	**r_p, r_e (Å)**	**Volume (Å^3)**	**Δρ ($\times 10^6$Å^{-2})**	**Scale Factor**
15C5 blank	$3.14 \pm 0.18, 6.62 \pm 0.07$	576 ± 34	-6.69 ± 0.17	0.102 ± 0.02
15C5 + NaK	$3.45 \pm 0.04, 7.48 \pm 0.04$	809 ± 10	-6.53 ± 0.19	0.125 ± 0.01
HMHC blank	$2.89 \pm 0.03, 7.81 \pm 0.02$	738 ± 14	-6.98 ± 0.20	0.092 ± 0.01
HMHC + NaK	$6.46 \pm 0.02, 6.60 \pm 0.02$	1179 ± 18	-7.23 ± 0.20	0.100 ± 0.03

a

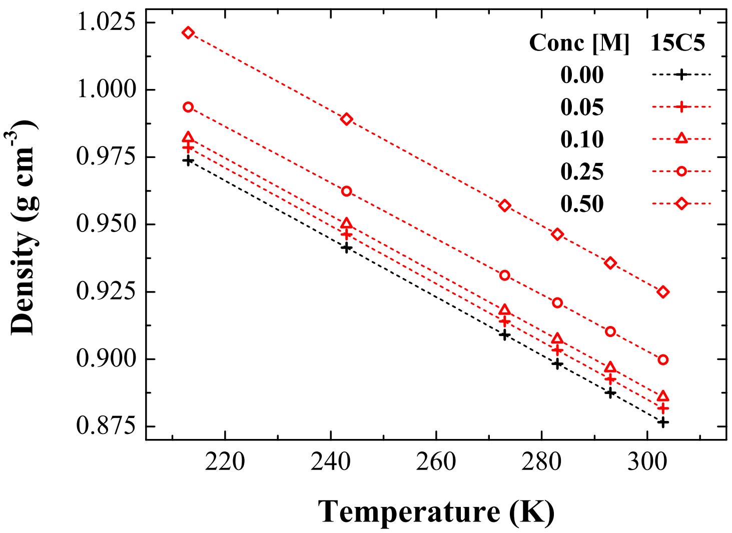

b

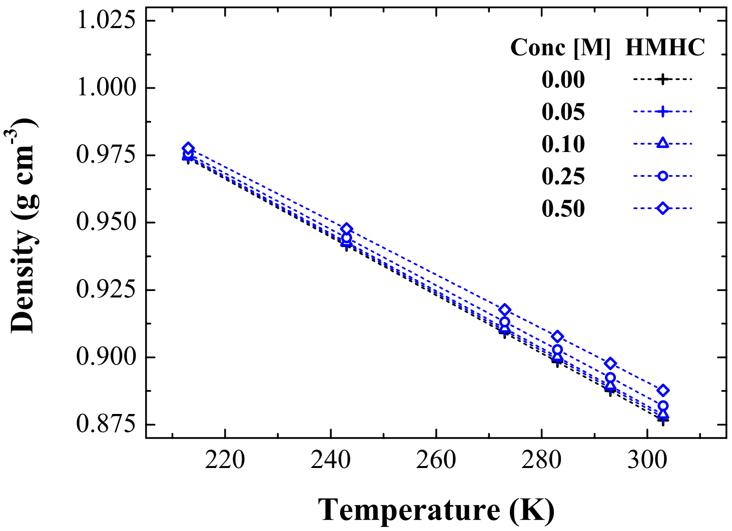

Figure SI-6 | Temperature dependent density measurements of blank solutions of 15-crown-5 in THF. **a** Temperature dependence of the density of solutions of 15-crown-5 in THF at different macrocycle concentrations. **b** Temperature dependence of the density of solutions of HMHC in THF at different macrocycle concentrations.

Table SI-3 | Experimentally determined (normal font) and linearly extrapolated (*italic*) density values of metal-free solutions corresponding to target concentrations of NaK of SANS samples.

**Temp**	**ρ THF**	**Density ρ** [g cm^{-3}] **of THF /** ***15C5***				**Density ρ** [g cm^{-3}] **of THF /** ***HMHC***			
[K]	[g cm^{-3}]	**0.05 M**	**0.10 M**	**0.25 M**	**0.50 M**	**0.05 M**	**0.10 M**	**0.25 M**	**0.50 M**
213	*0.9738*	*0.9786*	*0.9822*	*0.9937*	*1.021*	*0.9743*	*0.9747*	*0.9756*	*0.9777*
243	*0.9414*	*0.9463*	*0.9501*	*0.9624*	*0.9892*	*0.9421*	*0.9427*	*0.9444*	*0.9477*
273	0.9090	0.9140	0.918	0.9311	0.9571	0.9099	0.9107	0.9132	0.9177
283	0.8983	0.9033	0.9074	0.9209	0.9464	0.8993	0.9001	0.9029	0.9077
293	0.8875	0.8926	0.8967	0.9103	0.9357	0.8885	0.8894	0.8925	0.8977
303	0.8766	0.8817	0.8859	0.8998	0.925	0.8777	0.8787	0.882	0.8877

7 COMPUTATIONAL DETAILS

The density functional theory (DFT) calculations performed herein used the Perdew-Burke-Ernzerhof (PBE) functional[8] with the Amsterdam Density Functional (ADF)[9-11] software package. The TZP basis set was used for the Na, K, C, N, and O atoms, while the QZ3P + 1 diffuse basis set was used for the H atoms.[12] The core electronic states were kept frozen except for Na and K. All of the geometry optimized structures were checked against the computed analytical frequencies to ensure the prevalence of only real frequencies. Relativistic effects were taken into account using the scalar ZORA method.[13]

Unrestricted DFT optimizations were performed with a set spin state of ½ whenever there was a single alkali metal in the model. If there were zero or two alkali metals in the model, restricted DFT optimizations were performed. The nuclear magnetic resonance (NMR) parameters,[14] electric field gradients (EFG), Hirshfeld charges, and bond orders[15] were calculated from the geometry optimized models. Geometry optimizations were also performed using a THF solvent model (COSMO)[16], and the computed magnitudes of the binding energies, M–M distances, charges, and dipoles (see Section S17) were generally somewhat larger than in the gas phase. The same trend in the binding energies was observed, with the K-HMHC (Na) and K-15-crown-5_2 (Na) being preferred. The basis set superposition error (BSSE) for K-HMHC + Na → K-HMHC (Na), using the counterpoise method, was calculated to be 0.29 kJ/mol, in agreement with previous studies on similar systems.[17]

Multiple conformations for the HMHC species were optimized. The chair-like conformation was found to be the most stable in the gas phase and is, as a result, the major conformer considered herein for interaction with sodium and potassium. The chair-like HMHC conformation is more stable than the boat-like HMHC conformation by 5.9 kJ/mol. The K-HMHC (Na) boat-like conformation is more stable by 0.84 kJ/mol than the K-HMHC (Na) chair-like conformation, with this difference indicating, essentially, isoenergetic models. The effect of the basis set on the superalkali to alkalide binding energy for the chair conformation of K-

---

8 Perdew, J. P.; Burke, K.; Ernzerhof, M. Generalized Gradient Approximation Made Simple. *Phys. Rev. Lett.* **1996**, *77*, 3865–3868.

9 Te Velde, G.; Bickelhaupt, F. M.; Baerends, E. J.; Fonseca Guerra, C.; van Gisbergen, S. J. A.; Snijders, J. G.; Ziegler, T. Chemistry with ADF. *J. Comp. Chem.* **2001**, *22*, 931–967.

10 Fonseca Guerra, C.; Snijders, J. G.; te Velde, G.; Baerends, E. J. Towards an order-N DFT method. *Theor. Chem. Acc.* **1998**, *99*, 391–403.

11 *ADF, SCM, Theoretical Chemistry*; Vrije Universiteit: Amsterdam, The Netherlands, http://www.scm.com.

12 Van Lenthe, E.; Baerends, E. J. Optimized Slater-type basis sets for the elements 1-118. *J. Comp. Chem.* **2003**, *24*, 1142–1156.

13 Van Lenthe, E.; Ehlers, A.; Baerends, E. J. Geometry optimizations in the zero order regular approximation for relativistic effects. *J. Chem. Phys.* **1999**, *110*, 8943–8953.

14 Schreckenbach, G.; Ziegler, T. The calculation of NMR shielding tensors based on density functional theory and the frozen-core approximation. *Int. J. Quant. Chem.* **1996**, *60*, 753–766.

15 Nalewajski, R. F.; Mrozek, J.; Michalak, A. Two-electron valence indices from the Kohn-Sham orbitals. *Int. J. Quant. Chem.* **1997**, *61*, 589–601.

16 Klamt, A. Conductor-like screening model for real solvents: A new approach to the quantitative calculation of solvation phenomena. *J. Phys. Chem.* **1995**, *99*, 2224–2235.

17 Zurek, E. Alkali metals in ethylenediamine: A computational study of the optical absorption spectra and NMR parameters of $[M(en)_3^{\delta+}\cdot M^{\delta-}]$ ion pairs. *J. Am. Chem. Soc.* **2011**, *133*, 4829–4839.

HMHC (Na) was studied. It further supported the choice of the basis set, with the data shown below.

Basis Set	K-HMHC (Na) Binding Energy (kJ/mol)
DZP	-108.95
TZP	-91.80
TZP, H - QZ3P	-91.04
TZP, H - QZ3P + 1Diffuse	-91.04

The binding energies (BE) were calculated as follows for each system, where *E*[X] is the energy of species X:

BE for HMHC (15-crown-5) Superalkali Models:
S1. BE (M-HMHC) = *E* [M-HMHC] – *E* [M] – *E* [HMHC]

BE for HMHC (15-crown-5) Superalkali-Alkalide Models:
S2. BE (M_1-HMHC (M_2)) = *E* [M_1-HMHC (M_2)] – *E* [M_1] – *E* [M_2] – *E* [HMHC]

BE for HMHC Superalkali Dimer Models:
S3. BE ([M_1-HMHC][M_2-HMHC]) = *E* [M_1-HMHC][M_2-HMHC] – *E* [M_1] – *E* [M_2] – 2·*E*[HMHC]

BE for 15-crown-5_2 Superalkali Models:
S4. BE (M-15-crown-5_2) = *E* [M-15-crown-5_2] – *E* [M] – 2·*E* [15-crown-5]

BE for 15-crown-5_2 Superalkali-Alkalide Models:
S5. BE (M_1-15-crown-5_2 (M_2)) = *E* [M_1-15-crown-5_2 (M_2)] – *E* [M_1] – *E* [M_2] –2·*E* [15-crown-5]

8 STRUCTURE, HOMO, AND LUMO OF THE HMHC MOLECULE

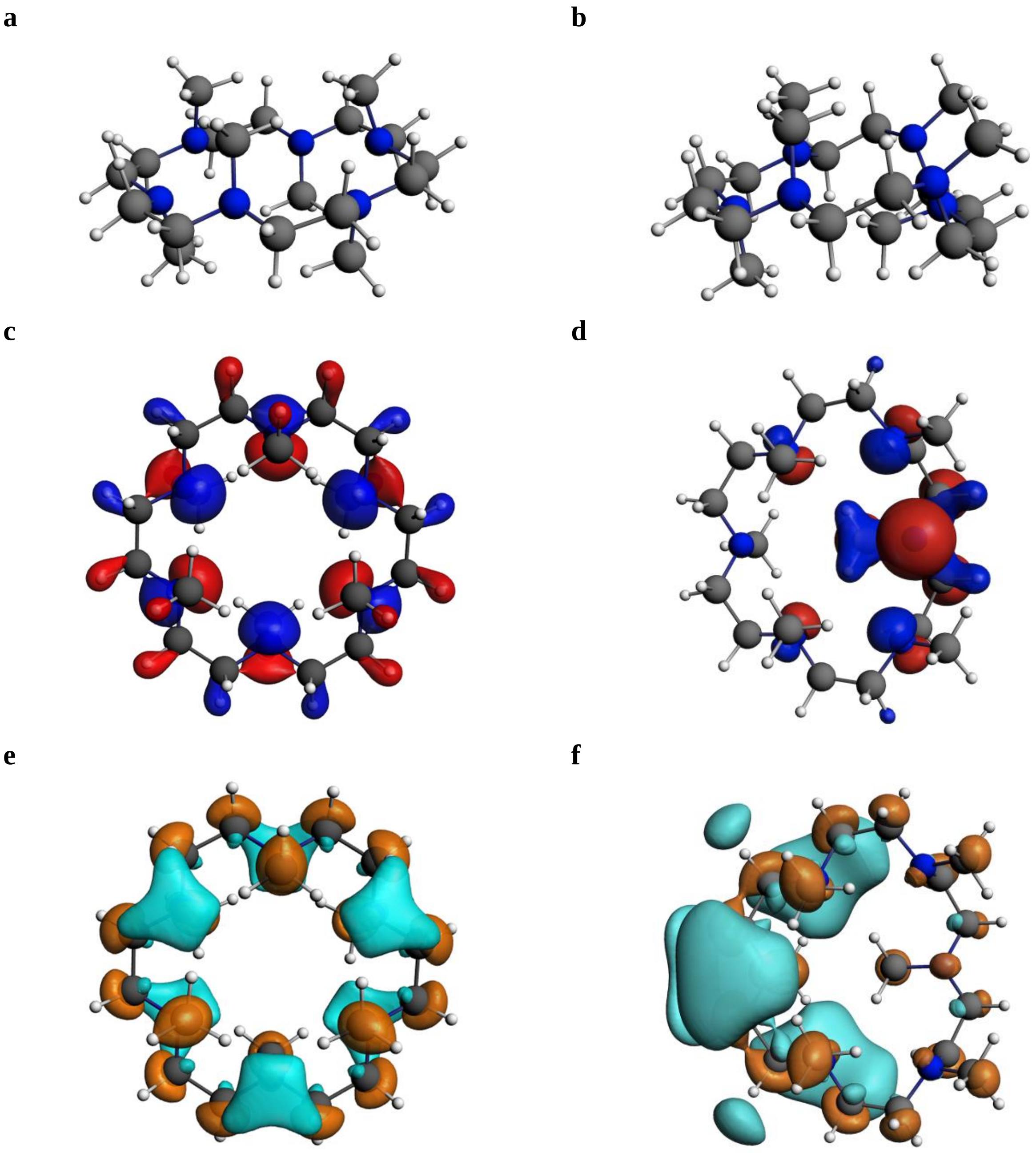

Figure SI-7 | The optimized geometry of the HMHC molecule in the chair (**a**) and boat (**b**) conformations. The HOMO of the HMHC molecule in the chair (**c**) and boat (**d**) conformations (isovalue = +/- 0.030 a.u.) and the LUMO of the HMHC molecule in the chair (**e**) and boat (**f**) conformations (isovalue = +/- 0.015 a.u.). Nitrogen, carbon, and hydrogen are colored blue, gray, and white, respectively.

9 Structure, HOMO, and LUMO of the 15-crown-5 Molecule

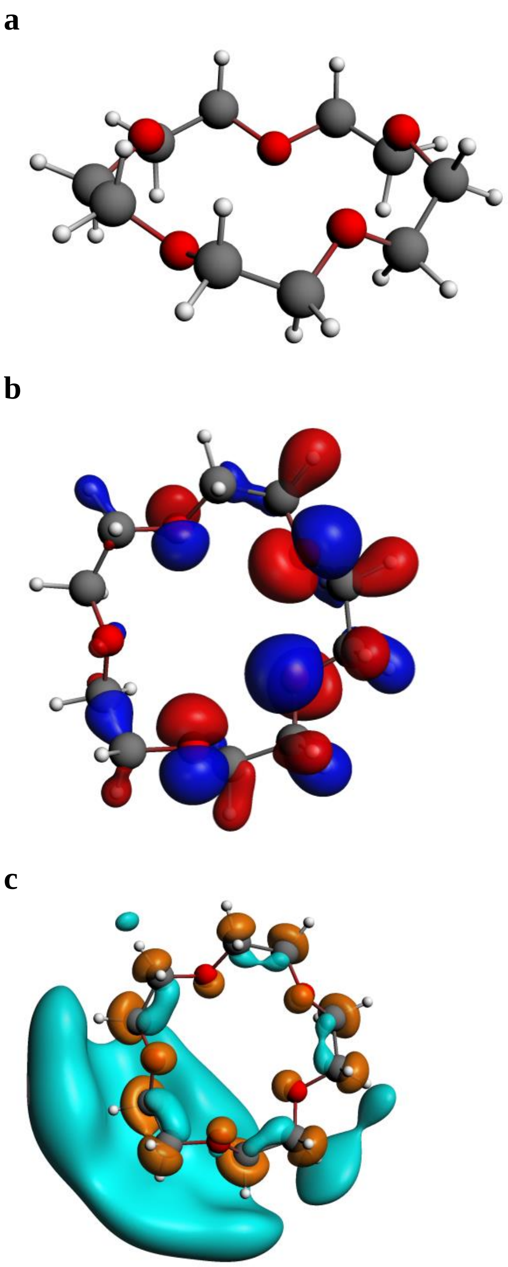

Figure SI-8 | The optimized geometry (**a**), HOMO (**b**, isovalue = +/- 0.030 a.u.), and LUMO (**c**, isovalue = +/- 0.015 a.u.) of the 15-crown-5 molecule. Oxygen, carbon, and hydrogen are colored red, gray, and white, respectively.

10 ALKALI METAL HMHC - STRUCTURE, SOMO, AND HOMO

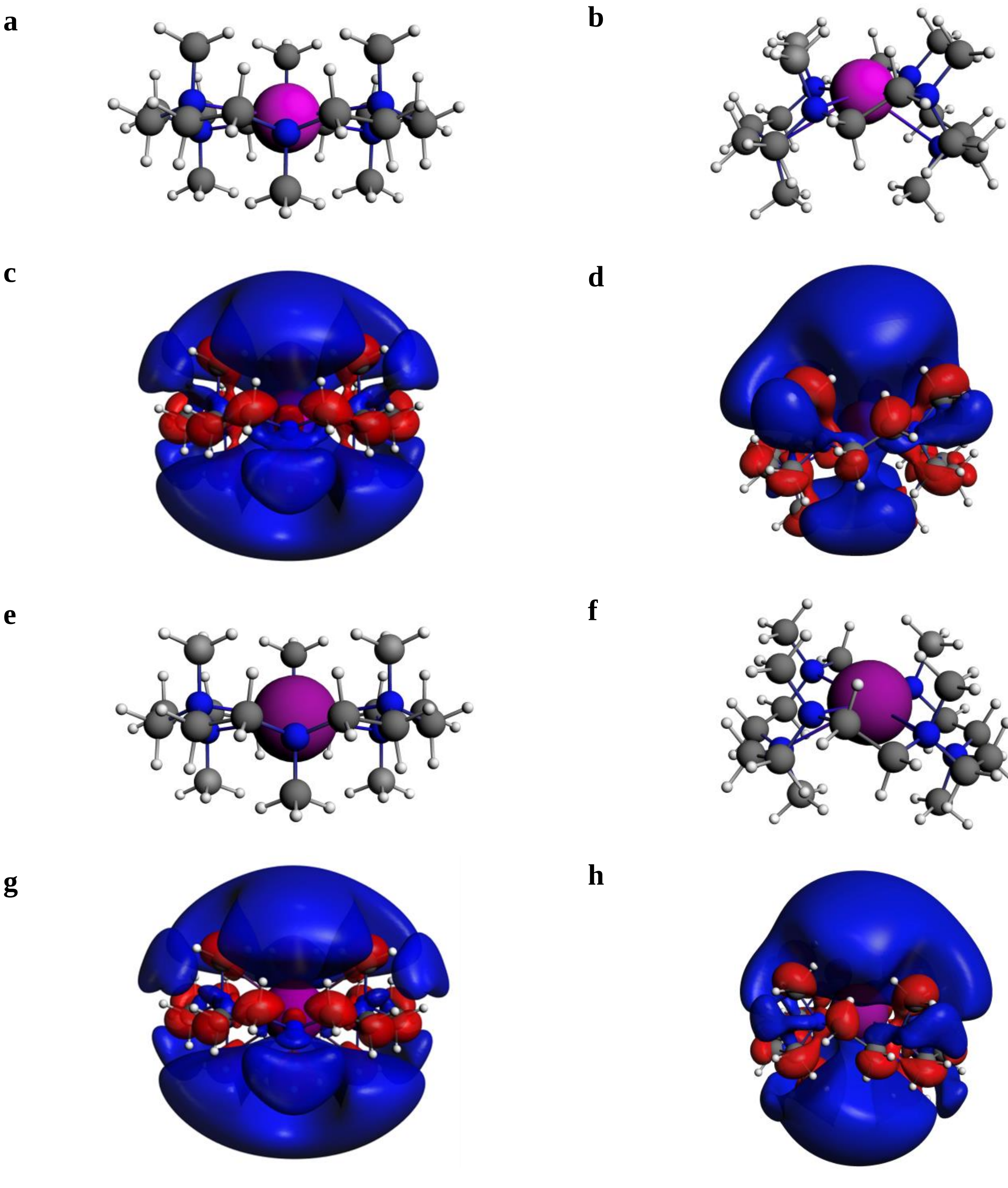

Figure SI-9 | The optimized geometry and SOMO (isovalue = +/- 0.010 a.u.) of Na-HMHC in the chair (**a**, **c**) and boat (**b**, **d**) conformations and of K-HMHC in the chair (**e**, **g**) and boat (**f**, **h**) conformations. Sodium, potassium, nitrogen, carbon, and hydrogen are colored pink, purple, blue, gray, and white, respectively. The SOMO character appears symmetric with HMHC in the chair conformation since the chair superalkalis have C_i symmetry, but not with HMHC in the boat conformation.

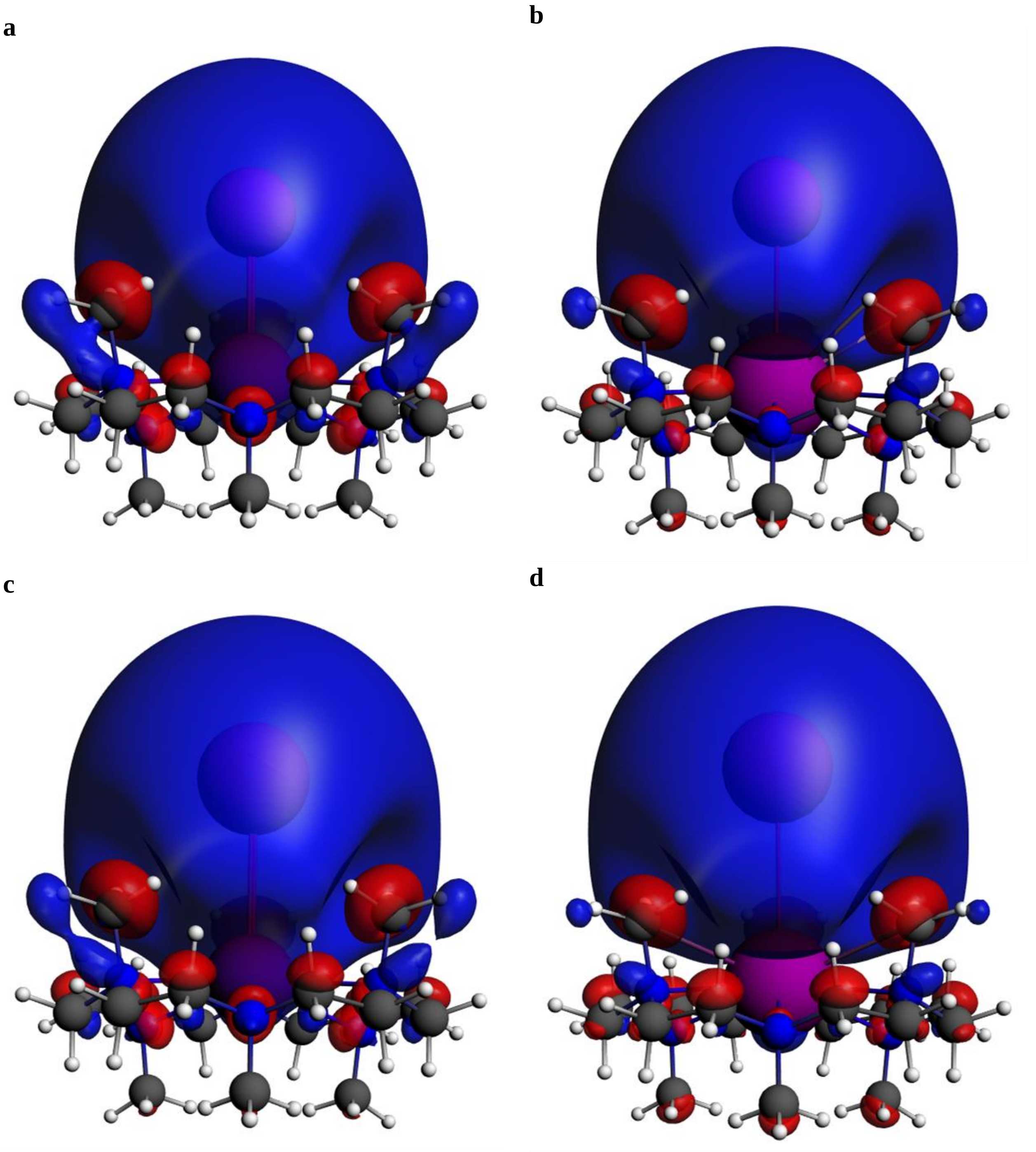

Figure SI-10 | The HOMOs (isovalue = +/- 0.010 a.u.) of superalkali-alkalide Na-HMHC (Na) (**a**), K-HMHC (Na) (**b**), Na-HMHC (K) (**c**), and K-HMHC (K) (**d**). Sodium, potassium, nitrogen, carbon, and hydrogen are colored pink, purple, blue, gray, and white, respectively. The majority of the charge density associated with the HOMO of the M-HMHC (M) superalkali-alkalide pair is found on the alkalide.

a **b**

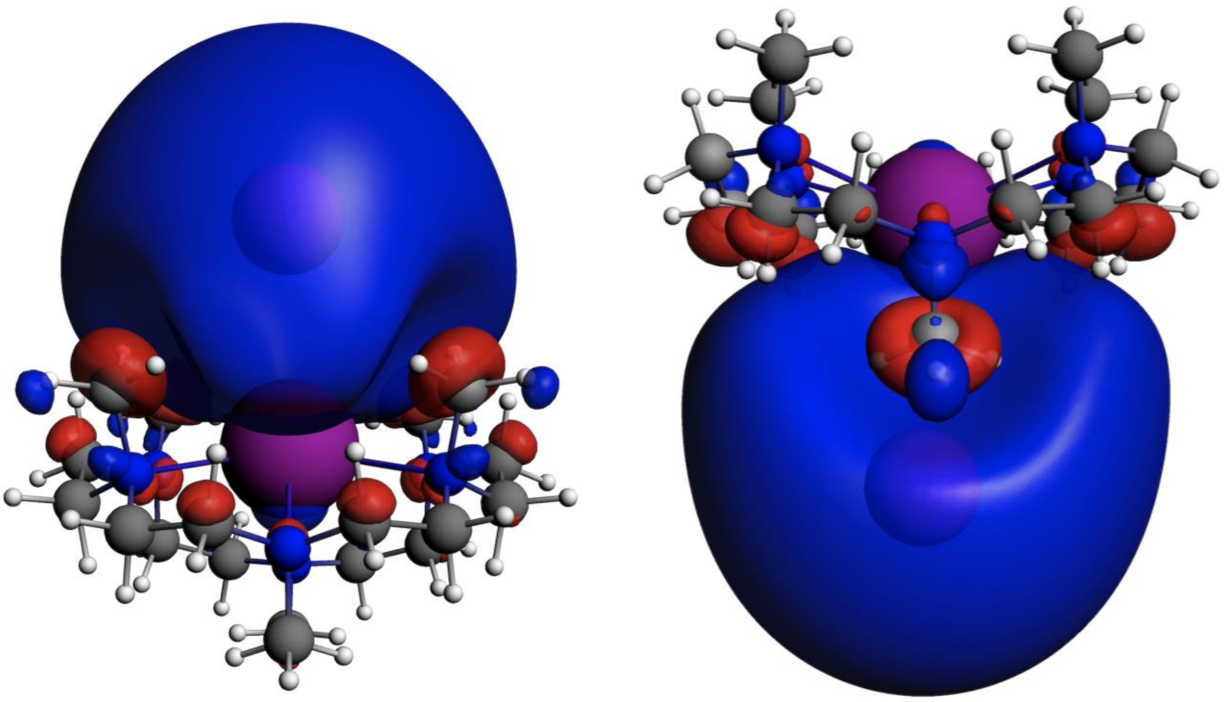

Figure SI-11 | The superalkali-alkalide K-HMHC (Na) HOMO (isovalue = +/- 0.010 a.u.) in the preferred ion pair boat (**a**) and less preferred boat (**b**) conformations. Sodium, potassium, nitrogen, carbon, and hydrogen are colored pink, purple, blue, gray, and white, respectively. Note the majority of the HOMO is located between the K and Na atoms and the region above (a) and below (b) the potassium atom.

11 ALKALI METAL 15-CROWN-5 - STRUCTURE, SOMO, AND HOMO

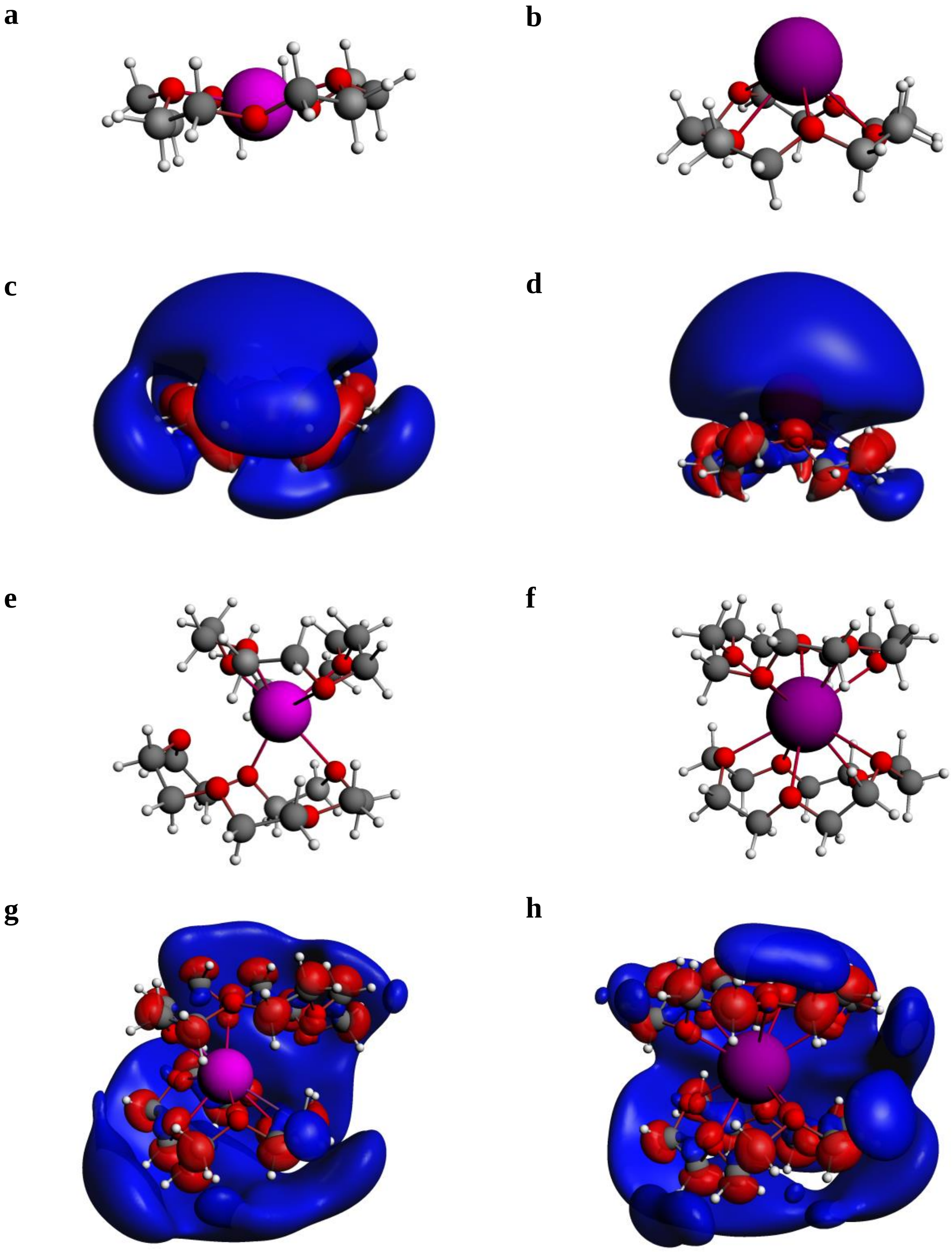

Figure SI-12 | The optimized geometry and SOMO (isovalue = +/- 0.010 a.u.) of the single crown superalkali Na-15C5 (**a**, **c**) and K-15C5 (**b**, **d**) and of the sandwich superalkali Na-15C5$_2$ (**e**, **g**) and K-15C5$_2$ (**f**, **h**). Sodium, potassium, oxygen, carbon, and hydrogen are colored pink, purple, red, gray, and white, respectively.

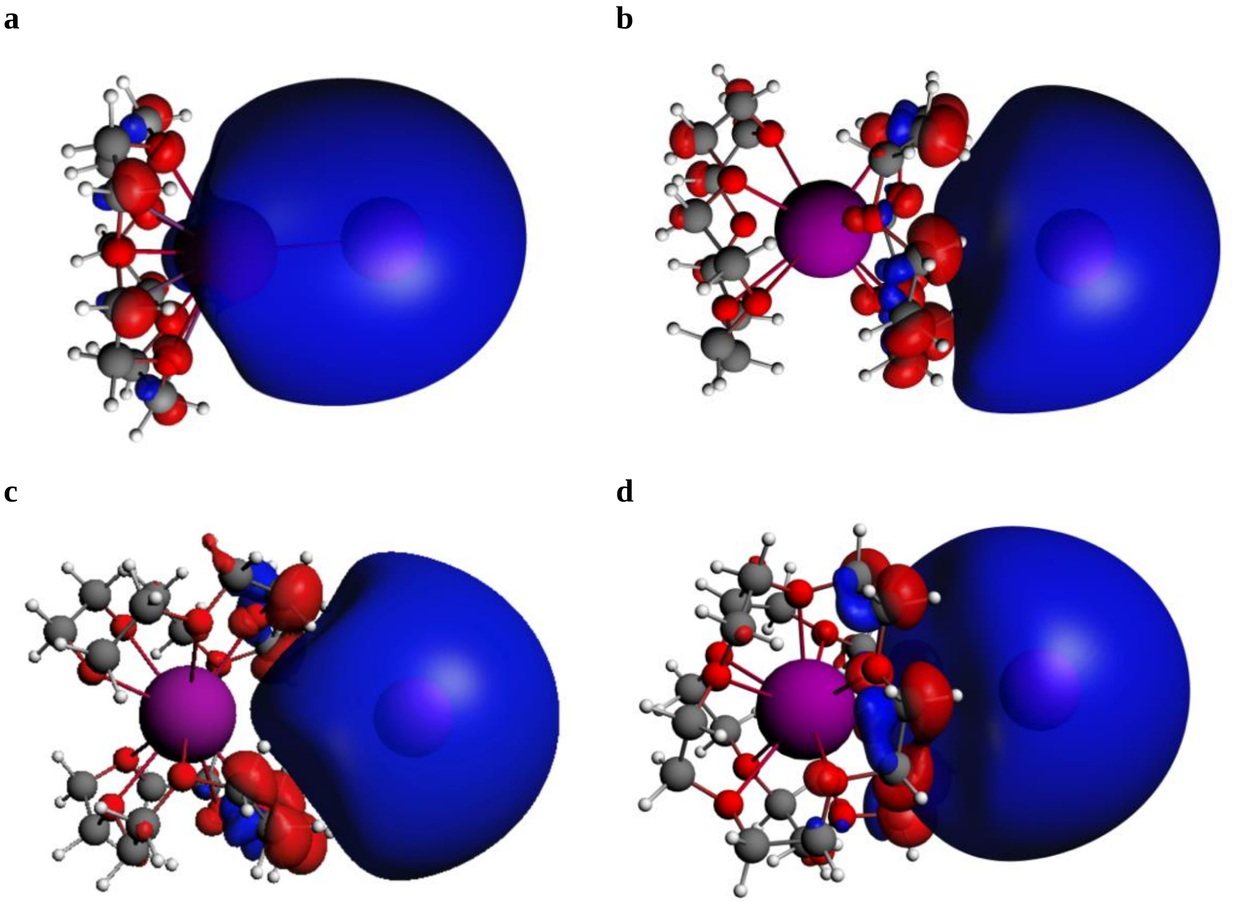

Figure SI-13 | The HOMOs (isovalue = +/- 0.010 a.u.) of the single crown superalkali-alkalide K-15C5 (Na) (**a**), the axial sandwich superalkali-alkalide K-$15C5_2$ (Na) (**b**), and the equatorial sandwich superalkali-alkalide K-$15C5_2$ (Na) in side view (**c**) and top view (**d**). Sodium, potassium, oxygen, carbon, and hydrogen are colored pink, purple, red, gray, and white, respectively. Note the large amount of HOMO character on the Na atom of the sandwich superalkali-alkalide complex and the lack of HOMO character on the sandwiched K atom. In the K-HMHC (Na) superalkali-alkalide models, there is HOMO character on the encapsulated K atom, which is not seen in K-$15C5_2$ (Na).

12 Superalkali Dimer Structures and HOMOs

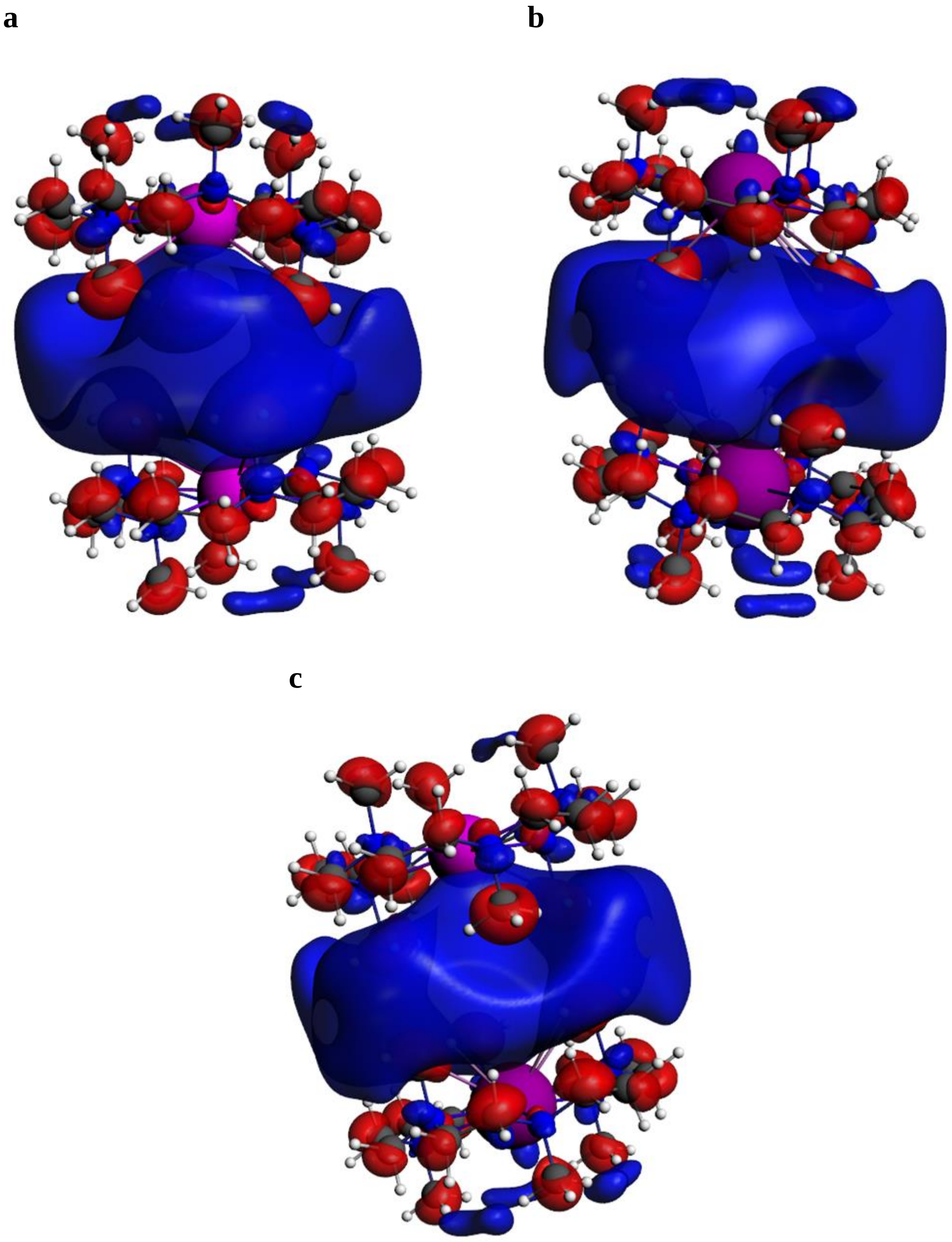

Figure SI-14 | The HOMOs (isovalue = +/- 0.010 a.u.) of the superalkali dimers [Na-HMHC][Na-HMHC] (**a**), [K-HMHC][K-HMHC] (**b**), and [Na-HMHC][K-HMHC] (**c**). Sodium, potassium, nitrogen, carbon, and hydrogen are colored pink, purple, blue, gray, and white, respectively. Note the large amount of HOMO character between the superalkali dimers. The M–M distance is long because the methyl groups on HMHC induce steric repulsion between the two superalkalis (see Sections S13 and S14).

13 COMPUTATIONAL ANALYSIS OF THE ALKALI METAL HMHC MODELS

	Gas-Phase Binding Energy (kJ/mol)	M–M Distance (Å)	Superalkali Hirshfeld Charge (e)	Alkalide Hirshfeld Charge (e)	Dipole Moment (D)
**Superalkali M-HMHC Models**					
Na-HMHC	-62.7	-	+0.31	-	0
K-HMHC	-90.9	-	+0.26	-	0
**Superalkali-Alkalide M-HMHC (M) Models**					
Na-HMHC (Na)	-152.0	3.61	+0.22	-0.23	9.3
K-HMHC (Na)	-177.0	4.11	+0.24	-0.29	10.6
K-HMHC (Na) - Boat	-183.5 [-150.6]	4.00 [4.50]	+0.21 [+0.27]	-0.29 [-0.33]	10.5 [12.6]
Na-HMHC (K)	-130.8	4.36	+0.25	-0.21	9.8
K-HMHC (K)	-159.0	4.73	+0.25	-0.24	9.9
**Superalkali Dimer [M-HMHC][M-HMHC] Models**	**Gas-Phase Binding Energy (kJ/mol)**	**M–M Distance (Å)**	**M1 Hirshfeld Charge (e)**	**M2 Hirshfeld Charge (e)**	**Dipole Moment (D)**
[Na-HMHC][Na-HMHC]	-155.8	7.34	+0.34	+0.34	0.2
[Na-HMHC][K-HMHC]	-184.6	7.39	+0.34	+0.29	0.8
[K-HMHC][K-HMHC]	-213.0	7.80	+0.30	+0.30	0.4
**Free Ion Pair Models**	**Gas-Phase Binding Energy (kJ/mol)**	**M–M Distance (Å)**	**M1 Hirshfeld Charge (e)**	**M2 Hirshfeld Charge (e)**	**Dipole Moment (D)**
Na-Na	-74.0	3.13	0.00	0.00	0
Na-K	-65.5	3.54	-0.10	+0.10	2.5
K-K	-55.5	3.98	0.00	0.00	0

Table SI-4 | The binding energies (kJ/mol), calculated in the gas-phase by subtracting the energy of the alkali metal(s) and of HMHC(s) from the total system energy (see Equations S1, S2, and S3 above), the distance between the alkali metals [M–M] (Å), the Hirshfeld charge of the alkali metal coordinated to HMHC [M-HMHC] (e), the Hirshfeld charge of the alkali metal about the M-HMHC [(M)] (e), and the dipole (D) for each superalkali, superalkali-alkalide ion pair, and superalkali dimer HMHC model. M is the symbol for the alkali metal, representing either Na or K, with '1' and '2' noting the first and second alkali metal type, respectively, for the superalkali dimer and free ion pair models. Each system was modeled with the chair conformation of HMHC, except for the 'K-HMHC (Na) – Boat' model where the most stable and less stable position of the sodide are given outside of, and within the brackets, respectively (Structures/HOMOs/SOMOs: see SI Sections 8, 10, and 12).

	Superalkali Vzz (a.u.)	Alkalide Vzz (a.u.)
**Superalkali M-HMHC Models**		
Na-HMHC	0.185	-
K-HMHC	0.542	-
**Superalkali-Alkalide M-HMHC (M) Models**		
Na-HMHC (Na)	0.041	0.008
K-HMHC (Na)	0.253	0.003
K-HMHC (Na) - Boat	0.178 [0.276]	0.003 [0.008]
Na-HMHC (K)	0.075	0.023
K-HMHC (K)	0.292	0.012
**Superalkali Dimer [M-HMHC][M-HMHC] Models**	**M1 Vzz (a.u.)**	**M2 Vzz (a.u.)**
[Na-HMHC][Na-HMHC]	0.182	0.182
[Na-HMHC][K-HMHC]	0.179	0.528
[K-HMHC][K-HMHC]	0.536	0.536
**Free Ion Pair Models**	**M1 Vzz (a.u.)**	**M2 Vzz (a.u.)**
Na-Na	0.026	0.026
Na-K	0.013	0.048
K-K	0.022	0.022

Table SI-5 | The Vzz component of the electric field gradient tensor (a.u.) for the coordinated alkali metal [M-HMHC] and the alkalide [(M)] in each superalkali and superalkali-alkalide ion pair HMHC model. M is the symbol for the alkali metal, to represent either Na or K, with '1' and '2' noting the first and second alkali metal type, respectively, in the models for the superalkali dimers and free ion pairs.

14 DISCUSSION ON THE HMHC SUPERALKALI DIMER MODELS

The M–M distances in the superalkali dimer models range from 7.34-7.80 Å, whereas in the superalkali-alkalide systems they range from 3.61-4.73 Å. The bonding in the superalkali dimers is associated with the H ⇜⇝ H interaction in the HMHC that results from partial occupation of the LUMO of the HMHC molecules upon addition of alkali metals to the system. The reason for the large distance between the two metals in the superalkali dimers is because the methyl groups of HMHC are bulky, and induce steric repulsion. If the binding energies are calculated by subtracting the coordinated M-HMHC energies from the total energy (Equation S6), instead of fragmenting the M-HMHC into M and HMHC (Equation S3), then each superalkali dimer has a binding energy of -30.4 to -31.3 kJ/mol. A binding energy of -86.1 kJ/mol for the superalkali-alkalide K-HMHC (Na) is computed via Equation S7.

HMHC Superalkali Dimer:

S6. BE ($[M_1$-HMHC$][M_2$-HMHC$]$) = E $[M_1$-HMHC$][M_2$-HMHC$]$ – E $[M_1$-HMHC$]$ – E $[M_2$-HMHC$]$

HMHC Superalkali-Alkalide:

S7. BE (M_1-HMHC (M_2)) = E $[M_1$-HMHC (M_2)$]$ – E $[M_1$-HMHC$]$ – E $[M_2]$

Consider the following three reactions along with their associated change in energies, ΔE, computed in the gas phase (ΔE_{g}) and in THF (ΔE_{THF}):

R1. K + Na + 2 HMHC → K-HMHC + Na-HMHC ΔE_{g} = -153.6 kJ/mol , ΔE_{THF} = -161.0 kJ/mol

R2. K + Na + 2 HMHC → K-HMHC (Na) + HMHC ΔE_{g} = -177.0 kJ/mol , ΔE_{THF} = -214.4 kJ/mol

R3. K + Na + 2 HMHC → [Na-HMHC][K-HMHC] ΔE_{g} = -184.6 kJ/mol , ΔE_{THF} = -206.9 kJ/mol

R3, the formation of the superalkali dimer, is preferred in the gas phase, whereas R2, the formation of the superalkali-alkalide ion pair, is preferred in solvent. However, the difference in the ΔEs for the formation of the dimer and the ion pair is quite small, and these species are likely to coexist in equilibrium (K-HMHC (Na) + HMHC ⇌ [Na-HMHC] [K-HMHC]). The finite temperature contributions to the free energy are likely to favor the ion pair because of the entropic contribution.

15 COMPUTATIONAL ANALYSIS OF THE ALKALI METAL 15-CROWN-5 MODELS

	Gas-Phase Binding Energy (kJ/mol)	M–M Distance (Å)	Superalkali Hirshfeld Charge (e)	Alkalide Hirshfeld Charge (e)	Dipole Moment (D)
**Superalkali M-15C5 Single Crown Models**					
Na-15C5	-38.7	-	+0.16	-	2.8
K-15C5	-54.6	-	-0.04	-	7.7
**Superalkali M-15C5$_2$ Sandwich Models**					
Na-15C5$_2$	-84.5	-	+0.26	-	4.5
K-15C5$_2$	-109.7	-	+0.23	-	2.7
**M-15C5 (M) Single Crown Pair Models**					
Na-15C5 (Na)	-141.8	3.38	+0.10	-0.33	11.6
K-15C5 (Na)	-143.0	3.69	+0.09	-0.34	13.9
Na-15C5 (K)	-122.3	3.90	+0.10	-0.29	11.0
K-15C5 (K)	-125.3	4.20	+0.08	-0.28	12.9
**Axial Pair M-15C5$_2$ (M) Models**					
Na-15C5$_2$ (Na)	-165.0	5.51	+0.28	-0.46	19.1
K-15C5$_2$ (Na)	-177.7	6.20	+0.27	-0.43	18.3
Na-15C5$_2$ (K)	-151.7	5.89	+0.28	-0.42	18.5
K-15C5$_2$ (K)	-165.1	6.54	+0.27	-0.39	17.4
**Equatorial Pair M-15C5$_2$ (M) Models**					
Na-15C5$_2$ (Na)	-147.4	5.71	+0.28	-0.45	17.1
K-15C5$_2$ (Na)	-178.2	5.59	+0.28	-0.42	15.6
Na-15C5$_2$ (K)	-132.9	6.38	+0.28	-0.42	17.0
K-15C5$_2$ (K)	-166.7	7.08	+0.28	-0.43	17.9

Table SI-6 | The binding energies (kJ/mol), calculated in the gas-phase by subtracting the lone alkali metal(s) and 15C5(s) energies from the total system energy (see Equations S1, S2, S4, and S5), the distance between the alkali metals [M–M] (Å), the Hirshfeld charge of the alkali metal coordinated to 15C5(s) [M-15C5(s)] (e), the Hirshfeld charge of the alkali metal about the M-15C5(s) [(M)] (e), and the dipole (D) for each superalkali and superalkali-alkalide pair model. M is the symbol for the alkali metal, to represent either Na or K (Structures/HOMOs/SOMOs: see SI Sections 9 and 11).

	Superalkali Vzz (a.u.)	Alkalide Vzz (a.u.)
**Superalkali M-15C5 Single Crown Models**		
Na-15C5	0.287	-
K-15C5	0.160	-
**Superalkali M-15C5$_2$ Sandwich Models**		
Na-15C5$_2$	0.047	-
K-15C5$_2$	0.125	-
**M-15C5 (M) Single Crown Pair Models**		
Na-15C5 (Na)	0.056	0.007
K-15C5 (Na)	0.188	0.001
Na-15C5 (K)	0.047	0.014
K-15C5 (K)	0.156	0.005
**Axial Pair M-15C5$_2$ (M) Models**		
Na-15C5$_2$ (Na)	0.064	0.002
K-15C5$_2$ (Na)	0.113	0.002
Na-15C5$_2$ (K)	0.066	0.002
K-15C5$_2$ (K)	0.115	0.002
**Equatorial Pair M-15C5$_2$ (M) Models**		
Na-15C5$_2$ (Na)	0.124	0.002
K-15C5$_2$ (Na)	0.165	0.002
Na-15C5$_2$ (K)	0.105	0.005
K-15C5$_2$ (K)	0.063	0.003

Table SI-7 | The Vzz component of the electric field gradient tensor (a.u.) for the coordinated alkali metal [M-15C5(s)] and the exterior alkali metal [(M)] in each model. M is the symbol for the alkali metal, to represent either Na or K.

Consider the following two reactions along with their associated change in energies in the gas phase, ΔE_{g},

R4. K + Na + 2(15-crown-5) → K-15-crown-5 (Na) + 15-crown-5 ΔE_{g} = -143.0 kJ/mol
R5. K + Na + 2(15-crown-5) → K-15-crown-5$_2$ (Na) ΔE_{g} = -178.2 kJ/mol

The coordination of two 15-crown-5 molecules to K is clearly favored energetically to an extent that is likely to overcome the entropic penalty to the free energy for R5 at the temperatures used.

16 Examination of HMHC Intermediates for the Chair/Boat Conformations

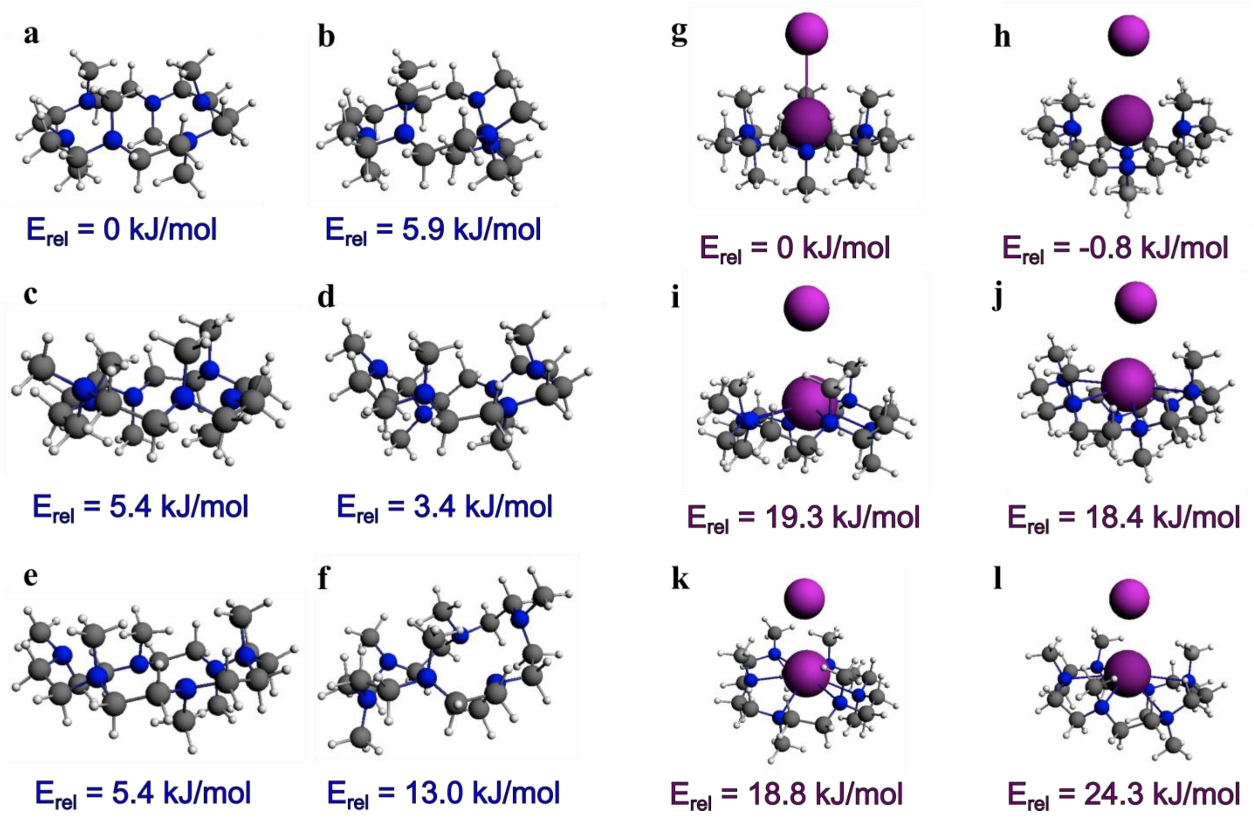

Figure SI-15 | Proposed intermediates in the interconversion of HMHC conformations. (**a**, **b**) The structure of HMHC in the chair (**a**) and boat (**b**) conformations with the relative energy (E_{rel}) given with respect to the chair conformation. (**c**-**f**) Proposed intermediate structures in the interconversion of HMHC from the chair to the boat conformation obtained by reorienting the methyl groups and carrying out geometry optimizations. Note that these do not represent transition states and therefore the actual barrier might be higher. The energies given are with respect to the chair conformation of HMHC in (**a**). (**g**, **h**) The structure of K-HMHC (Na) in the chair (**g**) and boat (**h**) conformations with the relative energy (E_{rel}) given with respect to the chair conformation. (**i**-**l**) Proposed intermediate structures in the interconversion of K-HMHC (Na) from the chair to the boat conformation obtained by reorienting the methyl groups and carrying out geometry optimizations. Note that these do not represent transition states and therefore the actual barrier might be higher. The energies given are with respect to the chair conformation of K-HMHC (Na) in (**g**). A positive relative energy notes a structure less stable than the reference 0 kJ/mol structure. A negative relative energy notes a structure more stable than the reference 0 kJ/mol structure.

Discussion on Chair and Boat Conformations for Pure HMHC and K-HMHC (Na)

Pure HMHC slightly favors the chair conformation by 5.9 kJ/mol. K-HMHC (Na) is, essentially, isoenergetic when comparing the chair and boat conformations as the relative energy is -0.84 kJ/mol in favor of the boat.

We investigated the potential energy landscape of HMHC by flipping one methyl group at a time for converting between the chair and boat conformations of HMHC. Note that the systems obtained above are not transition states, so the barriers may be higher. Pure HMHC has the highest energy intermediate at 13 kJ/mol while K-HMHC (Na) has its highest energy intermediate at 24 kJ/mol, both relative to the chair conformation. The highest energy intermediate, shown in Figure SI-15, is the intermediate structure just before attaining the boat conformation for both pure HMHC and K-HMHC (Na). Given that the highest energy intermediate along the potential energy surface between the chair and boat conformations is lower for pure HMHC, with an energetic preference for the chair, then more chair than boat conformations are present for pure HMHC in solution. The highest energy intermediate is still low for K-HMHC (Na), but the implication is that the shift between the chair and boat is more difficult to attain for the ion pair. Lastly, since the energies of the chair and boat conformations for K-HMHC (Na) are essentially isoenergetic, more K-HMHC (Na) in the chair conformation is, most likely, observed in solution as pure HMHC would be in the chair conformation prior to coordination of K and Na into a superalkali-alkalide HMHC ion pair.

Further, the chair K-HMHC (Na) sodide was found to have a computed shielding that is 1 ppm more shielded and upfield than the boat K-HMHC (Na) sodide. There is likely to be a dynamic equilibrium between the two species in solution. Since the NMR signal in experiment, shown in Figure 7a in the main text, that is more upfield is also the most intense, this potentially supports the prevalence of more K-HMHC (Na) in the chair conformation than the boat conformation.

17 COMPUTATIONAL STUDY ON THE INTERACTION OF THF WITH NA AND K

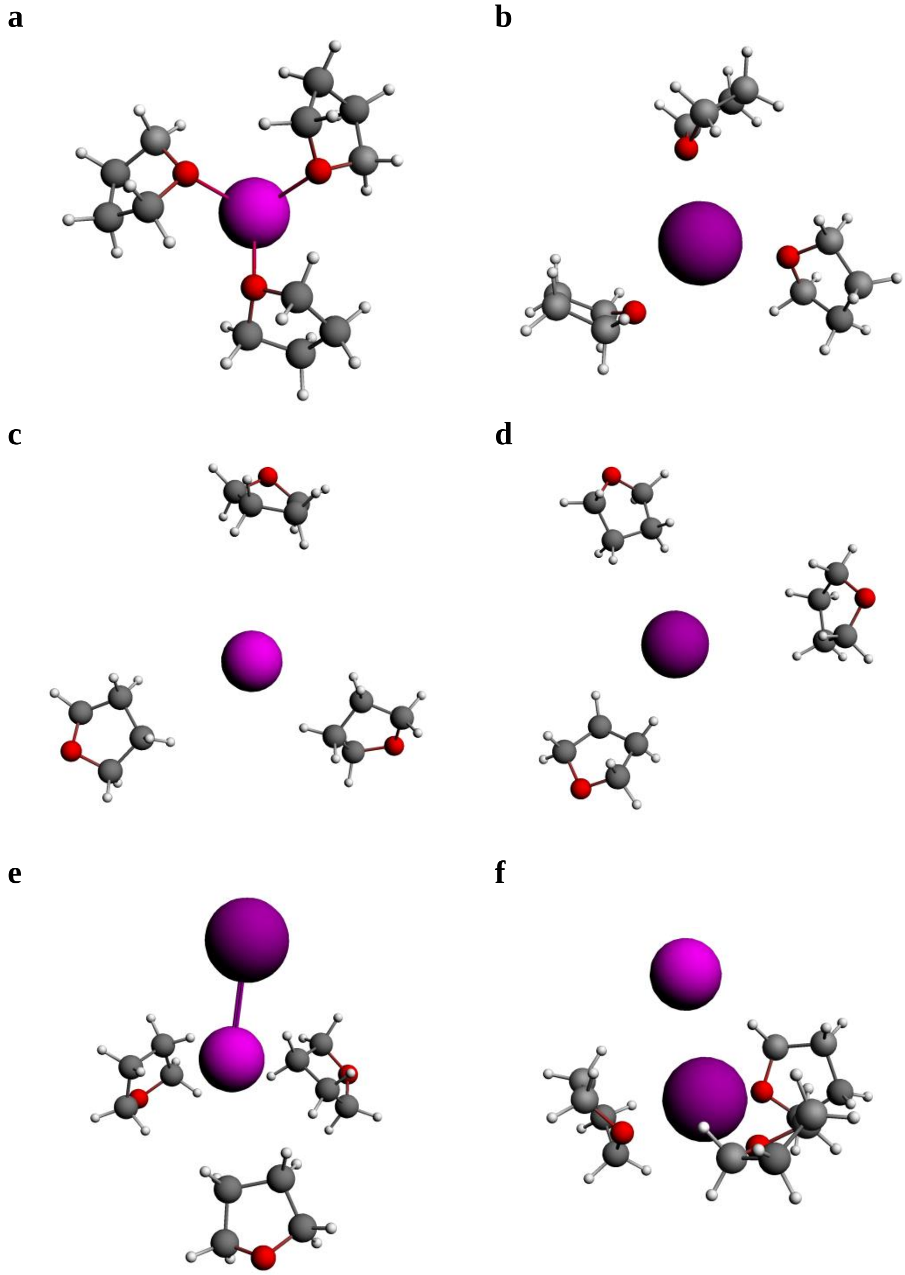

Figure SI-16 | (**a**, **b**) The structure of sodium (**a**) / potassium (**b**) interacting with three THF molecules, with the oxygen atoms in THF facing the alkali metal. (**c**, **d**) The structure of sodium (**c**) / potassium (**d**) interacting with three THF molecules, with the oxygen atoms facing away from the alkali metal. (**e**) The structure of K-Na, with three THF molecules interacting through sodium similar to that shown in (**c**). (**f**) The structure of K-Na, with three THF molecules interacting through potassium similar to that shown in (**b**). The binding energies for the formation of these species, and their EFG parameters, are given in Table SI-8 and SI-9 below.

Generally, the gas phase binding energy to THF was calculated as being weaker than to HMHC (see Table SI-8).

	Gas-Phase Binding Energy (kJ/mol)	M1 Hirshfeld Charge (e)	M2 Hirshfeld Charge (e)	Dipole Moment (D)
**Alkali HMHC Models**				
Na-HMHC (chair)	-62.7	+0.31	-	0
K-HMHC (chair)	-90.9	+0.26	-	0
Na-HMHC (boat)	-92.8	+0.15	-	8.0
K-HMHC (boat)	-106.8	+0.19	-	1.2
K-HMHC (Na) (chair)	-177.0	+0.24	-0.29	10.6
K-HMHC (Na) (boat)	-183.5 [-150.6]	+0.21 [+0.27]	-0.29 [-0.33]	10.5 [12.6]
**Alkali THF Adduct Models**				
a. Na-THF	-73.2	+0.01	-	5.4
b. K-THF	-61.4	+0.14	-	2.0
c. Na-THF	-3.6	+0.09	-	1.5
d. K-THF	-3.0	+0.12	-	1.6
e. (K) Na - THF	-76.0	+0.16	-0.04	5.8
f. K (Na) - THF	-158.8	+0.23	-0.26	6.5

Table SI-8 | The binding energies (kJ/mol), calculated in the gas-phase by subtracting the lone alkali metal(s) and HMHC/THF energies from the total system energy, the Hirshfeld charge of the alkali metal that appears first in the system name [M1] (e), the Hirshfeld charge of the alkali metal that appears second in the system name [M2] (e), and the dipole moment (D) for each system. M is the symbol for the alkali metal, to represent either Na or K. For the ‘K-HMHC (Na) (boat)’ model, the most stable and less stable position of the sodide are given outside and within the brackets, respectively. The structures for each Alkali THF Adduct Model are given in Figure SI-16, with each letter next to a particular model in the table corresponding to that structure.

	M1 Vzz (a.u.)	M2 Vzz (a.u.)
K-HMHC (Na) (chair)	0.253	0.003
K-HMHC (Na) (boat)	0.178 [0.276]	0.003 [0.008]
e. (K) Na - THF	0.059	0.012
f. K (Na) - THF	0.113	0.004

Table SI-9 | The Vzz component of the electric field gradient tensor (a.u.) for the ion pair models with HMHC and THF. M is the symbol for the alkali metal to represent either Na or K, with ‘1’ and ‘2’ noting the first and second alkali metal type, respectively, in the model. Structures (e) and (f) in the table correspond to a structure in Figure SI-16.

Implicit THF Solvent	Binding Energy (kJ/mol)	M–M Distance (Å)	Superalkali Hirshfeld Charge (e)	Alkalide Hirshfeld Charge (e)	Dipole Moment (D)
**Superalkali M-HMHC Models**					
Na-HMHC	-67.2	-	+0.28	-	0
K-HMHC	-93.8	-	+0.23	-	0
**Superalkali-Alkalide M-HMHC (M) Models**					
Na-HMHC (Na)	-179.0	4.78	+0.34	-0.60	21.0
K-HMHC (Na)	-214.4	4.36	+0.27	-0.55	19.1
Na-HMHC (K)	-154.6	5.67	+0.35	-0.59	21.9
K-HMHC (K)	-189.3	5.18	+0.28	-0.51	19.2
**Superalkali Dimer [M-HMHC][M-HMHC] Models**	**Binding Energy (kJ/mol)**	**M–M Distance (Å)**	**M1 Hirshfeld Charge (e)**	**M2 Hirshfeld Charge (e)**	**Dipole Moment (D)**
[Na-HMHC][Na-HMHC]	-177.6	7.74	+0.34	+0.32	13.0
[Na-HMHC][K-HMHC]	-206.9	7.73	+0.34	+0.27	13.9
[K-HMHC][K-HMHC]	-233.3	8.59	+0.30	+0.29	9.2
**Free Ion Pair Models**	**Binding Energy (kJ/mol)**	**M–M Distance (Å)**	**M1 Hirshfeld Charge (e)**	**M2 Hirshfeld Charge (e)**	**Dipole Moment (D)**
Na-K	-79.1	3.67	-0.24	+0.24	6.1

Table SI-10 | The binding energies (kJ/mol), calculated with a THF implicit solvent, were found by subtracting the lone alkali metal(s) and HMHC(s) energies from the total system energy (see Equations S1, S2, and S3), the distance between alkali metals [M–M] (Å), the Hirshfeld charge of the alkali metal coordinated to HMHC [M-HMHC] (e), the Hirshfeld charge of the alkali metal about the M-HMHC [(M)] (e), and the dipole (D) for each superalkali, superalkali-alkalide ion pair, and superalkali dimer HMHC model. M is the symbol for the alkali metal, representing either Na or K, with ‘1’ and ‘2’ noting the first and second alkali metal type, respectively, for the superalkali dimer and free ion pair models. Each system given was modeled with the chair conformation of HMHC and is similar to the Figures shown in SI Sections 8, 10, and 12.

Implicit THF Solvent	Binding Energy (kJ/mol)	M–M Distance (Å)	Superalkali Hirshfeld Charge (e)	Alkalide Hirshfeld Charge (e)	Dipole Moment (D)
**Superalkali M-15C5 Single Crown Models**					
Na-15C5	-38.0	-	-0.17	-	8.6
K-15C5	-55.1	-	-0.23	-	12.7
**Superalkali M-15C5$_2$ Sandwich Models**					
Na-15C5$_2$	-16.8	-	+0.26	-	17.8
K-15C5$_2$	-44.7	-	+0.23	-	19.4
**M-15C5 (M) Single Crown Pair Models**					
Na-15C5 (Na)	-153.6	3.50	+0.10	-0.47	17.3
K-15C5 (Na)	-165.0	3.80	+0.12	-0.50	19.8
Na-15C5 (K)	-134.1	4.07	+0.08	-0.39	15.7
K-15C5 (K)	-140.3	4.31	+0.07	-0.41	17.7
**Axial Pair M-15C5$_2$ (M) Models**					
Na-15C5$_2$ (Na)	-149.7	6.83	+0.28	-0.82	32.3
K-15C5$_2$ (Na)	-167.2	7.22	+0.29	-0.80	34.7
Na-15C5$_2$ (K)	-124.2	7.30	+0.29	-0.75	31.6
K-15C5$_2$ (K)	-140.7	7.91	+0.29	-0.77	35.1
**Equatorial Pair M-15C5$_2$ (M) Models**					
Na-15C5$_2$ (Na)	-127.9	7.67	+0.30	-0.81	33.9
K-15C5$_2$ (Na)	-164.5	7.33	+0.29	-0.79	31.4
Na-15C5$_2$ (K)	-101.6	7.84	+0.30	-0.76	31.9
K-15C5$_2$ (K)	-139.9	7.60	+0.29	-0.74	30.1

Table SI-11 | The binding energies (kJ/mol), calculated with a THF implicit solvent by subtracting the lone alkali metal(s) and 15C5(s) energies from the total system energy (see Equations S1, S2, S4, and S5), the distance between the alkali metals [M–M] (Å), the Hirshfeld charge of the alkali metal coordinated to 15C5(s) [M-15C5(s)] (e), the Hirshfeld charge of the alkali metal about the M-15C5(s) [(M)] (e), and the dipole (D) for each superalkali and superalkali-alkalide pair model. M is the symbol for the alkali metal, to represent either Na or K (Structures/HOMOs/SOMOs: see SI Sections 9 and 11).

18 Superalkali-Alkalide HOMOs with Small Isovalues

a **b**

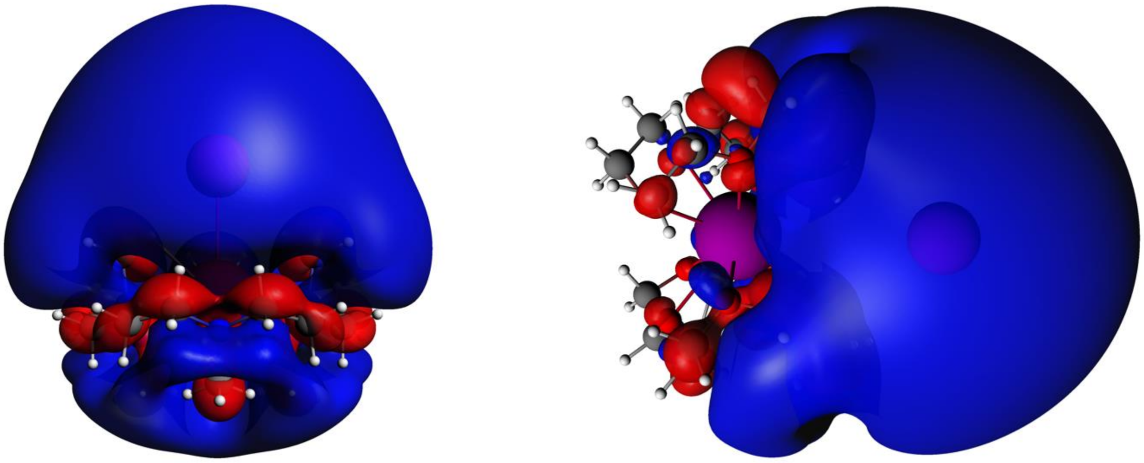

Figure SI-17 | (a) The K-HMHC (Na) HOMO (Isovalue = +/- 0.003 a.u.) and **(b)** the K-15-crown-5_2 (Na) HOMO (Isovalue = +/- 0.003 a.u.). Note the pieces of isosurface that interconnect two H atoms: these are the H ⇜⇝ H interactions discussed in the text that result from orbital overlap.

19 FRAGMENT ORBITAL ANALYSIS

Superalkali-Alkalide HOMO

**K-HMHC (Na) SFO**	**Orbital %**
Na 3s	51.75%
K-HMHC SOMO	34.24%
K-HMHC LUMO	9.46%
K-HMHC LUMO+5	2.26%

**$K-(15C5)_2$ (Na) SFO**	**Orbital %**
Na 3s	67.93%
$K-(15C5)_2$ SOMO	20.04%
$K-(15C5)_2$ LUMO	3.87%
$K-(15C5)_2$ LUMO+1	2.01%
$K-(15C5)_2$ LUMO+2	2.10%
$K-(15C5)_2$ LUMO+6	1.63%

Table SI-12 | Results of a fragment orbital (SFO) analysis that was used to analyze the composition of the HOMO of the complexes in terms of the occupied and unoccupied orbitals of the fragments (the spin-restricted K-HMHC / K-15-Crown-5_2 superalkalis, and the spin-restricted Na atoms). A TZP basis was employed on all of the atoms except H, which was treated with a QZ3P + 1Diffuse basis.

Superalkali-Alkalide HOMO (H TZP)

**K-HMHC (Na) SFO**	**Orbital %**
Na 3s	68.10%
K-HMHC SOMO	27.69%
K-HMHC LUMO	2.92%
K-HMHC LUMO+5	0%

**$K-(15C5)_2$ (Na) SFO**	**Orbital %**
Na 3s	80.29%
$K-(15C5)_2$ SOMO	14.48%
$K-(15C5)_2$ LUMO	2.31%
Na 4s	1.52%
$K-(15C5)_2$ LUMO+1	0%
$K-(15C5)_2$ LUMO+2	0%
$K-(15C5)_2$ LUMO+6	0%

Table SI-13 | Same as Table SI-12, except a TZP basis was employed on all atoms.

20 COORDINATES (XYZ)

HMHC – Chair Conformation

N	-1.961005	-1.159760	-1.788450
N	-0.306130	1.102266	-2.651685
N	0.994870	2.663129	-0.538660
N	1.961005	1.159760	1.788450
N	0.306130	-1.102266	2.651685
N	-0.994870	-2.663129	0.538660
C	-2.868454	-0.328551	-1.008174
C	0.868774	0.275175	-2.894804
C	-0.261711	3.040788	0.095520
C	-2.164648	-3.111703	-0.209434
C	-2.238009	-2.585547	-1.651169
C	-1.901083	-0.770444	-3.193389
C	-1.465852	0.683447	-3.431808
C	-0.043586	2.526376	-2.829190
C	1.073989	3.082778	-1.933947
C	2.868454	0.328551	1.008174
C	-0.868774	-0.275175	2.894804
C	0.261711	-3.040788	-0.095520
C	2.164648	3.111703	0.209434
C	2.238009	2.585547	1.651169
C	1.901083	0.770444	3.193389
C	1.465852	-0.683447	3.431808
C	0.043586	-2.526376	2.829190
C	-1.073989	-3.082778	1.933947
H	-2.802660	-0.612289	0.047646
H	-3.927158	-0.412886	-1.341089
H	-2.553257	0.717788	-1.080051
H	1.233768	0.338791	-3.944284
H	0.631155	-0.768030	-2.661296
H	1.672984	0.580855	-2.217265
H	-1.095460	2.600779	-0.461970
H	-0.403749	4.143204	0.151704
H	-0.296296	2.624118	1.107777
H	-3.055683	-2.786905	0.347404
H	-2.227343	-4.223574	-0.272159
H	-1.511455	-3.123711	-2.277350
H	-3.246160	-2.861527	-2.040532
H	-2.880283	-0.899674	-3.711818
H	-1.198418	-1.452452	-3.694196
H	-1.299653	0.790750	-4.529527
H	-2.294000	1.362310	-3.180890
H	-0.981926	3.062271	-2.624212

H	0.233483	2.778421	-3.879746
H	2.051395	2.758567	-2.320143
H	1.049935	4.192362	-2.045125
H	2.802660	0.612289	-0.047646
H	3.927158	0.412886	1.341089
H	2.553257	-0.717788	1.080051
H	-1.233768	-0.338791	3.944284
H	-0.631155	0.768030	2.661296
H	-1.672984	-0.580855	2.217265
H	1.095460	-2.600779	0.461970
H	0.403749	-4.143204	-0.151704
H	0.296296	-2.624118	-1.107777
H	3.055683	2.786905	-0.347404
H	2.227343	4.223574	0.272159
H	1.511455	3.123711	2.277350
H	3.246160	2.861527	2.040532
H	2.880283	0.899674	3.711818
H	1.198418	1.452452	3.694196
H	1.299653	-0.790750	4.529527
H	2.294000	-1.362310	3.180890
H	0.981926	-3.062271	2.624212
H	-0.233483	-2.778421	3.879746
H	-2.051395	-2.758567	2.320143
H	-1.049935	-4.192362	2.045125

HMHC – Boat Conformation

N	2.003161	-4.371253	-5.730003
N	-0.026677	-2.134924	-5.617609
N	0.199109	0.281062	-3.866801
N	2.966116	-0.480155	-2.921884
N	3.884740	-2.666993	-1.032776
N	3.309251	-4.819856	-3.039411
C	3.088845	-3.786119	-6.506633
C	-0.875236	-2.721933	-4.587043
C	-0.350575	0.907434	-2.671028
C	3.486179	-5.710415	-4.184851
C	2.347716	-5.693801	-5.215990
C	0.745923	-4.391797	-6.472994
C	0.151267	-3.008373	-6.776215
C	-0.479546	-0.812742	-6.052896
C	-0.808781	0.171071	-4.919260
C	3.056116	-1.428893	-4.029686
C	3.487871	-2.140338	0.266403
C	2.095008	-5.102516	-2.282191
C	1.416184	0.947518	-4.324745
C	2.610862	0.866101	-3.366568

C	4.200965	-0.411750	-2.141424
C	4.797360	-1.763971	-1.731638
C	4.422409	-4.020342	-0.907703
C	4.518357	-4.822987	-2.213606
H	3.990906	-3.705311	-5.890483
H	3.331549	-4.383586	-7.414294
H	2.822554	-2.770618	-6.819032
H	-1.920051	-2.896195	-4.927631
H	-0.881716	-2.065416	-3.710671
H	-0.447622	-3.677363	-4.265287
H	-0.628755	1.974019	-2.826961
H	0.379227	0.853354	-1.854683
H	-1.248598	0.362800	-2.351436
H	3.603534	-6.773503	-3.866504
H	4.432741	-5.436120	-4.673579
H	2.648032	-6.395352	-6.029636
H	1.442516	-6.122045	-4.764467
H	0.023019	-4.976561	-5.887781
H	0.845334	-4.916413	-7.452311
H	0.793132	-2.477072	-7.494722
H	-0.813434	-3.192759	-7.306262
H	0.301221	-0.397462	-6.708262
H	-1.403233	-0.874607	-6.675824
H	-1.742903	-0.148678	-4.438349
H	-1.031909	1.152428	-5.397740
H	2.089956	-1.490717	-4.542639
H	3.850268	-1.152527	-4.762911
H	3.251524	-2.433792	-3.640844
H	4.349752	-1.985385	0.953123
H	2.969464	-1.183322	0.137868
H	2.788283	-2.836519	0.746610
H	1.227133	-5.029846	-2.946694
H	2.098479	-6.110712	-1.811318
H	1.968833	-4.337879	-1.508487
H	1.706571	0.499861	-5.282835
H	1.234959	2.029996	-4.523221
H	2.430173	1.483049	-2.473016
H	3.459586	1.351006	-3.907196
H	5.010203	0.108945	-2.709219
H	4.008231	0.209158	-1.252942
H	5.699879	-1.544260	-1.113985
H	5.154835	-2.274407	-2.634055
H	5.436835	-4.032005	-0.445998
H	3.768442	-4.562261	-0.211389
H	4.810234	-5.856208	-1.909879
H	5.343082	-4.446129	-2.837731

Na-HMHC – Chair Conformation

Na	0.000000	0.000000	0.000000
N	-2.040424	-1.115991	-1.752672
N	-0.222309	1.053066	-2.701950
N	0.918523	2.716770	-0.500038
N	2.040424	1.115991	1.752672
N	0.222309	-1.053066	2.701950
N	-0.918523	-2.716770	0.500038
C	-3.154611	-0.383057	-1.137106
C	0.943499	0.329557	-3.226828
C	-0.259328	3.364849	0.091923
C	-2.147925	-3.096874	-0.209789
C	-2.231241	-2.568494	-1.638165
C	-1.892698	-0.765744	-3.172231
C	-1.449212	0.672874	-3.416477
C	-0.039258	2.507206	-2.814283
C	1.061103	3.068283	-1.919875
C	3.154611	0.383057	1.137106
C	-0.943499	-0.329557	3.226828
C	0.259328	-3.364849	-0.091923
C	2.147925	3.096874	0.209789
C	2.231241	2.568494	1.638165
C	1.892698	0.765744	3.172231
C	1.449212	-0.672874	3.416477
C	0.039258	-2.507206	2.814283
C	-1.061103	-3.068283	1.919875
H	-3.236013	-0.637102	-0.071452
H	-4.126076	-0.608499	-1.624783
H	-2.980260	0.699814	-1.199589
H	1.126147	0.550250	-4.299287
H	0.801854	-0.754137	-3.116182
H	1.845905	0.597944	-2.660818
H	-1.172332	3.046928	-0.429763
H	-0.197823	4.472223	0.043645
H	-0.368498	3.070450	1.144623
H	-2.997484	-2.718614	0.376351
H	-2.264200	-4.201388	-0.255261
H	-1.462930	-3.051939	-2.258101
H	-3.213444	-2.882040	-2.053444
H	-2.842661	-0.920611	-3.728377
H	-1.160182	-1.459583	-3.608613
H	-1.335688	0.805428	-4.514202
H	-2.242815	1.364619	-3.100260
H	-0.999085	2.980853	-2.563792
H	0.195506	2.805584	-3.859008
H	2.038999	2.694983	-2.255558

H	1.078771	4.170309	-2.064162
H	3.236013	0.637102	0.071452
H	4.126076	0.608499	1.624783
H	2.980260	-0.699814	1.199589
H	-1.126147	-0.550250	4.299287
H	-0.801854	0.754137	3.116182
H	-1.845905	-0.597944	2.660818
H	1.172332	-3.046928	0.429763
H	0.197823	-4.472223	-0.043645
H	0.368498	-3.070450	-1.144623
H	2.997484	2.718614	-0.376351
H	2.264200	4.201388	0.255261
H	1.462930	3.051939	2.258101
H	3.213444	2.882040	2.053444
H	2.842661	0.920611	3.728377
H	1.160182	1.459583	3.608613
H	1.335688	-0.805428	4.514202
H	2.242815	-1.364619	3.100260
H	0.999085	-2.980853	2.563792
H	-0.195506	-2.805584	3.859008
H	-2.038999	-2.694983	2.255558
H	-1.078771	-4.170309	2.064162

<u>K-HMHC – Chair Conformation</u>

K	0.000000	0.000000	0.000000
N	-2.083899	-1.119711	-1.772788
N	-0.218562	1.069147	-2.753454
N	0.916154	2.767751	-0.505607
N	2.083899	1.119711	1.772788
N	0.218562	-1.069147	2.753454
N	-0.916154	-2.767751	0.505607
C	-3.235376	-0.415519	-1.196315
C	0.937314	0.363989	-3.319508
C	-0.237528	3.467546	0.071577
C	-2.157236	-3.104508	-0.209588
C	-2.231417	-2.578234	-1.643908
C	-1.897962	-0.764853	-3.188725
C	-1.464689	0.681623	-3.432370
C	-0.041975	2.527461	-2.830179
C	1.067704	3.081429	-1.935071
C	3.235376	0.415519	1.196315
C	-0.937314	-0.363989	3.319508
C	0.237528	-3.467546	-0.071577
C	2.157236	3.104508	0.209588
C	2.231417	2.578234	1.643908
C	1.897962	0.764853	3.188725

C	1.464689	-0.681623	3.432370
C	0.041975	-2.527461	2.830179
C	-1.067704	-3.081429	1.935071
H	-3.348586	-0.670486	-0.134018
H	-4.183914	-0.665128	-1.715716
H	-3.088970	0.671757	-1.253448
H	1.095903	0.607484	-4.390656
H	0.805735	-0.722768	-3.230365
H	1.852319	0.624357	-2.770335
H	-1.162341	3.171734	-0.442199
H	-0.139091	4.570726	-0.000145
H	-0.357453	3.201895	1.130518
H	-2.995275	-2.700454	0.376011
H	-2.311568	-4.204804	-0.255549
H	-1.441197	-3.044295	-2.249478
H	-3.197170	-2.926091	-2.071523
H	-2.829576	-0.932148	-3.772360
H	-1.146635	-1.450812	-3.605025
H	-1.386216	0.820188	-4.532920
H	-2.253687	1.366645	-3.090817
H	-1.001415	2.991346	-2.560631
H	0.181331	2.853545	-3.869616
H	2.038504	2.680871	-2.259943
H	1.110934	4.179449	-2.105503
H	3.348586	0.670486	0.134018
H	4.183914	0.665128	1.715716
H	3.088970	-0.671757	1.253448
H	-1.095903	-0.607484	4.390656
H	-0.805735	0.722768	3.230365
H	-1.852319	-0.624357	2.770335
H	1.162341	-3.171734	0.442199
H	0.139091	-4.570726	0.000145
H	0.357453	-3.201895	-1.130518
H	2.995275	2.700454	-0.376011
H	2.311568	4.204804	0.255549
H	1.441197	3.044295	2.249478
H	3.197170	2.926091	2.071523
H	2.829576	0.932148	3.772360
H	1.146635	1.450812	3.605025
H	1.386216	-0.820188	4.532920
H	2.253687	-1.366645	3.090817
H	1.001415	-2.991346	2.560631
H	-0.181331	-2.853545	3.869616
H	-2.038504	-2.680871	2.259943
H	-1.110934	-4.179449	2.105503

Na-HMHC (Na)

Na	7.529111	6.348905	-1.783648
Na	4.728591	8.088118	-0.307174
N	2.181262	7.341709	-1.671979
N	3.943982	9.529382	-2.895193
N	5.092408	11.032679	-0.480584
N	6.454395	9.540566	1.827528
N	4.463291	7.353620	2.618935
N	3.243626	5.486439	0.520095
C	1.133939	8.106020	-0.988900
C	5.003242	8.977239	-3.748542
C	3.909534	11.621756	0.153527
C	1.956595	5.321417	-0.161992
C	1.931059	5.892812	-1.577466
C	2.251940	7.690922	-3.101651
C	2.621264	9.142311	-3.396787
C	4.065048	10.987379	-2.795012
C	5.203528	11.450261	-1.889632
C	7.748440	8.975837	1.426579
C	3.303579	8.111940	3.094764
C	4.238689	4.546614	-0.009922
C	6.328556	11.448867	0.206114
C	6.448090	10.996930	1.659271
C	6.129274	9.166691	3.207380
C	5.681265	7.715580	3.363609
C	4.255516	5.904036	2.776310
C	3.096425	5.323909	1.969723
H	1.108429	7.842922	0.074741
H	0.125486	7.925119	-1.419114
H	1.348593	9.179105	-1.049553
H	4.938802	9.370548	-4.786459
H	4.946139	7.882214	-3.772191
H	5.992051	9.208774	-3.334263
H	3.001233	11.290825	-0.362665
H	3.931723	12.732593	0.146424
H	3.829783	11.283478	1.192945
H	1.181831	5.806150	0.446302
H	1.666541	4.250310	-0.235153
H	2.694699	5.392846	-2.189171
H	0.944469	5.632432	-2.020692
H	1.281615	7.487864	-3.606633
H	2.994166	7.022976	-3.560538
H	2.544875	9.283736	-4.496778
H	1.874687	9.812096	-2.950716
H	3.111782	11.386039	-2.424270
H	4.227869	11.452942	-3.791521

H	6.156765	11.056594	-2.268710
H	5.259782	12.558359	-1.969349
H	7.958453	9.203285	0.374400
H	8.577682	9.363245	2.058057
H	7.726724	7.881122	1.489781
H	3.084652	7.927327	4.168620
H	3.472644	9.185911	2.956065
H	2.414797	7.848273	2.510129
H	5.217732	4.721240	0.452641
H	3.935878	3.490235	0.159928
H	4.389916	4.710723	-1.083690
H	7.170794	11.046500	-0.373760
H	6.431573	12.556521	0.195337
H	5.613714	11.406226	2.243621
H	7.370737	11.460303	2.072219
H	6.996639	9.317407	3.886622
H	5.339382	9.838342	3.568004
H	5.551205	7.525699	4.452209
H	6.481244	7.045741	3.019082
H	5.191546	5.409691	2.481561
H	4.077500	5.643208	3.843268
H	2.157234	5.803185	2.275259
H	3.003047	4.252713	2.253104

Na-HMHC (K)

K	8.088210	6.137024	-2.114170
Na	4.658794	8.120515	-0.284726
N	2.196207	7.321683	-1.667441
N	3.886721	9.558961	-2.828476
N	5.041813	11.059021	-0.451317
N	6.384991	9.556861	1.820932
N	4.479096	7.333865	2.615853
N	3.287752	5.510762	0.507613
C	1.159883	8.058508	-0.938631
C	4.968246	8.996796	-3.648329
C	3.841875	11.590792	0.200642
C	2.017459	5.278321	-0.187542
C	1.970276	5.867744	-1.595097
C	2.222351	7.702668	-3.090074
C	2.576758	9.163430	-3.356808
C	3.998554	11.019782	-2.754085
C	5.130835	11.504755	-1.852196
C	7.651524	8.977392	1.355341
C	3.309974	8.089869	3.072658
C	4.343499	4.648031	-0.038638
C	6.265322	11.499531	0.240418

C	6.399077	11.016665	1.682763
C	6.115442	9.167823	3.208874
C	5.687444	7.710740	3.367987
C	4.275358	5.885266	2.782110
C	3.136868	5.298497	1.951000
H	1.173932	7.779007	0.121334
H	0.141052	7.868001	-1.337963
H	1.355734	9.135666	-0.992369
H	4.926758	9.367489	-4.695508
H	4.918322	7.900468	-3.654908
H	5.947372	9.245686	-3.219514
H	2.942851	11.235391	-0.316387
H	3.821667	12.701561	0.214261
H	3.784470	11.231141	1.234616
H	1.211108	5.708745	0.421619
H	1.792688	4.193201	-0.275773
H	2.736881	5.390438	-2.222208
H	0.984547	5.597988	-2.034560
H	1.238996	7.505550	-3.571160
H	2.953091	7.048189	-3.586245
H	2.513554	9.324901	-4.454859
H	1.816495	9.816588	-2.907401
H	3.040729	11.417076	-2.391793
H	4.159519	11.468180	-3.758391
H	6.093895	11.147711	-2.244081
H	5.152950	12.615266	-1.914228
H	7.824542	9.223668	0.299926
H	8.515256	9.336108	1.956343
H	7.614971	7.881416	1.401439
H	3.085982	7.919414	4.147510
H	3.471513	9.163247	2.918988
H	2.427378	7.807548	2.487109
H	5.306104	4.862771	0.442527
H	4.105423	3.570336	0.093773
H	4.494418	4.851353	-1.106558
H	7.118540	11.137720	-0.350824
H	6.334539	12.609751	0.254229
H	5.577433	11.424171	2.287348
H	7.333462	11.457492	2.093534
H	7.005119	9.323455	3.857173
H	5.331182	9.828731	3.602219
H	5.552848	7.521998	4.456244
H	6.497596	7.048446	3.031222
H	5.220934	5.392442	2.514752
H	4.073850	5.631993	3.846620
H	2.182856	5.745510	2.262584

H	3.068150	4.217026	2.200410

<u>K-HMHC (Na)</u>

Na	7.915627	6.197295	-2.056712
K	4.718296	8.082375	-0.285698
N	2.144988	7.309898	-1.692148
N	3.889326	9.558058	-2.807607
N	5.050591	11.107458	-0.450884
N	6.373546	9.553380	1.809641
N	4.488107	7.321337	2.660140
N	3.285215	5.527246	0.509917
C	1.070253	8.032765	-1.008731
C	4.996265	8.980380	-3.583616
C	3.857874	11.650556	0.202115
C	2.014143	5.281949	-0.183291
C	1.951735	5.852351	-1.601205
C	2.227224	7.691453	-3.112752
C	2.592659	9.154897	-3.366598
C	3.998409	11.021123	-2.750589
C	5.130896	11.532556	-1.858512
C	7.612405	8.950366	1.298092
C	3.327248	8.069781	3.146103
C	4.356586	4.689820	-0.048576
C	6.279561	11.524412	0.244865
C	6.409775	11.015373	1.681423
C	6.137156	9.160748	3.204270
C	5.712924	7.702046	3.383226
C	4.283604	5.869584	2.796349
C	3.141581	5.299473	1.953238
H	1.044046	7.757307	0.051942
H	0.071888	7.824793	-1.448812
H	1.250368	9.112971	-1.057366
H	4.993793	9.335484	-4.635852
H	4.944310	7.883878	-3.582121
H	5.964164	9.229717	-3.129891
H	2.954403	11.304393	-0.313193
H	3.848710	12.761192	0.215515
H	3.797323	11.292448	1.236444
H	1.208590	5.714943	0.425173
H	1.794801	4.194801	-0.258312
H	2.725780	5.381572	-2.223440
H	0.971587	5.551581	-2.032884
H	1.263598	7.498780	-3.634113
H	2.974347	7.037007	-3.583386
H	2.556142	9.314857	-4.465998
H	1.821362	9.808394	-2.936913

H	3.038436	11.417914	-2.393286
H	4.156766	11.457146	-3.760847
H	6.096504	11.183303	-2.250142
H	5.137437	12.641906	-1.941603
H	7.759242	9.197031	0.238471
H	8.504036	9.287369	1.867834
H	7.561407	7.854696	1.341937
H	3.124040	7.887697	4.222819
H	3.482931	9.145118	3.000991
H	2.433661	7.792496	2.575026
H	5.319101	4.917067	0.427543
H	4.144097	3.607093	0.076828
H	4.499545	4.898682	-1.116758
H	7.129901	11.167932	-0.353212
H	6.361229	12.633286	0.283109
H	5.597103	11.428123	2.294546
H	7.352528	11.437267	2.092693
H	7.042200	9.316700	3.830700
H	5.363177	9.823251	3.615060
H	5.603972	7.525614	4.476267
H	6.516365	7.036970	3.036829
H	5.227668	5.379625	2.520105
H	4.079856	5.591508	3.854069
H	2.189066	5.744161	2.272059
H	3.071562	4.215388	2.189425

K-HMHC (K)

K	8.427450	5.849553	-2.139469
K	4.707511	8.090505	-0.262939
N	2.114300	7.358250	-1.680585
N	3.932147	9.521706	-2.846134
N	5.120134	11.089515	-0.492889
N	6.403091	9.538011	1.826307
N	4.433684	7.336185	2.666846
N	3.222556	5.534347	0.507058
C	1.078894	8.121311	-0.979604
C	4.990217	8.880507	-3.639783
C	3.946884	11.735444	0.101079
C	1.931796	5.338227	-0.165348
C	1.867583	5.909220	-1.582760
C	2.178923	7.732595	-3.103864
C	2.604546	9.177066	-3.371357
C	4.117402	10.978883	-2.815337
C	5.261959	11.447687	-1.915474
C	7.679417	8.942879	1.405919
C	3.267425	8.060947	3.177598

C	4.250906	4.656971	-0.069769
C	6.352807	11.473974	0.216932
C	6.434953	10.998280	1.668320
C	6.090809	9.171091	3.214115
C	5.655896	7.716033	3.396991
C	4.248358	5.878646	2.781294
C	3.096279	5.308057	1.952918
H	1.064476	7.853893	0.083474
H	0.065356	7.944020	-1.398295
H	1.293434	9.194703	-1.039651
H	4.971813	9.212843	-4.699169
H	4.890001	7.787583	-3.613393
H	5.980487	9.101906	-3.220067
H	3.033467	11.410345	-0.410462
H	4.001889	12.843717	0.047823
H	3.846411	11.445999	1.153555
H	1.153395	5.802826	0.454812
H	1.668965	4.260289	-0.234627
H	2.611627	5.407504	-2.217430
H	0.869553	5.645783	-1.997759
H	1.195108	7.581399	-3.600652
H	2.883271	7.043996	-3.591309
H	2.547858	9.337027	-4.470104
H	1.874879	9.865810	-2.924449
H	3.175465	11.433425	-2.480368
H	4.310532	11.384419	-3.831900
H	6.210290	11.017248	-2.268083
H	5.348242	12.549784	-2.041042
H	7.887599	9.171229	0.352465
H	8.528146	9.306346	2.023056
H	7.639482	7.847804	1.472503
H	3.067476	7.840649	4.247921
H	3.416213	9.141685	3.070641
H	2.373735	7.799958	2.598745
H	5.227097	4.839440	0.398921
H	3.995289	3.583247	0.052219
H	4.386749	4.865738	-1.139016
H	7.199322	11.065929	-0.353355
H	6.479529	12.579123	0.222891
H	5.599377	11.419694	2.243172
H	7.360049	11.432886	2.105155
H	6.962051	9.339542	3.883653
H	5.297561	9.841537	3.571374
H	5.536823	7.541529	4.489166
H	6.459448	7.046096	3.059961
H	5.192734	5.404322	2.477888

H	4.070569	5.581952	3.838731
H	2.148558	5.753375	2.284084
H	3.026816	4.224270	2.189302

Na-HMHC, Na-HMHC

Na	9.910731	5.393047	-1.883523
Na	3.754957	8.678719	0.404232
N	9.173660	2.650387	-1.302222
N	10.603988	4.317867	0.721206
N	12.291221	6.448633	-0.498634
N	10.912713	8.035969	-2.603421
N	9.471567	6.358713	-4.601708
N	7.785622	4.205842	-3.428415
N	2.819435	5.949110	0.586390
N	4.223111	7.260624	2.869942
N	5.867428	9.616085	2.081173
N	4.490507	11.503500	0.233162
N	3.075058	10.178523	-2.036838
N	1.401236	7.857757	-1.200415
C	9.198101	2.319852	0.130172
C	10.407322	2.870067	0.879199
C	11.923438	4.684498	1.253202
C	12.349169	6.113402	0.930558
C	12.493281	7.895407	-0.656801
C	12.227461	8.410874	-2.067429
C	10.274728	1.997809	-2.019958
C	9.536017	5.073178	1.390755
C	13.283964	5.686621	-1.264203
C	10.862608	8.375001	-4.032063
C	9.650221	7.809105	-4.763542
C	8.166987	5.979285	-5.163956
C	7.749235	4.545112	-4.857535
C	7.613379	2.755623	-3.271490
C	7.871063	2.251273	-1.855517
C	9.829977	8.697754	-1.862278
C	10.561210	5.613144	-5.241745
C	6.757298	4.943028	-2.682263
C	2.891863	5.345980	1.925077
C	4.089669	5.800964	2.752097
C	5.517377	7.569797	3.495413
C	5.903858	9.044035	3.433896
C	6.032489	11.073200	2.171004
C	5.785896	11.807810	0.857676
C	3.908592	5.467889	-0.273976
C	3.112403	7.839763	3.633638
C	6.900066	9.017448	1.224386

C	4.477985	12.055262	-1.128713
C	3.272982	11.632130	-1.962783
C	1.755917	9.902804	-2.620235
C	1.335158	8.437868	-2.548167
C	1.218808	6.403180	-1.296655
C	1.506270	5.651333	-0.001495
C	3.379992	12.030982	1.032960
C	4.145252	9.538107	-2.813903
C	0.405634	8.461864	-0.309158
H	9.178069	1.219918	0.291860
H	8.272765	2.716728	0.574073
H	10.290834	2.591752	1.949137
H	11.321182	2.371180	0.526791
H	12.656074	3.976351	0.840302
H	11.961732	4.567173	2.357962
H	11.699110	6.823805	1.461692
H	13.373001	6.257804	1.339699
H	13.528782	8.192604	-0.381433
H	11.824971	8.400804	0.055284
H	12.358589	9.514709	-2.048318
H	12.990152	8.019963	-2.756047
H	10.249210	2.272904	-3.082436
H	10.226626	0.891272	-1.946713
H	11.240882	2.327872	-1.616300
H	8.553259	4.813790	0.970285
H	9.673769	6.152874	1.237346
H	9.506161	4.880352	2.484219
H	13.100862	4.609572	-1.156998
H	14.322301	5.897220	-0.932549
H	13.208094	5.929980	-2.331763
H	10.861252	9.475867	-4.187458
H	11.786059	7.997683	-4.493936
H	9.747693	8.087637	-5.835546
H	8.734012	8.295965	-4.397725
H	7.419057	6.678172	-4.761088
H	8.151058	6.104603	-6.268498
H	8.413010	3.842488	-5.381444
H	6.733506	4.395463	-5.283567
H	6.587315	2.436166	-3.556662
H	8.299168	2.261579	-3.974695
H	7.758573	1.145286	-1.867059
H	7.094735	2.635220	-1.177462
H	9.859156	8.409593	-0.801988
H	9.893541	9.805077	-1.921218
H	8.848302	8.392054	-2.252011
H	11.524766	5.888873	-4.793682

H	10.422494	4.534588	-5.090074
H	10.617650	5.805493	-6.333553
H	6.904200	6.028023	-2.783729
H	5.730657	4.701700	-3.032182
H	6.820965	4.713357	-1.608336
H	2.930655	4.236512	1.867485
H	1.959933	5.596622	2.451136
H	4.005436	5.319333	3.750468
H	5.019115	5.420931	2.301407
H	6.281336	6.965013	2.985334
H	5.536071	7.262021	4.563077
H	5.223356	9.636028	4.062617
H	6.914353	9.141041	3.886754
H	7.053359	11.344751	2.516508
H	5.336810	11.438387	2.940241
H	5.894449	12.896120	1.056850
H	6.570290	11.545301	0.133430
H	3.863530	5.955812	-1.258037
H	3.870809	4.368666	-0.427882
H	4.886142	5.715264	0.166654
H	2.157881	7.636430	3.129896
H	3.225741	8.930318	3.698326
H	3.053734	7.435385	4.665391
H	6.748850	7.931788	1.132692
H	7.925740	9.189722	1.614998
H	6.846311	9.430669	0.207163
H	4.499571	13.166936	-1.115379
H	5.405435	11.731569	-1.623180
H	3.395882	12.076680	-2.973948
H	2.357430	12.070429	-1.541287
H	1.022223	10.527035	-2.090396
H	1.712467	10.206552	-3.688640
H	1.984550	7.834278	-3.198200
H	0.310139	8.363695	-2.972371
H	0.184378	6.142898	-1.610512
H	1.885309	6.040089	-2.091922
H	1.393945	4.566429	-0.216374
H	0.743195	5.899132	0.750237
H	3.398965	11.595573	2.040650
H	3.419329	13.135956	1.132199
H	2.419136	11.759787	0.576026
H	5.125298	9.728273	-2.354252
H	4.009408	8.447935	-2.832671
H	4.179190	9.901173	-3.862402
H	0.574626	9.543935	-0.230303
H	-0.633460	8.296941	-0.663533

H	0.494485	8.039646	0.700354

Na-HMHC, K-HMHC

K	9.931098	5.409438	-2.782431
Na	3.964135	8.564266	0.215849
N	8.437545	2.969497	-1.964276
N	9.973692	4.639219	0.056086
N	12.286845	6.210345	-1.127859
N	11.494521	7.770130	-3.619893
N	9.990121	6.102969	-5.674799
N	7.694226	4.533524	-4.460516
N	2.371292	6.217079	1.065695
N	3.841426	7.865033	3.076030
N	6.060276	9.501269	1.949206
N	5.288157	11.009699	-0.500700
N	3.795441	9.415938	-2.526780
N	1.598033	7.782453	-1.357733
C	8.230952	2.897113	-0.508845
C	9.446916	3.293103	0.331683
C	11.274649	4.811790	0.718933
C	12.034256	6.077921	0.315649
C	12.748649	7.573382	-1.433409
C	12.781630	7.919464	-2.924308
C	9.384836	1.947539	-2.417695
C	9.023944	5.678858	0.469224
C	13.249148	5.206187	-1.590200
C	11.698375	7.868052	-5.073128
C	10.487699	7.465752	-5.919577
C	8.683443	5.921024	-6.326376
C	7.929264	4.656830	-5.906683
C	7.183315	3.188865	-4.149680
C	7.145601	2.846401	-2.658493
C	10.517469	8.761169	-3.150679
C	10.952928	5.094758	-6.127311
C	6.782004	5.576911	-3.977495
C	2.169054	6.071841	2.512940
C	3.365329	6.503113	3.355872
C	5.108434	8.080175	3.788675
C	5.837064	9.360827	3.394309
C	6.555215	10.856453	1.666891
C	6.573364	11.214425	0.184793
C	3.377958	5.267270	0.573252
C	2.842161	8.869383	3.455802
C	6.996430	8.481620	1.457266
C	5.496354	11.171731	-1.947794
C	4.297865	10.770021	-2.800639

C	2.501618	9.236718	-3.198827
C	1.770327	7.956925	-2.806667
C	1.109988	6.420728	-1.098584
C	1.087377	6.033199	0.376878
C	4.266212	11.937459	-0.002981
C	4.760570	8.395224	-2.956648
C	0.686684	8.791128	-0.806995
H	7.934508	1.873145	-0.191885
H	7.382636	3.552766	-0.262282
H	9.152400	3.185333	1.398685
H	10.265096	2.578600	0.162837
H	11.882755	3.924642	0.490697
H	11.164431	4.838821	1.825206
H	11.467227	6.964018	0.634506
H	12.983117	6.089319	0.895406
H	13.771016	7.753278	-1.034137
H	12.083544	8.272754	-0.906924
H	13.167941	8.958850	-3.007754
H	13.511459	7.277262	-3.437240
H	9.551608	2.035510	-3.499211
H	9.027539	0.918147	-2.205930
H	10.356244	2.080910	-1.923400
H	8.062464	5.554693	-0.050267
H	9.404159	6.676421	0.204160
H	8.827746	5.659978	1.562049
H	12.874843	4.195698	-1.380964
H	14.239795	5.316002	-1.101054
H	13.392646	5.284054	-2.675636
H	11.975466	8.902047	-5.374134
H	12.557141	7.231732	-5.329947
H	10.773647	7.606884	-6.985063
H	9.656488	8.159402	-5.728138
H	8.073719	6.806281	-6.096222
H	8.783502	5.890193	-7.433429
H	8.488571	3.766364	-6.227885
H	6.973148	4.646815	-6.474463
H	6.151599	3.048580	-4.540456
H	7.816841	2.465277	-4.682149
H	6.733994	1.817002	-2.570367
H	6.431149	3.507806	-2.146169
H	10.334567	8.647631	-2.072668
H	10.851528	9.803511	-3.336854
H	9.549233	8.618054	-3.650375
H	11.906928	5.210719	-5.596502
H	10.577883	4.086153	-5.910586
H	11.152682	5.162299	-7.217318

H	7.202958	6.575258	-4.166854
H	5.784468	5.522328	-4.462190
H	6.635942	5.491774	-2.890403
H	1.931607	5.020830	2.786753
H	1.288085	6.669479	2.786699
H	3.079183	6.385729	4.424023
H	4.208576	5.818939	3.182332
H	5.750976	7.211196	3.586651
H	4.954495	8.108056	4.889442
H	5.260671	10.235651	3.727715
H	6.795692	9.383658	3.956524
H	7.586198	11.000395	2.056782
H	5.914752	11.562766	2.213875
H	6.913590	12.269357	0.099803
H	7.325043	10.602888	-0.336505
H	3.548482	5.407745	-0.503238
H	3.078347	4.211215	0.743009
H	4.343580	5.435103	1.070212
H	1.914861	8.716288	2.888552
H	3.209959	9.876515	3.220301
H	2.598352	8.833211	4.538312
H	6.592074	7.474069	1.632546
H	7.989737	8.551397	1.949718
H	7.145341	8.584541	0.372466
H	5.751848	12.222764	-2.204498
H	6.370479	10.563382	-2.224365
H	4.594126	10.884291	-3.865932
H	3.467966	11.470802	-2.632105
H	1.879090	10.110557	-2.958864
H	2.617036	9.227472	-4.304365
H	2.325522	7.084125	-3.179202
H	0.791664	7.955453	-3.334277
H	0.081389	6.276787	-1.495962
H	1.758526	5.728622	-1.655055
H	0.740981	4.978541	0.437710
H	0.336826	6.636411	0.906968
H	4.107173	11.784884	1.072776
H	4.545884	12.999012	-0.165624
H	3.308558	11.753147	-0.507693
H	5.721146	8.528087	-2.437825
H	4.393960	7.390045	-2.702282
H	4.951735	8.432568	-4.050328
H	1.080068	9.798583	-0.994847
H	-0.329687	8.727314	-1.249153
H	0.597502	8.669603	0.280385

K-HMHC, K-HMHC

K	10.052137	5.112248	-1.868374
K	3.638037	8.950756	0.356067
N	9.490916	2.230138	-1.389143
N	10.833453	3.971188	0.726258
N	12.322221	6.367858	-0.432254
N	10.641278	7.951156	-2.403507
N	9.283069	6.246762	-4.512938
N	7.833965	3.847435	-3.358501
N	2.952241	6.084093	0.430042
N	4.131552	7.459033	2.869648
N	5.729071	9.947028	2.174214
N	4.291247	11.854317	0.299054
N	3.124185	10.449456	-2.137481
N	1.494456	7.966504	-1.459768
C	9.607543	1.870481	0.033067
C	10.799170	2.500873	0.758642
C	12.138285	4.450351	1.207501
C	12.401905	5.940303	0.973400
C	12.310154	7.837460	-0.509871
C	11.939729	8.403381	-1.883200
C	10.581829	1.647921	-2.176415
C	9.732548	4.545644	1.511282
C	13.419404	5.804129	-1.224141
C	10.500564	8.349470	-3.812575
C	9.312110	7.717638	-4.541181
C	7.981074	5.764040	-4.999564
C	7.721799	4.274587	-4.762067
C	7.819943	2.379110	-3.280736
C	8.179237	1.808843	-1.906643
C	9.532111	8.464634	-1.590070
C	10.385249	5.675417	-5.290445
C	6.766813	4.433060	-2.538479
C	2.945260	5.506790	1.783667
C	4.071731	5.998517	2.695634
C	5.397571	7.832795	3.520595
C	5.706436	9.332088	3.510419
C	5.774993	11.411376	2.297927
C	5.552713	12.170735	0.987376
C	4.119135	5.629330	-0.335789
C	2.977810	7.952350	3.626016
C	6.852924	9.443690	1.374680
C	4.310862	12.406929	-1.064294
C	3.184168	11.909691	-1.973636
C	1.868858	10.074478	-2.805876
C	1.561473	8.574924	-2.797951

C	1.474692	6.499697	-1.577122
C	1.698782	5.749057	-0.262290
C	3.136476	12.345832	1.056074
C	4.290352	9.948587	-2.876949
C	0.332609	8.453983	-0.710532
H	9.689686	0.769405	0.166193
H	8.673251	2.172627	0.527283
H	10.781310	2.120407	1.803278
H	11.736960	2.138042	0.314619
H	12.914552	3.854095	0.707331
H	12.258857	4.267330	2.297595
H	11.673833	6.536929	1.541194
H	13.399593	6.167047	1.409240
H	13.299164	8.265281	-0.235480
H	11.596812	8.201918	0.242928
H	11.978643	9.511485	-1.798058
H	12.707060	8.123419	-2.619338
H	10.500244	1.955507	-3.227138
H	10.581520	0.538284	-2.140020
H	11.552791	2.001944	-1.805815
H	8.760659	4.217172	1.118137
H	9.740182	5.642415	1.447998
H	9.786658	4.260656	2.582836
H	13.390373	4.706983	-1.191878
H	14.414846	6.134359	-0.860045
H	13.323609	6.103962	-2.275980
H	10.392796	9.451779	-3.912585
H	11.435855	8.082825	-4.325269
H	9.327133	8.102988	-5.584332
H	8.372410	8.074612	-4.094767
H	7.200786	6.359101	-4.502602
H	7.863047	5.945958	-6.090425
H	8.433140	3.674268	-5.346976
H	6.712779	4.051555	-5.172920
H	6.822092	1.969326	-3.550946
H	8.523677	2.000472	-4.035785
H	8.111406	0.701537	-1.983462
H	7.420811	2.114303	-1.171853
H	9.624590	8.120522	-0.550163
H	9.489901	9.574197	-1.584524
H	8.568642	8.091703	-1.965571
H	11.349917	6.015778	-4.890847
H	10.367599	4.579308	-5.225829
H	10.337432	5.958189	-6.362841
H	6.802027	5.530817	-2.582912
H	5.757758	4.107831	-2.868279

H	6.891522	4.150923	-1.483128
H	3.016959	4.397529	1.749850
H	1.970829	5.739783	2.236159
H	3.951920	5.478736	3.671482
H	5.041907	5.673706	2.291768
H	6.204515	7.285630	3.011514
H	5.419828	7.502770	4.582353
H	4.957177	9.870418	4.108354
H	6.678507	9.465691	4.033680
H	6.750329	11.752759	2.708676
H	5.011055	11.705909	3.031885
H	5.625203	13.255481	1.221296
H	6.373554	11.949846	0.290402
H	4.140438	6.105523	-1.326586
H	4.123630	4.528245	-0.478661
H	5.051839	5.910109	0.173841
H	2.044240	7.691386	3.109834
H	3.013767	9.046938	3.707884
H	2.933460	7.530815	4.651907
H	6.791710	8.352009	1.261855
H	7.836843	9.685034	1.829095
H	6.829028	9.871545	0.362355
H	4.254008	13.517554	-1.051737
H	5.283143	12.152266	-1.509444
H	3.308169	12.421558	-2.952977
H	2.213933	12.238248	-1.574860
H	1.054235	10.622221	-2.311196
H	1.863645	10.401149	-3.868762
H	2.333746	8.036980	-3.366109
H	0.609043	8.433971	-3.354580
H	0.510728	6.140288	-1.999274
H	2.252890	6.215976	-2.299890
H	1.643446	4.663116	-0.495336
H	0.871255	5.958407	0.430566
H	3.117895	11.896302	2.057534
H	3.148314	13.449844	1.173300
H	2.201727	12.065512	0.552880
H	5.221147	10.192204	-2.346729
H	4.254398	8.853827	-2.962919
H	4.353854	10.373640	-3.900566
H	0.382365	9.544629	-0.593769
H	-0.626452	8.202985	-1.210307
H	0.317551	8.016857	0.296504

K-HMHC (Na) – Boat Conformation

K	1.415921	-2.605097	-3.135161
Na	-1.450097	-3.601020	-0.535488
N	1.981422	-4.365871	-5.662870
N	-0.311040	-2.270493	-5.554143
N	0.386585	0.145169	-3.730393
N	3.385251	-0.299670	-3.292249
N	3.727821	-2.527796	-1.200811
N	3.143775	-5.063028	-2.875455
C	3.065606	-3.869914	-6.515379
C	-1.567680	-2.818372	-5.020466
C	-0.130328	0.602111	-2.432791
C	3.437311	-5.757466	-4.137437
C	2.296879	-5.712860	-5.155615
C	0.712302	-4.406300	-6.410074
C	0.121876	-3.032979	-6.734731
C	-0.461611	-0.847852	-5.898978
C	-0.719873	0.068471	-4.701406
C	4.006065	-0.679054	-4.564076
C	3.148524	-1.958540	0.023983
C	2.220681	-5.854108	-2.048300
C	1.449064	1.028736	-4.228368
C	2.736175	1.015790	-3.399560
C	4.376699	-0.297881	-2.204991
C	4.810555	-1.687050	-1.729622
C	4.220046	-3.888488	-0.921124
C	4.392531	-4.776446	-2.155013
H	3.998137	-3.797650	-5.944395
H	3.242458	-4.529642	-7.391162
H	2.832054	-2.864542	-6.883201
H	-2.412841	-2.679121	-5.727599
H	-1.823539	-2.354001	-4.058619
H	-1.457219	-3.888648	-4.810849
H	-0.574060	1.618176	-2.489121
H	0.667824	0.613495	-1.681673
H	-0.891383	-0.105837	-2.074328
H	3.685750	-6.825805	-3.960851
H	4.337877	-5.302995	-4.572015
H	2.561342	-6.400793	-5.988229
H	1.383543	-6.115211	-4.696454
H	-0.007214	-4.976323	-5.806969
H	0.829318	-4.959240	-7.367499
H	0.864184	-2.429576	-7.274603
H	-0.720218	-3.190248	-7.442148
H	0.452607	-0.533076	-6.422664
H	-1.301037	-0.695896	-6.612826

H	-1.606030	-0.285517	-4.161148
H	-0.980329	1.075081	-5.092817
H	3.249568	-0.732587	-5.356371
H	4.789195	0.042975	-4.878736
H	4.466188	-1.670686	-4.481391
H	3.894429	-1.864315	0.841052
H	2.723565	-0.967850	-0.173871
H	2.319558	-2.596011	0.363034
H	1.316550	-6.101749	-2.616999
H	2.686581	-6.797431	-1.692416
H	1.873200	-5.276059	-1.181463
H	1.677484	0.741076	-5.263770
H	1.100077	2.084756	-4.269684
H	2.512866	1.359334	-2.381897
H	3.422817	1.770186	-3.841154
H	5.296351	0.253484	-2.497789
H	3.943615	0.252834	-1.361086
H	5.603751	-1.534379	-0.963692
H	5.286883	-2.224887	-2.560651
H	5.193011	-3.860161	-0.385219
H	3.507577	-4.360300	-0.234019
H	4.886285	-5.713901	-1.815715
H	5.084579	-4.301464	-2.865189

K-HMHC (Na) – Less Preferred Boat Conformation

K	2.386095	-2.363378	-4.086921
Na	5.654820	-1.672377	-7.102027
N	3.117677	-5.014164	-3.026228
N	3.595284	-2.518617	-1.216608
N	3.427048	0.154322	-2.873770
N	0.411059	0.041806	-3.778979
N	-0.134857	-2.389060	-5.656796
N	1.994128	-4.714485	-5.976680
C	4.392105	-4.632064	-2.387152
C	4.252162	-3.839416	-1.086196
C	4.617577	-1.459713	-1.344264
C	4.072406	-0.046028	-1.567423
C	2.448094	1.251262	-2.853378
C	1.370519	1.154452	-3.936933
C	2.249625	-5.756947	-2.108326
C	2.711156	-2.276043	-0.072040
C	4.442625	0.396368	-3.908202
C	-0.518921	0.011039	-4.931098
C	-0.146449	-0.966270	-6.048250
C	0.188041	-3.212699	-6.840684
C	0.622727	-4.641997	-6.503228

C	2.225910	-5.939946	-5.197201
C	3.407850	-5.820679	-4.230063
C	-0.337900	0.153605	-2.523201
C	-1.418453	-2.802620	-5.082785
C	2.964328	-4.623723	-7.077064
H	4.971945	-4.051290	-3.123834
H	4.998215	-5.533631	-2.146473
H	3.688184	-4.436743	-0.359632
H	5.266402	-3.731776	-0.656609
H	5.283604	-1.732239	-2.177248
H	5.250334	-1.426414	-0.429464
H	3.347774	0.194759	-0.779271
H	4.921054	0.656175	-1.429102
H	2.946882	2.235770	-2.976175
H	1.971088	1.273980	-1.865205
H	0.830030	2.127056	-3.949434
H	1.850583	1.048542	-4.921649
H	1.906570	-5.111413	-1.291278
H	2.754160	-6.644679	-1.671153
H	1.355265	-6.103125	-2.638590
H	1.998520	-3.104841	0.019771
H	2.126117	-1.361688	-0.222518
H	3.260891	-2.180189	0.887972
H	5.144453	-0.446183	-3.978452
H	5.024028	1.322345	-3.707384
H	3.984234	0.480810	-4.902925
H	-0.606525	1.014147	-5.389986
H	-1.522807	-0.230001	-4.561239
H	-0.870133	-0.792467	-6.875629
H	0.851926	-0.721075	-6.445781
H	-0.678672	-3.254617	-7.534504
H	1.000623	-2.709484	-7.384601
H	0.508509	-5.264851	-7.415505
H	-0.056277	-5.067099	-5.752087
H	1.310176	-6.166416	-4.634780
H	2.406496	-6.811083	-5.862213
H	4.252367	-5.346164	-4.751470
H	3.743140	-6.840795	-3.945592
H	-1.071609	-0.659529	-2.462982
H	0.332594	0.048128	-1.662837
H	-0.877555	1.118999	-2.424486
H	-1.406876	-3.879398	-4.879489
H	-1.595934	-2.298965	-4.125500
H	-2.273330	-2.590787	-5.759764
H	2.840188	-3.684193	-7.630190
H	2.861676	-5.477145	-7.782281

H	3.991286	-4.600979	-6.690356

15-Crown-5

O	5.121819	-1.196222	-0.063306
O	2.867092	-2.586254	1.286398
O	5.255717	1.541516	0.834650
O	2.496027	2.145321	0.248397
O	1.031914	-0.315445	0.982818
C	5.068337	-2.557165	0.352953
C	6.317135	-0.537965	0.339177
C	3.648228	-3.057376	0.186626
C	1.459071	-2.669884	1.073341
C	0.879190	-1.525738	0.252187
C	6.208502	0.927580	-0.026073
C	4.776041	2.800490	0.370762
C	3.386031	3.020533	0.931687
C	1.201690	2.063730	0.836789
C	0.519519	0.824336	0.294244
H	5.360116	-2.653062	1.414285
H	5.754495	-3.178800	-0.254553
H	3.642584	-4.163765	0.165420
H	3.258926	-2.688483	-0.777043
H	7.200157	-0.971591	-0.169545
H	6.462507	-0.635007	1.430268
H	7.204078	1.400996	0.075778
H	5.887685	1.014041	-1.079155
H	1.007487	-2.645430	2.073957
H	1.185435	-3.627985	0.592841
H	-0.191307	-1.746121	0.062820
H	1.384959	-1.444189	-0.727958
H	4.719682	2.812538	-0.731198
H	5.442352	3.621877	0.694773
H	3.093982	4.078873	0.789532
H	3.389855	2.801255	2.014115
H	0.601056	2.960964	0.594113
H	1.276093	1.979725	1.935125
H	0.727650	0.754680	-0.787704
H	-0.575016	0.902320	0.434840

Na-15-Crown-5_2

Na	0.798131	1.886151	3.650741
O	-0.273280	1.647382	1.427431
O	-1.100280	3.586701	3.294621
O	1.613387	4.297241	3.313413
O	1.738621	-0.327723	2.116511
O	3.002587	2.138584	2.370422

O	-1.170502	0.392858	4.467792
O	0.313843	2.273627	6.109021
O	3.272656	2.511222	5.831023
O	0.770126	-1.465933	5.620042
O	3.375852	-0.319571	6.053390
C	-0.729694	2.918114	0.946691
C	-1.680448	3.500191	1.978403
C	-0.663362	4.892079	3.712756
C	0.646454	5.327371	3.081657
C	2.812159	4.502561	2.552069
C	0.451479	0.923200	0.423975
C	0.837522	-0.425919	0.999590
C	3.116775	-0.146990	1.726079
C	3.719505	3.317809	2.772614
C	3.754243	0.931517	2.572131
C	-1.768063	1.276670	5.435018
C	-0.838493	1.562096	6.596332
C	1.199739	2.578160	7.198073
C	2.437798	3.296373	6.693075
C	4.358964	1.819420	6.457175
C	-1.401473	-1.004690	4.739444
C	-0.139662	-1.821932	4.581886
C	1.995416	-2.194108	5.534944
C	4.006209	0.465533	7.058474
C	2.971473	-1.604926	6.526979
H	-1.282565	2.792963	-0.003812
H	0.130141	3.583101	0.754615
H	-2.548182	2.832444	2.074277
H	-2.034373	4.487264	1.638497
H	-0.539509	4.808901	4.800725
H	-1.439626	5.648111	3.503714
H	0.971717	6.280448	3.542963
H	0.531472	5.500503	1.996375
H	1.342965	1.494004	0.110477
H	-0.190012	0.764107	-0.463487
H	1.267239	-1.055931	0.203336
H	-0.064702	-0.922238	1.381472
H	2.559152	4.600636	1.480496
H	3.327301	5.425481	2.876368
H	3.996009	3.245499	3.834437
H	4.630456	3.439713	2.159032
H	3.179221	0.142198	0.666497
H	3.662673	-1.096402	1.862406
H	4.799795	1.064178	2.241376
H	3.754361	0.651885	3.638296
H	-2.715172	0.861563	5.814539

H	-1.987376	2.208297	4.898443
H	-0.514080	0.622943	7.077507
H	-1.370178	2.180529	7.344480
H	1.465992	1.648195	7.727037
H	0.684514	3.245194	7.916788
H	3.011086	3.658748	7.563991
H	2.130314	4.168637	6.097656
H	-2.163804	-1.380424	4.034946
H	-1.781568	-1.135622	5.763808
H	0.324350	-1.644910	3.596761
H	-0.414384	-2.893223	4.649042
H	5.099697	1.664056	5.659312
H	4.823591	2.448989	7.236383
H	3.342024	0.570439	7.936069
H	4.946583	-0.007059	7.408277
H	2.413903	-2.124328	4.515487
H	1.830347	-3.262384	5.770997
H	3.844697	-2.274886	6.634369
H	2.479243	-1.515171	7.511687

K-15-Crown-5_2

K	1.116486	1.438263	4.150287
O	-0.134384	1.491866	1.429292
O	-0.730106	3.542339	3.357791
O	2.098845	4.077928	3.331893
O	1.995508	-0.521271	1.912319
O	3.374232	1.915937	2.118917
O	-1.372683	0.298032	4.789482
O	-0.465602	2.480932	6.451905
O	2.233757	2.043504	6.951289
O	0.955048	-1.190912	5.395850
O	3.492179	0.038345	5.556706
C	-0.428664	2.827993	1.007620
C	-1.313354	3.493571	2.046116
C	-0.164059	4.800491	3.753874
C	1.166895	5.141663	3.105106
C	3.295617	4.255525	2.560626
C	0.528464	0.761908	0.391777
C	0.949715	-0.593196	0.928530
C	3.305086	-0.331632	1.337707
C	4.137530	3.004299	2.642727
C	4.093045	0.676929	2.144532
C	-2.246530	1.253037	5.411843
C	-1.707137	1.824049	6.713855
C	0.165775	2.951118	7.649037
C	1.611980	3.268443	7.343323

C	3.634583	2.156963	6.708999
C	-1.418140	-1.007609	5.379943
C	-0.230903	-1.801970	4.882984
C	2.148619	-1.905685	5.049289
C	4.184338	0.749404	6.595974
C	3.290454	-1.348180	5.869174
H	-0.981907	2.814167	0.048305
H	0.510238	3.386413	0.851512
H	-2.238138	2.906280	2.145431
H	-1.583616	4.504778	1.698144
H	-0.042426	4.715980	4.841378
H	-0.871476	5.624824	3.551478
H	1.537320	6.087580	3.548173
H	1.047677	5.308343	2.019106
H	1.404767	1.327346	0.029962
H	-0.159441	0.610568	-0.462726
H	1.249010	-1.242812	0.088837
H	0.093054	-1.062023	1.431318
H	3.031331	4.450296	1.505935
H	3.879107	5.115902	2.938805
H	4.439601	2.799211	3.684947
H	5.059125	3.157662	2.048926
H	3.220383	0.028599	0.301838
H	3.844226	-1.295408	1.325999
H	5.090552	0.793352	1.680847
H	4.229937	0.336569	3.185217
H	-3.241563	0.811817	5.595907
H	-2.349645	2.066323	4.683295
H	-1.557849	1.032986	7.470667
H	-2.453685	2.536532	7.115471
H	0.127184	2.168882	8.426431
H	-0.343499	3.855328	8.030947
H	2.091881	3.680436	8.250544
H	1.692393	4.023861	6.540858
H	-2.353375	-1.521400	5.091898
H	-1.371437	-0.949877	6.479569
H	-0.216109	-1.820722	3.778712
H	-0.317589	-2.843674	5.242787
H	3.823148	2.747901	5.794625
H	4.146149	2.667207	7.546908
H	4.029397	0.238760	7.558990
H	5.269162	0.785095	6.390091
H	2.342943	-1.816110	3.968046
H	2.042101	-2.977310	5.299445
H	4.203467	-1.936078	5.661909
H	3.042000	-1.450151	6.938117

K-15-Crown-5 (Na)

K	0.914475	2.507104	4.606203
Na	0.117072	1.821513	8.144973
O	-0.433063	2.265398	1.999505
O	0.178640	4.877488	3.064511
O	2.908148	4.568961	4.027219
O	1.246257	0.130086	2.981677
O	3.367330	1.987706	2.882634
C	-0.573158	3.439107	1.196999
C	-0.861459	4.617995	2.113947
C	1.262521	5.664974	2.556587
C	2.288076	5.812710	3.670047
C	4.045114	4.243778	3.217073
C	-0.183317	1.092735	1.219403
C	0.088258	-0.069789	2.162665
C	2.487497	-0.148327	2.319599
C	4.405596	2.793376	3.448831
C	3.596399	0.583456	3.043744
H	-1.418796	3.330109	0.491361
H	0.344549	3.604515	0.602881
H	-1.754559	4.394074	2.712129
H	-1.066255	5.513672	1.502537
H	1.717806	5.193409	1.667702
H	0.894939	6.667412	2.265741
H	3.045427	6.559356	3.377551
H	1.781696	6.166760	4.578064
H	0.674229	1.264682	0.543416
H	-1.064405	0.850114	0.595431
H	0.170311	-1.001932	1.578087
H	-0.750790	-0.175621	2.863200
H	3.825365	4.391326	2.145858
H	4.900279	4.886060	3.494048
H	4.506826	2.596745	4.531082
H	5.373148	2.577857	2.958783
H	2.462818	0.191585	1.271402
H	2.686139	-1.234634	2.335047
H	4.567660	0.294171	2.601975
H	3.602486	0.311038	4.114073

K-15-Crown-5_2 (Na) Axial

K	1.150814	1.413815	4.239045
Na	0.613600	-1.625009	9.618604
O	-0.544862	2.075116	2.029831
O	0.292370	4.241078	3.845613

O	3.041853	3.955150	3.136592
O	1.132582	-0.347774	1.818135
O	3.260571	1.499158	1.775782
O	-0.944513	-0.534502	4.475674
O	-1.034409	1.902047	6.078108
O	1.639922	2.417906	6.794631
O	1.708954	-1.067763	5.271268
O	3.688273	0.877474	5.759061
C	-0.762651	3.456560	1.730379
C	-0.888942	4.235960	3.030397
C	1.179697	5.355852	3.686013
C	2.326365	5.116070	2.716808
C	4.136415	3.643940	2.265499
C	-0.449475	1.280068	0.841241
C	-0.162357	-0.162570	1.225860
C	2.204181	-0.417177	0.867149
C	4.407803	2.157172	2.321611
C	3.467903	0.114318	1.509238
C	-2.146005	0.080644	4.966050
C	-1.972795	0.835351	6.273526
C	-0.666538	2.518215	7.324204
C	0.592125	3.333512	7.122744
C	2.940413	2.995779	6.677400
C	-0.577325	-1.726981	5.198758
C	0.837438	-2.116469	4.832114
C	3.083782	-1.440254	5.348941
C	3.941179	1.859010	6.782537
C	3.792126	-0.467424	6.265605
H	-1.706456	3.580618	1.163298
H	0.060601	3.845757	1.104975
H	-1.667736	3.767125	3.648495
H	-1.200782	5.267918	2.795928
H	1.598878	5.546872	4.684920
H	0.624406	6.255957	3.370938
H	2.977396	6.013077	2.727968
H	1.958832	4.980989	1.683258
H	0.343081	1.681818	0.184503
H	-1.404756	1.316703	0.283224
H	-0.282667	-0.802373	0.335076
H	-0.877891	-0.485236	1.993066
H	3.880639	3.914529	1.226857
H	5.040319	4.205761	2.562597
H	4.588906	1.823644	3.358279
H	5.307102	1.939584	1.716745
H	1.982906	0.191844	-0.023657
H	2.358199	-1.463343	0.546587

H	4.315028	-0.029749	0.812905
H	3.691987	-0.436483	2.440269
H	-2.943167	-0.672240	5.097088
H	-2.453220	0.779029	4.175530
H	-1.618440	0.168774	7.080919
H	-2.959523	1.235128	6.577504
H	-0.473302	1.740160	8.083889
H	-1.479266	3.175171	7.684728
H	0.824301	3.866269	8.062296
H	0.449186	4.071469	6.314170
H	-1.266823	-2.550899	4.939816
H	-0.616886	-1.561846	6.289534
H	0.942044	-2.272097	3.744083
H	1.075746	-3.054983	5.362583
H	3.046886	3.536874	5.720229
H	3.134509	3.704416	7.502437
H	3.832452	1.395086	7.774037
H	4.966729	2.255719	6.686017
H	3.541237	-1.464133	4.342276
H	3.186324	-2.444494	5.798268
H	4.851872	-0.766944	6.359467
H	3.313932	-0.528584	7.258210

K-15-Crown-5_2 (Na) Equatorial

K	1.172933	1.751272	3.808135
Na	6.432344	-0.087757	4.215899
O	2.599451	0.817436	6.134632
O	0.740854	-1.166121	5.515072
O	1.747092	3.507790	6.009220
O	-0.803714	2.113217	6.011897
O	-1.452812	-0.079057	4.047007
O	3.117995	1.803916	1.588865
O	1.254458	-0.248833	1.755923
O	2.617727	4.310436	2.616057
O	-0.170204	4.298597	3.290980
O	-0.367369	2.172178	1.296666
C	2.789143	-0.530705	6.583059
C	3.229084	1.752900	7.008746
C	2.138967	-1.467180	5.596391
C	0.080846	-1.907729	4.490321
C	-1.365241	-1.460182	4.446541
C	3.078877	3.150424	6.429535
C	0.766682	3.618389	7.047439
C	-0.094453	2.376107	7.228279
C	-1.792719	1.094715	6.196823
C	-2.359456	0.691379	4.844179

C	3.440644	0.615661	0.856647
C	4.166650	2.788762	1.590942
C	2.619451	-0.546723	1.404709
C	0.339431	-0.047988	0.672874
C	0.194983	1.399820	0.226950
C	3.547171	4.167696	1.536576
C	1.869411	5.517932	2.532071
C	0.690765	5.428743	3.491527
C	-1.004516	4.340898	2.127406
C	-0.461170	3.552724	0.943199
H	2.347925	-0.657205	7.588954
H	3.869802	-0.767022	6.625979
H	2.300775	-2.509501	5.929399
H	2.624899	-1.348806	4.611175
H	4.313749	1.538227	7.088655
H	2.787652	1.680466	8.021613
H	3.455353	3.881802	7.166972
H	3.687493	3.232867	5.518524
H	0.111787	-2.993522	4.704436
H	0.561186	-1.728173	3.513220
H	-1.816674	-1.606521	5.439630
H	-1.918165	-2.082072	3.721213
H	0.111372	4.453557	6.757973
H	1.241653	3.869141	8.010915
H	-0.808997	2.577073	8.050001
H	0.514112	1.497920	7.497764
H	-2.612279	1.479790	6.833940
H	-1.344769	0.219002	6.697374
H	-2.579302	1.595019	4.260019
H	-3.305574	0.144688	4.999776
H	3.260400	0.782499	-0.222065
H	4.503914	0.342117	0.996579
H	2.656681	-1.385177	0.686037
H	3.082744	-0.877250	2.347144
H	4.786060	2.654595	2.494480
H	4.823812	2.672713	0.714472
H	4.348671	4.927364	1.604665
H	3.016257	4.308269	0.576041
H	-0.636144	-0.396810	1.041206
H	0.628345	-0.660637	-0.197949
H	-0.477952	1.426955	-0.652186
H	1.170164	1.823425	-0.062047
H	1.526069	5.685190	1.493829
H	2.487908	6.391760	2.816928
H	0.112507	6.368551	3.434995
H	1.073566	5.302129	4.511804

H	-1.186738	5.382340	1.811497
H	-1.966750	3.900642	2.427840
H	0.529954	3.927938	0.636402
H	-1.156527	3.684595	0.090382
